# A novel network-based approach for discovering dynamic metabolic biomarkers in cardiovascular disease

**DOI:** 10.1371/journal.pone.0208953

**Published:** 2018-12-11

**Authors:** Christian Baumgartner, Verena Spath-Blass, Verena Niederkofler, Katharina Bergmoser, Sonja Langthaler, Alexander Lassnig, Theresa Rienmüller, Daniela Baumgartner, Aarti Asnani, Robert E. Gerszten

**Affiliations:** 1 Institute of Health Care Engineering with European Testing Center of Medical Devices, Graz University of Technology, Graz, Austria; 2 Department of Pediatric Cardiology, Medical University of Graz, Graz, Austria; 3 Cardiovascular Medicine, Beth Israel Deaconess Medical Center and Harvard Medical School, Boston, Massachusetts, United States of America; University of Florida, UNITED STATES

## Abstract

Metabolic biomarkers may play an important role in the diagnosis, prognostication and assessment of response to pharmacological therapy in complex diseases. The process of discovering new metabolic biomarkers is a non-trivial task which involves a number of bioanalytical processing steps coupled with a computational approach for the search, prioritization and verification of new biomarker candidates. Kinetic analysis provides an additional dimension of complexity in time-series data, allowing for a more precise interpretation of biomarker dynamics in terms of molecular interaction and pathway modulation. A novel network-based computational strategy for the discovery of putative dynamic biomarker candidates is presented, enabling the identification and verification of unexpected metabolic signatures in complex diseases such as myocardial infarction. The novelty of the proposed method lies in combining metabolic time-series data into a superimposed graph representation, highlighting the strength of the underlying kinetic interaction of preselected analytes. Using this approach, we were able to confirm known metabolic signatures and also identify new candidates such as carnosine and glycocholic acid, and pathways that have been previously associated with cardiovascular or related diseases. This computational strategy may serve as a complementary tool for the discovery of dynamic metabolic or proteomic biomarkers in the field of clinical medicine.

## Introduction

Omics biomarkers are gaining importance in biomedical research and clinical applications, and allow for the identification, characterization, classification and monitoring of disease [1, 2, 3]. In this regard, biomarkers, which are defined as “objectively measurable and quantifiable indicators of biological or pathological processes” [4, 5], are widely used in a clinical setting as they may serve as predictors of the disease state and progression. For example, the diagnosis of complex cardiovascular diseases such as myocardial infarction typically relies on the measurement of myocardial necrosis markers such as the proteins creatine kinase, muscle and brain subunits (CK-MB) and cardiac troponins [6, 7, 8, 9, 10], which reach their peak values within 12 to 24 hours after an event and, consequently, are much less suitable to serve as biomarkers for very early diagnosis [11]. Although newer assays for cardiac injury, e.g., high-sensitivity troponins, can diagnose myocardial injury at earlier time points, they are limited by decreased specificity for myocardial infarction [12]. Therefore, the search for new biomarkers that can aid in the early diagnosis or even prognosis of myocardial injury is of great clinical interest [13, 14]. In 2008, as the first study worldwide we investigated metabolite level changes in the human blood with the objective of identifying early metabolic biomarker candidates using a “planned” myocardial injury (PMI) model. Early alterations in purine and pyrimidine metabolism, such as hypoxanthine and adenosine monophosphate (AMP), as well as transmyocardial enrichment of metabolites related to myocardial anaerobic metabolism, such as lactic acid and succinic acid, were associated with myocardial injury [15].

Bioinformatic-driven search strategies for discovering new biomarker candidates are under continuous research and development, in large part due to innovations in high-throughput technologies resulting in the generation of large datasets. Thus, both the discovery and validation of putative biomarker candidates require interdisciplinary collaboration between various fields of expertise in biomedical research, including bioinformatics for treating all relevant data-driven tasks [16, 17, 18]. The procedure leading to the discovery of new biomarkers is highly complex and requires a number of different steps from the bioinformatics perspective, including well-defined concepts for data management, information retrieval and data mining to guarantee the validity and generalizability of findings [19, 20].

A commonly used computational approach for information retrieval is inferring networks of genes, proteins, metabolites, cells and other biological entities from complex biological datasets. Dynamic network representations play an especially important role in predicting changes in biochemical pathways, identifying correlations between biomolecules, or investigating pathway dynamics when kinetic information is available [21, 22]. A first approach using dynamic networks to identify candidate metabolic biomarkers of physical activity was introduced by our group in the work of Netzer et al. [23].

Here we present a new three-step computational strategy for characterizing the dynamics of longitudinal metabolite levels through an appropriate graph representation. To thoroughly investigate changes in metabolite interactions using time-series data, the network inference approach introduced by Netzer et al. [23] was extended by transforming the inferred networks, created at various measurement time points, into a single superimposed, combined graph representation and visualization. This new dynamic network approach was tested and evaluated using clinical time series data collected in the study of Lewis et al. [15] for the purpose of identifying and verifying early metabolic biomarker candidates in myocardial injury.

## Materials and methods

The proposed network-based strategy for the discovery and prioritization of new metabolic biomarker candidates takes into consideration the kinetic information of analytes as an additional degree of data complexity, represented by longitudinal mass spectrometry (MS) data. The kinetic network is built up upon a three-step modality, including (i) data preprocessing of raw data, (ii) the preselection of a subset of metabolites with enhanced discriminatory and predictive ability by using an appropriate feature selection paradigm and (iii) finally, the network inference by combining multiple single networks at different measurement time points into one combined dynamic graph representation.

### Metabolic data

In this study, changes in metabolite levels were measured in a cohort of 17 patients undergoing alcohol septal ablation for the treatment of symptomatic hypertrophic obstructive cardiomyopathy (HOCM), serving as a clinically accepted model for “planned” myocardial injury (PMI). Blood samples were drawn from the patients over a defined period of time before (baseline at t_0_) and, according to the study protocol, at ten minutes [t_10_ (n = 17)], one hour [t_60_ (n = 17)], two hours [t_120_ (n = 13)] and four hours [t_240_ (n = 8)] after injury, allowing patients to serve as their own biological controls and thus permitting kinetic analyses of circulating metabolites according to the selected longitudinal cohort study design. Inclusion criteria of patients, the detailed patient characteristics as well as the protocol of the MS analysis can be found in Lewis et al. [15]. All protocols in this study for obtaining blood from patients were approved by the Massachusetts General Hospital Institutional Review Board, and all subjects gave written informed consent.

The detailed procedure of metabolite qualification and quantitation is also described in [15], including the following steps (i) blood sample processing, (ii) metabolite selection for analysis platform, (iii) HPLC and MS analysis, and (iv) LC-MS analyses with isotope standards for absolute analyte quantitation. All related information on this procedure can be found in the Supplemental data, therein (https://www.jci.org/articles/view/35111/sd/1). Briefly, three HPLC columns for separating the metabolite classes of sugars and ribonucleotides, organic acids, and amino acids were aligned in sequence with a triple quadrupole mass spectrometer using a turbo ion spray LC/MS interface. Targeted MS/MS analysis using selective reaction monitoring (SRM) conditions was performed allowing for measurement of a total of 210 metabolites for each sample. Metabolite quantitation expressed in MS intensity units (IU) was carried out by integrating peak areas for parent/daughter ion pairs. In addition, for a subset of metabolites, for which isotope labeled standards were available, an absolute quantitation of analytes in plasma (ng/ml) was performed.

For computational analysis of time series data, metabolite levels (IU) at time t_10_ = 10 min, t_60_ = 60 min, t_120_ = 120 min and t_240_ = 240 min after myocardial injury were normalized to the baseline level at time t_0_ before alcohol septal ablation. Thus, patients in this study served as their own biological controls. The data used in this study are available in S1, S2 and S3 Datasets.

### Data preprocessing and subset selection

As a first step, data preprocessing was carried out to provide quality-assured data for network construction, including: (i) technical validation of data with plausibility checks where for each sample all metabolite peaks were manually reviewed for peak quality in a blinded manner according to the internal lab standards, with those with poor peak quality labeled as “missing values” in the final dataset, (ii) outlier detection where a common statistical model based on interquartile ranges (IQR) was used, leading to a removal of 4% of MS data. We defined an outlier as an observation outside the range [q25 –k ⋅ IQR; q75 + k ⋅ IQR], with q25, q75 = first and third quartiles, IQR = q75 –q25 and set the parameter k = 3 to only remove ‘extreme’ outliers in the data, (iii) handling of missing values, which means that analytes with more than 60% missing values in a subset of two time points were excluded. In case of less than 60% missing values, these values were replaced by the median of analyte levels at the given time point. Consequently, from the initial set of 210 analytes, 170 analytes were included for further consideration after handling of missing values. Finally, a set of 71 fully identified and annotated metabolites was used for dynamic network construction. The three datasets including the original (S1 Dataset), preprocessed (S2 Dataset) and fully annotated dataset (S3 Dataset) are available in the Supporting information.

Secondly, a univariate feature selection method, termed paired Biomarker Identifier (pBI) was applied to the data to preselect a subset of metabolites based on the pBI score as starting point for inferring the networks [24]. The pBI score was developed to select key metabolites from the data according to the underlying paired data characteristics (e.g. data at baseline t_0_ vs. after 10 minutes t_10_) and to prioritize them into classes of weak, moderate and strong predictors. In detail, the pBI score combines the following terms: a scaling factor λ, the discriminatory ability DA*, a biological effect term calculated as the median percent change in metabolite levels Δ_change_ at time t_x_ versus baseline t_0_, divided by the coefficient of variation (CV) in the normalized data, and the direction of level change given by the sign function:

$$pBI=\lambda \cdot DA^{*}\cdot \sqrt{\frac{\left|\Delta_{change}\right|}{\left|CV\right|}}\cdot sign\left(\Delta_{change}\right)$$ (1)

with $\Delta_{change}=\{\begin{array}{cc} \Delta & if\Delta \geq 1\\ -\frac{1}{\Delta } & else \end{array}$

Using this method, metabolites were ranked with respect to their absolute pBI scores at each point in time and scores of the same metabolites higher than a defined cutoff (in this study |pBI| > q75 including only moderate and strong predictors) at each measurement were added up and ranked again from highest to lowest. Subsequently, a subset of 20 top-ranked and fully annotated metabolites was included for final network construction. For this step we propose a well-defined classification scheme, prioritizing analytes into strong (|pBI| > q90 i.e. 90% percentile), moderate and strong (|pBI| > q75 i.e. third quartile) and weak, moderate and strong predictors (|pBI| > q50 i.e. second quartile). For reasons of clarity and comprehensibility in dynamic network analysis the threshold can be adjusted accordingly.

### Dynamic network construction

A network graph, representing the preselected subset of candidate metabolites of the given dataset and their interaction between each other, is inferred by adapting the BiomarkR package by Netzer et al. [23] using the programming language R [25].

Basically, a network graph G is defined as a set of vertices or nodes V, which are connected by edges E: G = (V, E). For inferring the metabolic network, three steps are required: (i) calculating ratios R between metabolites M, which represent chemical interactions where r_ij_ = |log2(m_i_/m_j_)| with i > j, and m ∈ M, r ∈ R, (ii) computing the pBI scores s_ij_, s ∈ S on the logarithmic ratios R and finally (iii) constructing a graph G with | s_ij_ |> τ for i, j ∈ 1, …, |M|. Note that the ratio r ∈ R represents a pathway reaction of the form A → B, where a reactant A is metabolized into a product B, taking into account single or multiple reaction paths. For graph construction, the nodes V are defined as the given analytes M. The edges E represent the chemical interaction of analyte pairs in the network, i.e. the analytes level ratios. An edge is constructed if the pBI score of the binary logarithm ratios r of analyte levels (now referred to as pBI*) is higher than a defined threshold value τ. The resulting graphs are visualized using the igraph R package [26].

#### Threshold determination

**Fig 1:**
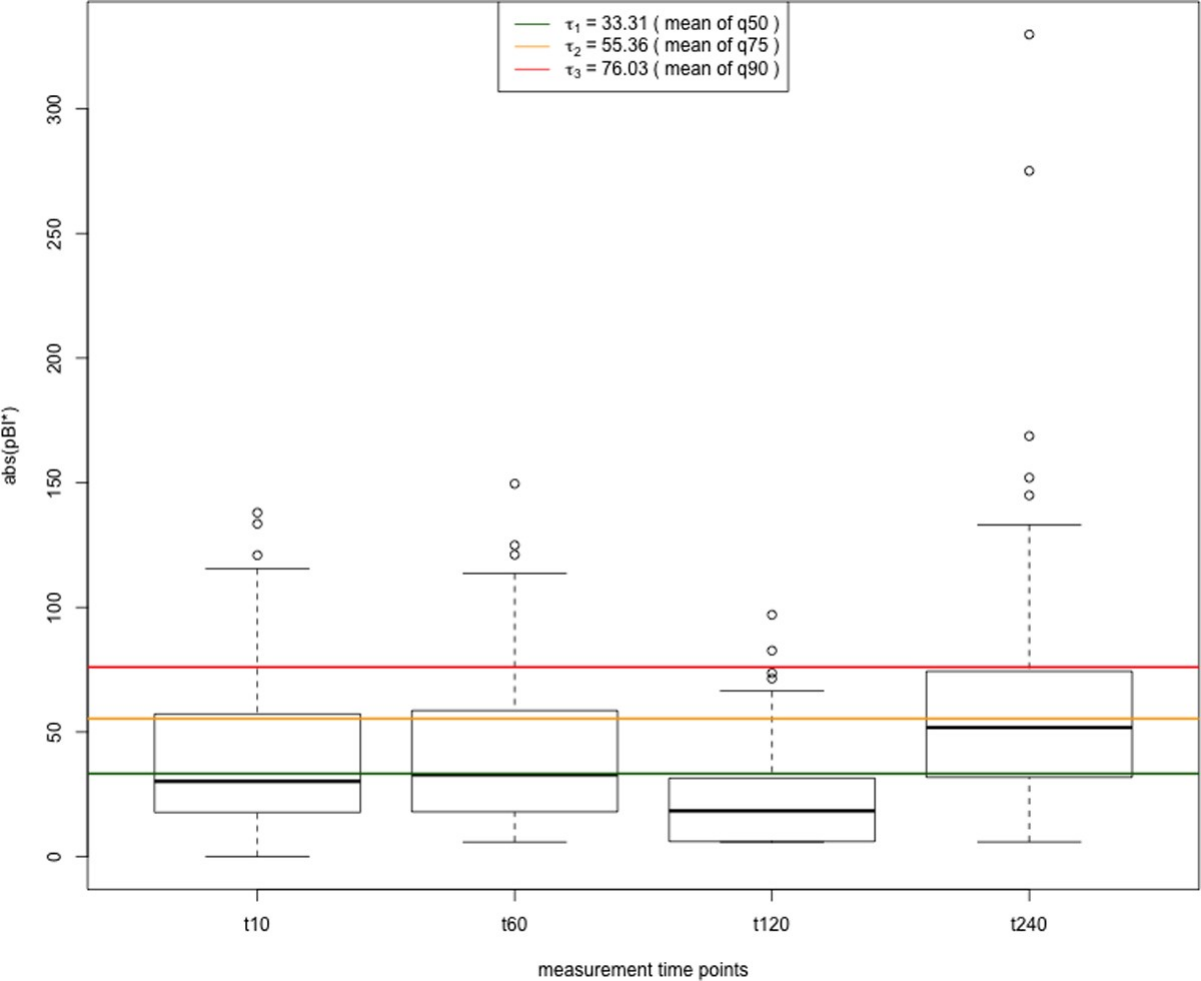
Boxplot of absolute pBI* scores of analyte level ratios r with four measurement points t_i_ [t_10_, t_60_, t_120_, t_240_]. The dynamic thresholds based on the quartiles/percentiles can be derived from the respective value ranges indicated by the boxplot diagrams. The horizontal yellow line represents the averaged static threshold value (τ_s_ = τ_2_ = 55.36), calculated as mean value of the dynamic cutoff values τ_i,2_ (= q75) from the four time points. In addition, the thresholds for q50 (weak, moderate and strong predictors) and q90 (only strong predictors) are visualized in green and red, respectively.

There are different approaches to set the threshold value τ in order to specify the impact of the network edges for different network representations and thus to highlight the hidden information in the data. A statistical model based on interquartile ranges is used to dynamically set the threshold value τ. The absolute values of the pBI* score at each measurement point are considered for quartile/percentile calculation. The calculated quartile values serve as cutoff values, where q50 is again the threshold to classify weak, moderate and strong predictors, q75 for including moderate and strong predictors, and q90 only for strong predictors. Consequently, different dynamic thresholds τ_i,j_ for every measurement point at t_10_, t_60_, t_120_ and t_240_ can be chosen, where i stands for the selected time point t_i_ with i = {1, 2, 3, 4} in this study, and j for the selected quartile/percentile q_j_ with j = {1, 2, 3}, representing q50, q75 and q90 (see also Fig 1). The dynamic thresholding approach based on interquartile ranges provides a general way to work with various metabolite levels regardless of their value range. Alternatively, a static threshold value τ_s_ which is calculated as the mean value over all measurements t_i_ can be applied for constructing the combined, superimposed network graph (see Fig 1, green, yellow and red lines). Note that for all metabolite-to-metabolite ratios, yielding a pBI* score higher than the respective threshold value τ_s_ (static) or respectively τ_i,j_ (dynamic), an edge between the two metabolites is created.

#### Combined network plots and edge weighting

A graph representation, highlighting the dynamics in the given data, is introduced by fusing multiple networks created at the different measurement points. These networks are superimposed, where the nodes are layered upon each other and all edges occurring in any network are taken into account and weighted accordingly, providing a single combined graph representation. Here, two different concepts for calculating the edge weights are implemented:

The first concept–referred to as degree-based (discrete) weighting–is based on combining the adjacency matrices of all graphs. To get an idea of how often two metabolites show a connection over different measurement times, all adjacency matrices are summed up. This results in a combined adjacency matrix, in which each entry a_ij_ can take on values in the range of zero to the number of networks combined. Thus, if n graphs of n different time points are combined, an entry a_ij_ represents a discrete value x with x = {0, 1, 2, …, n-1, n}. These entry values are rescaled to yield a value in the range of [0,1] to ensure that the weight distribution is independent from the total number of graphs combined. The weight values are thus assigned as edge weights to the combined graph. For visualization of the combined plot, the edge weights are displayed as lines with different widths within the network graph. The edges with the smallest width indicate a connection that only occurs in one of the networks at the time points t_i_. In contrast, those edges with the highest weights are shown as the thickest lines. Hence, the thickness of an edge serves as an indicator for the frequency of its occurrence in all of the networks combined.

The second approach for calculating the edge weights, termed score-based (continuous) weighting, dynamically weights the edges based on the pBI* scores of the logarithmic metabolite level ratios. To ensure the comparability between the different time points and to enable a combination of the dynamically weighted edges, each pBI* score value is normalized by the maximum score of each measurement time, before it is assigned as weight to the corresponding edge. The resulting weights take on values in the range of [0,1] and thus are comparable between the graphs at different measurement points. Similar to the procedure using binary weights, continuous weights are entered in the respective adjacency matrix and summed over time. Thus, if n networks are combined, each matrix entry is a continuous value y ∈ [0; n] which are rescaled to achieve edge weights being independent from the number of combined graphs. Afterwards, the rescaled values are assigned as edge weights to the combined, superimposed graph. Analogous to the discrete weighting approach, the edge weights are displayed as lines of different thickness, again serving as an indicator for their frequency of occurrence weighted by the accumulated pBI* score in the superimposed network graphs. The R-based computational framework is available in S1 File.

#### Verification of inferred dynamic networks

The networks constructed provide information on the degree of connection and strength of interaction between the preselected metabolites. To verify these findings based on the given graph representation, a KEGG database query combined with a literature search was conducted for pathway identification, mapping and verification of the underlying biochemical and molecular mechanisms (see also Table 1) [27].

**Table 1:** Results of a representative KEGG database and literature search.

Metabolic pathway		Related metabolites		Related disease	
KEGG ID	Pathway	KEGG ID	KEGG Compound	KEGG ID	KEGG Disease / Disease-Related References
map00230	Purine metabolism	C00385	Xanthine	H00674	Anemia due to disorders of nucleotide metabolism
		C00262	Hypoxanthine	H00824	Calcification of joints and arteries
map00240	Pyrimidine metabolism	C00112	CDP (cytidine diphosphate)	H00674	Anemia due to disorders of nucleotide metabolism
		C00063	CTP (cytidine triphosphate)	H00824	Calcification of joints and arteries
map04152 0	AMPK signaling pathway	C00020	AMP (adenosine monophosphate)	-	Regulation of cell metabolism in cardiometabolic diseases (atherosclerosis, heart failure, diabetes) [29]
map00340	Histidine metabolism*	C00386	Carnosine	-	Cholesterol and glucose metabolism (diabetic nephropathy) [30, 31]

The first main column (with two sub-columns) represents metabolic pathways, the second main column shows metabolites important for the respective pathway and the third main column depicts disease entries linked to the respective metabolic pathway.

* interacting with the purine metabolism.

## Results

The proposed computational approach for kinetic network representation using time-series data combines (i) feature selection to preselect a subset of metabolites with enhanced predictive value using pBI scoring, and subsequently (ii) construction of the dynamic network by translating the kinetic information of circulating metabolites into a superimposed graph representation. For the latter step, a threshold value needs to be defined to specify the degree of interconnectability of analyte pairs present in the network (see section Combined network plots and edge weighting).

Fig 1 shows the absolute pBI* scores of the given dataset in boxplot representation used for network inference at the different time points.

Specifically, the upper limit of each single box indicates the dynamic cutoff τ_i,2_ deduced from the quartile q75, resulting in different threshold values for each measurement point t_i_. The horizontal lines represent the static threshold τ_s_ (s = 1 …3 representing q50, q75 and q90), computed as the mean of the dynamic cutoff values τ_i,j_ from the four times t_i_, which is applied to all measurement points uniformly to generate the superimposed dynamic graph representation. For example, the selected threshold τ_s_ = τ_2_ = 55.36 (q75, yellow line) prioritizes metabolites and their interactions accordingly into the classes of moderate and strong predictors, characterized by their absolute pBI* scores and weights of network edges in the combined graph.

**Fig 2:**
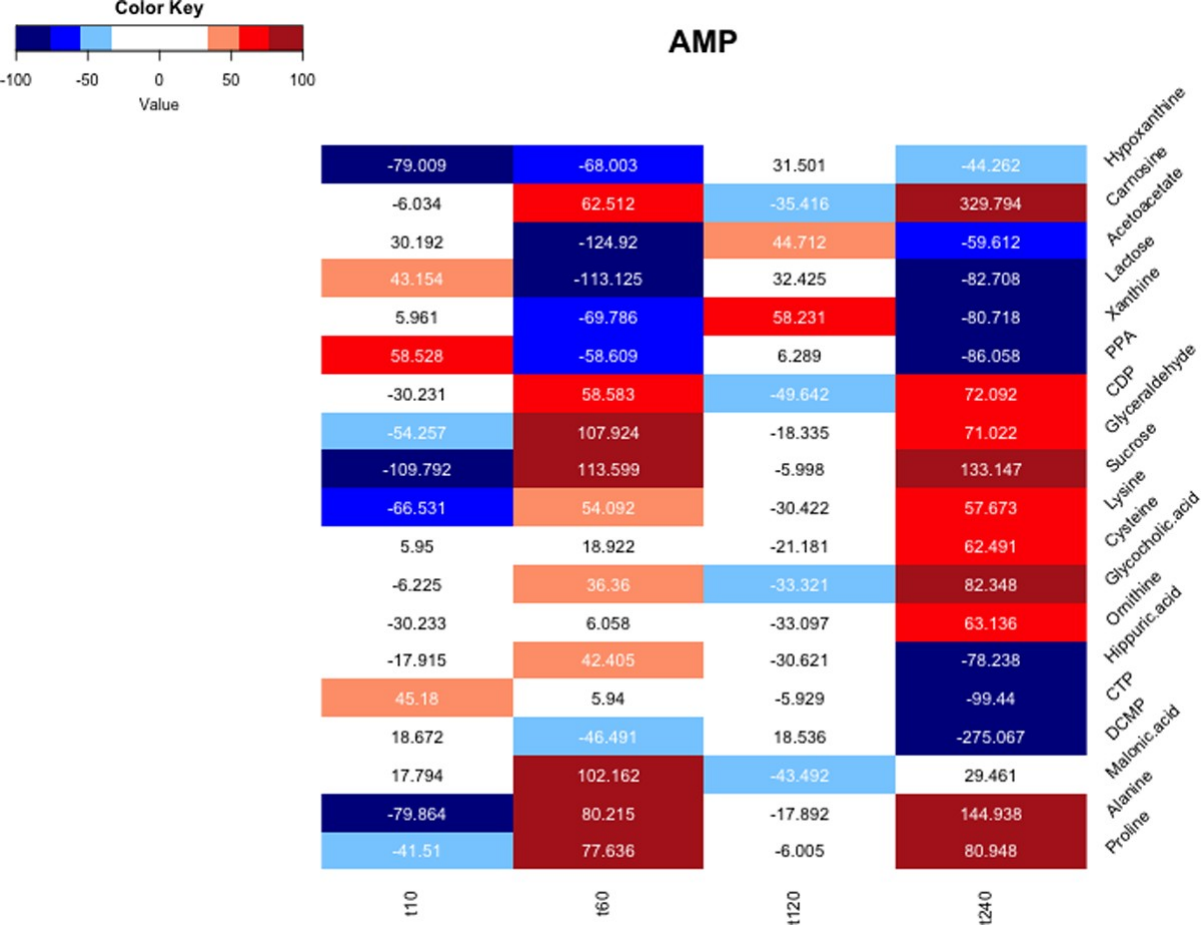
Heatmap representing metabolite interactions originating from the metabolite AMP. The color code indicates the connection strength between AMP and the respective selected metabolites. A high absolute pBI*score indicates a strong interaction between two metabolites and is represented by darker colors (dark red and dark blue).

Fig 2 shows the heatmap of a subset of the 20 top ranked, annotated metabolites of a combined network graph based on static thresholding τ_s_ = τ_3_ (q90), in which connections originate from the representative network metabolite AMP and their respective pBI* scores for each of the four time points are computed.

Numbers in the map denote the computed absolute pBI* scores of metabolite level ratios between AMP and the respective selected metabolites. The color code thus indicates the interaction strength that is directly associated with the absolute value of the pBI* score. Using this color key, analytes are categorized with regard to their predictive value and strength of their respective connections. For instance, AMP has the strongest relationship with the listed metabolites at t_240_ and the weakest relationship with the listed metabolites at t_120_. These patterns indicate the dynamics of interaction among metabolites at different time points, demonstrating an enhanced regulatory activity for AMP (AMP is involved in the regulation of cell metabolism in multiple cardiometabolic diseases) one hour and four hours after myocardial injury.

**Fig 3:**
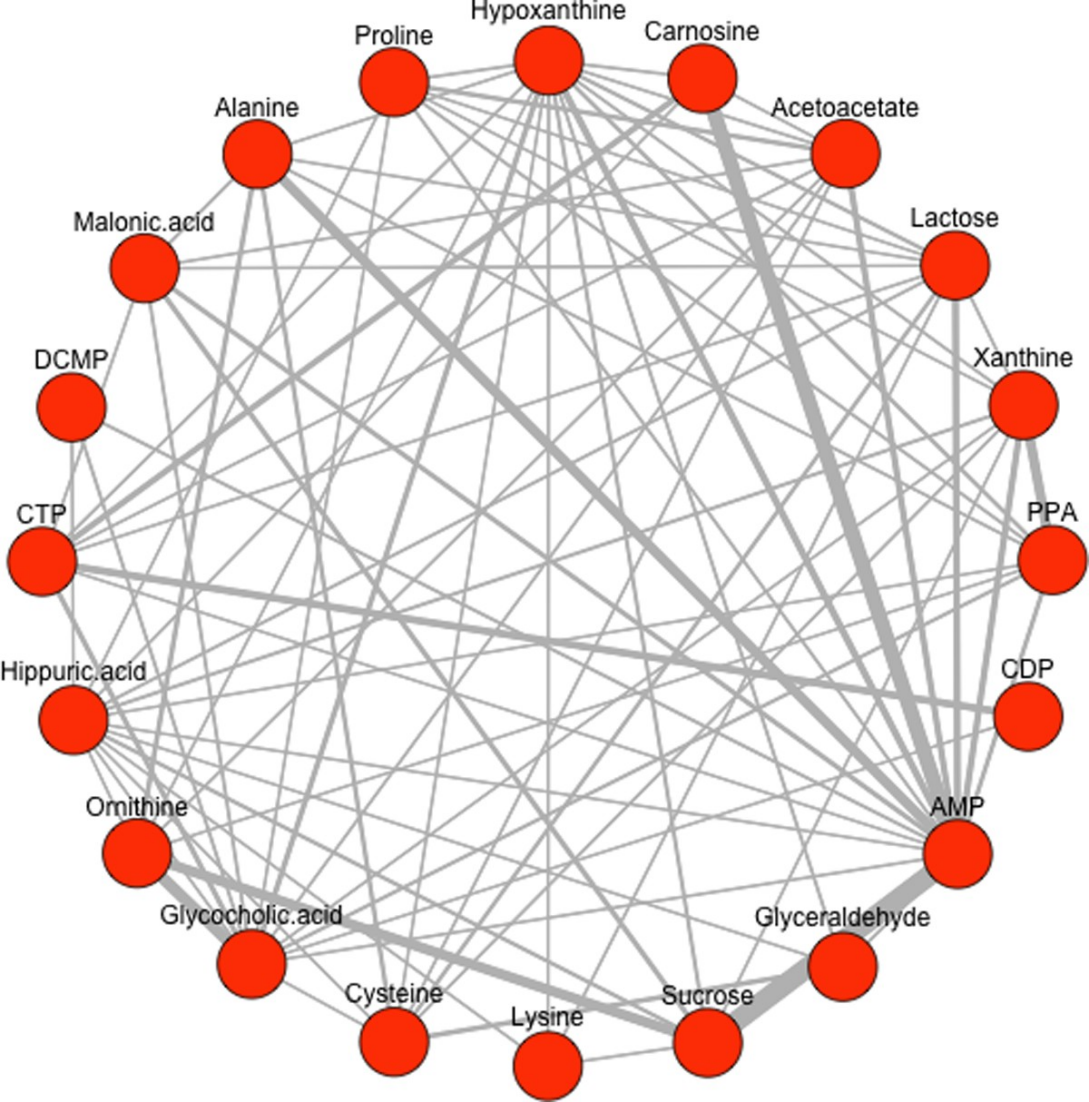
Combined network graph with continuous weighted edges. The graph combines the networks of the four different measurement points inferred with a static threshold τ_s_ = τ_3_ (q90). The pBI* score values of all time points are considered to weight the edges using the *continuous weighting* approach. Thicker line widths indicate stronger or strongest interactions between two metabolites (e.g. AMP ⟷ carnosine or alanine at t_240_ or AMP ⟷ sucrose at multiple time points t_10_, t_60_ and t_240_; see heatmap Fig 2).

Fig 3 illustrates the combined, superimposed dynamic network graph after the pBI score-based selection of a subset of 20 analytes using the fully annotated dataset (see S3 Dataset). The depicted kinetic network graph is inferred using the *continuous weighting* approach for highlighting the various strengths of connections, i.e. thickness of edges between the network metabolites by combining the network edge information of the four time points. This means that the pBI* scores of all single networks are considered. Thicker lines indicate higher pBI* scores, showing stronger interactions between analytes, considering one or multiple measurement points. For visualization a circular graph representation was selected.

**Fig 4:**
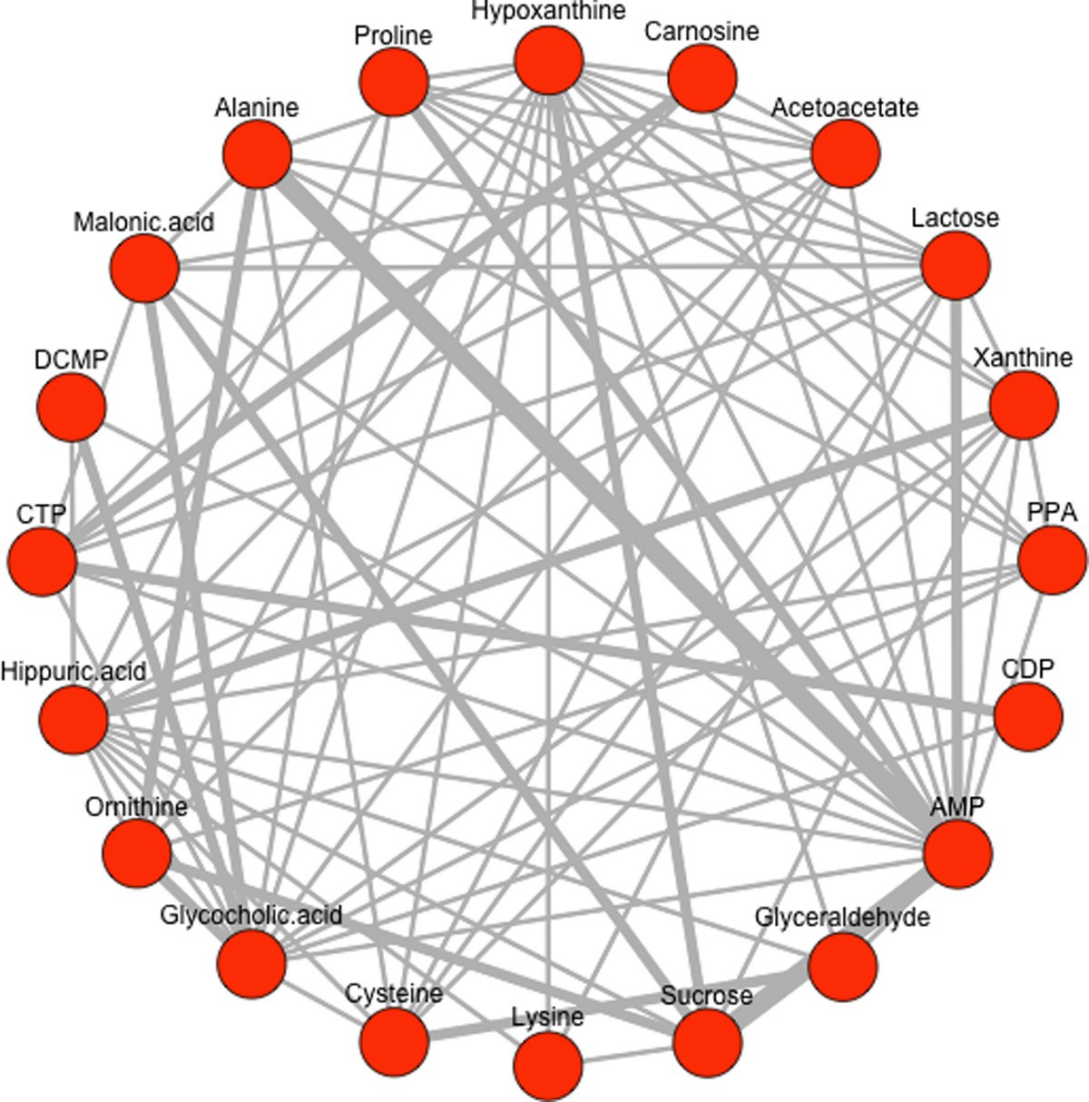
Combined network graph with discrete weighted edges. The graph combines the networks of the four measurement points inferred with the static threshold τ_s_ = τ_3_ (q90) where thicker line widths indicate stronger interactions and serves as an indicator for the frequency of its occurrence in all of the networks combined.

The graph combines the networks of the four different measurement points inferred with a static threshold τ_s_ = τ_3_ (q90), considering all time points to weight the edges using the continuous weighting approach. Fig 4 illustrates a second variant of a superimposed network graph again inferred with the static threshold τ_s_ = τ_3_ (q90), combining networks from the same four measurement points as demonstrated in Fig 3. In contrast, the edges in the resulting combined graph are highlighted using the *discrete weighting* approach. Thicker line widths indicate stronger interactions between the respective metabolites in terms of their occurrence in the network. Note that for both networks (Figs 3 and 4) we used the highest static threshold at q90 (= 90% percentile) to visualize only the strongest interactions among the selected metabolites. All networks inferred with the static thresholds τ_s_ = q50, q75 and q90 are available in S1 File, Example Plots.

**Fig 5:**
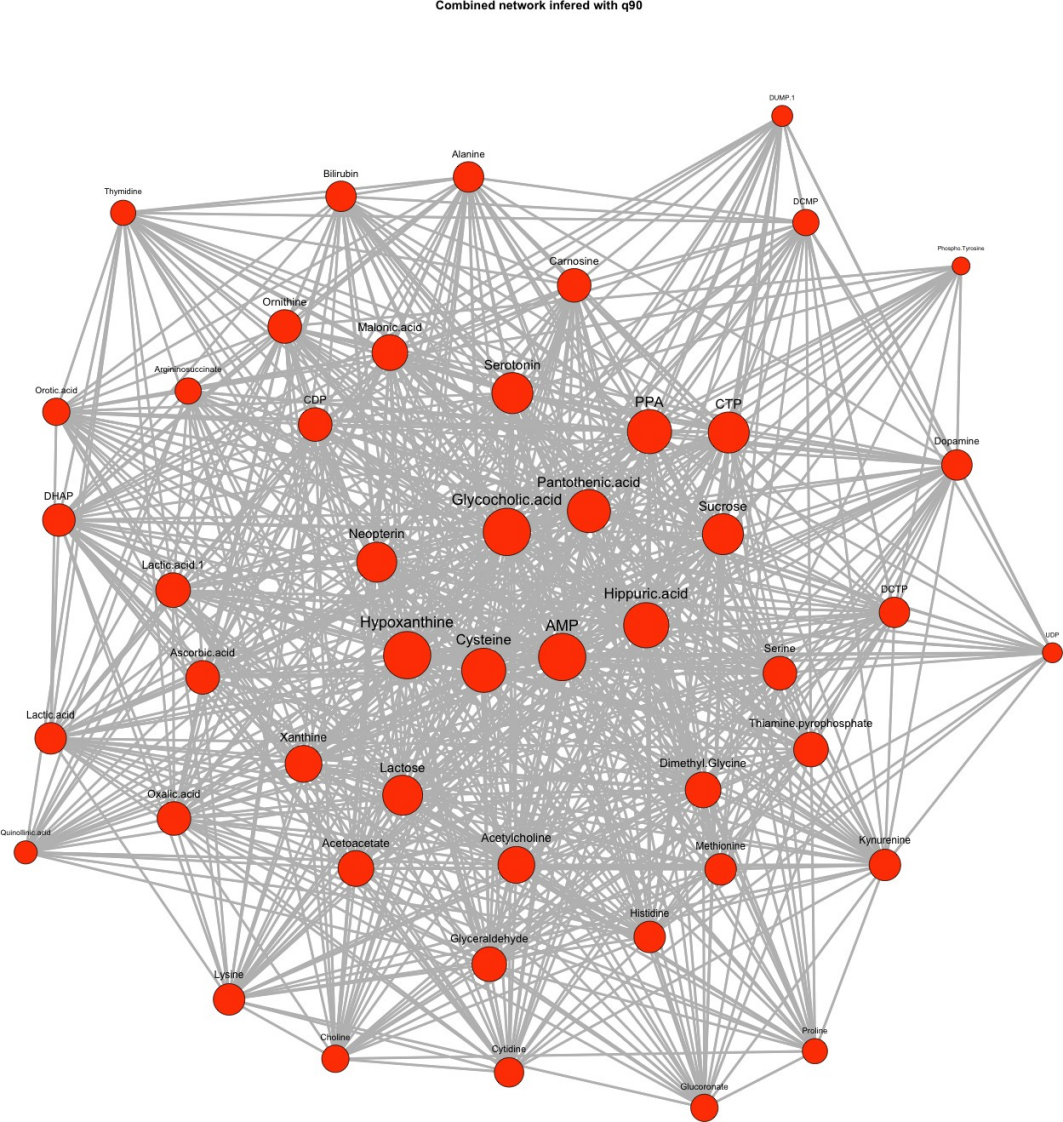
Combined network graph of all measurement points with degree-based edge weighting. The graph combines the single networks of the four time points t_i_ inferred with a dynamic threshold τ_i,3_ (= q90 for i = 1….4). The node size is adapted to the degree of the respective edges with larger node sizes indicating higher node degrees. In total, a number of n = 48 preselected analytes are displayed in the network graph.

In addition to the two presented edge-weighting concepts, an alternative method of inferring the network is briefly introduced which uses the concept of weighting the nodes instead of the edges. Using this concept, the combined network graph is created upon a *degree-based (discrete) node weighting* modality as depicted in Fig 5.

The superimposed graph combines the single networks inferred at the four different time points t_i_ using the individual dynamic thresholds τ_i,3_ (= q90 for i = 1….4). For metabolite subset selection a pBI score threshold at q75 was selected, leading to a higher number of preselected analytes (n = 48) as demonstrated in Fig 5. Here, the node size in the graph depends on the respective node degree, i.e. the number of connections corresponding to a node by combining again the individual network graphs of all measurement points into one superimposed graph representation. For visualization the force-directed Fruchterman-Reingold layout was selected [28]. For biochemical interpretation and verification, a KEGG database and literature search was conducted. Relevant pathways and metabolic interactions associated with cardiometabolic diseases, in particular with myocardial injury, are summarized in Table 1.

As a proof-of-concept, significant metabolite interactions highlighted in the dynamic graphs (see Figs 3, 4 and 5) include the metabolites xanthine and hypoxanthine (prioritized as moderate/strong predictors), both associated with purine metabolism, as well as CDP and CTP, which are related to pyrimidine metabolism. Multiple network connections originating from adenosine monophosphate (AMP), linked to the AMPK signaling pathway, are highlighted as well. AMPK is a key energy sensor that regulates metabolism at the level of both the cell and the whole organism. It is activated during metabolic stress, under conditions where the generation of ATP decreases or ATP consumption increases, and leads to the inhibition of anabolic pathways. Therefore, it can be considered to be associated with cellular stress that occur after a myocardial infarction. Reviewing the AMPK signaling pathway in more detail, an interaction with the starch and sucrose metabolism can also be shown [32, 33].

A strong network connection between alanine and AMP was also present, potentially representing alanine-mediated activation of AMPK [34].

Interestingly, a new and so far unexpected analyte, glycocholic acid, and its relationship with ornithine are prominently represented in the network graphs. This metabolite requires further consideration, as it has been discussed as a potential biomarker of liver injury in rodent toxicity studies, but its role in myocardial injury has not been characterized [35]. However, other bile acids have recently been implicated in the dysregulation of cardiac metabolism and the development of cardiomyopathy, highlighting the biological plausibility of glycocholic acid as a biomarker of cardiovascular disease [36].

## Discussion

The presented computational approach for the identification, prioritization and characterization of interactions of new dynamic metabolic biomarker candidates gathered from longitudinal MS high throughput data provides a powerful tool for biomarker discovery by translating the kinetics of the data into a combined graph representation. Our proposed approach can be divided into three steps: (i) data preprocessing, (ii) feature subset selection and (iii) network construction. The latter two steps are based on the pBI score measure introduced by Baumgartner et al. [24] for selecting and prioritizing metabolite data. In particular, the pBI score is used as a decision criterion for preselecting candidate metabolites upon their discriminatory ability from time series data. In step three, the kinetic network based on different edge weighting approaches is inferred. Here, the introduced pBI* score is combined with the threshold τ to identify significant edges out of all possible connections in a network. In principle, these cut-off values are computed dynamically according to a statistical model based on interquartile ranges so that the resulting networks created from multiple time points yield the same number of edges. In this regard, static thresholding was used to infer combined, superimposed networks as demonstrated in Figs 3 and 4, whilst Fig 5 demonstrates a combined network graph by using dynamic thresholds alternatively. However, it is important to note that the task of choosing the threshold τ for network edge selection is crucial for setting up networks for the subsequent biological interpretation of metabolite interactions.

Specifically, the proposed method for inferring kinetic network graphs with various edge weighting concepts (discrete vs. continuous) represents a powerful approach for discovering metabolic signatures in terms of their discriminatory ability and their strength of interactions over time, as demonstrated in the selected clinical example of myocardial injury. Two different strategies for weighting the network edges were introduced. First, the discrete approach, which uses the adjacency matrices of the graphs to be combined, presents a straightforward method where all edges occurring in the underlying network graphs are considered equally since the weights take on discrete values according to their occurrence frequency in the networks to combine. Second, the continuous approach used for calculating the edge weights takes into account the respective pBI* score values of all edges in the network graphs. This results in continuous weights which are related to the strengths of connections over all time points and therefore allows for a more differentiated quantification of the underlying analyte interactions. When comparing the different edge weighting approaches, more prominent edges are highlighted with thick lines for the discrete calculation of edges than for those of a continuous type. This may lead to misinterpretations, especially if low threshold values for network inference are chosen. Therefore, it is recommended to combine both edge weighting approaches for the biological interpretation of findings, starting with “discrete” weighting for preselection of all promising interactions, and subsequently differentiation of the impact of interactions by the “continuous” weighting method.

As a result of this applied new computational approach, the presented networks highlight a broad panel of promising key metabolites and the chemical interaction of analyte pairs in the network, which were evaluated with an appropriate pathway database, i.e. the KEGG database, as an essential initial step for the verification and interpretation of findings (see Table 1). This analysis enabled the confirmation of known metabolic signatures—in addition to some unexpected candidates such as carnosine or glycocholic acid—and pathways that have been previously associated with cardiovascular disease or related disorders [15, 29–36].

## Conclusions

Using the proposed bioinformatics approach of inferring dynamic networks for biomarker discovery in complex diseases like myocardial infarction, we identified promising single putative biomarker candidates and provided additional quantitative information on the analytes’ interconnectability in single or multiple metabolic pathways over time. In particular, the introduction of dynamic networks provides a further source of information, representing not only the kinetics of the metabolite changes themselves, but also their chemical interactions over time.

Furthermore, our results have demonstrated biochemical and biological plausibility, indicating that this approach can serve as a significant tool for aiding in the discovery of dynamic metabolic or proteomic biomarkers in clinical cardiology.

## Supporting information

S1 Dataset Original dataset. This is the original, unprocessed dataset with n = 210 analytes, including missing values, but already cleaned for extreme outliers (file name in R-scripts: DataAll).
(CSV)

S2 Dataset Preprocessed dataset. This is the preprocessed and cleaned dataset (n = 170 analytes) after handling missing values by replacement with the median of analyte levels at the given time point (file name in R-scripts: PreprocessedAll).
(CSV)

S3 Dataset Annotated dataset. This is the fully identified and annotated dataset of n = 71 metabolites for final network construction (file name in R-scripts: DataPreprocessedAnnotated).
Measurement values in all three datasets (S1–S3 Datasets) represent metabolite levels in MS intensity units (IU) for the five defined measurements before alcohol septal ablation (baseline at time t_0_) and t_10_ = 10 min, t_60_ = 60 min, t_120_ = 120 min and t_240_ = 240 min after myocardial injury.
S1–S3 Datasets are available in CVS format. The first column represents a class level (1…time point t_0_, 2…time point t_10_, etc.), the second column indicates the explicit time point for a selected case such as D_t0_p1 (time point t_10_ for case p1), the third column shows again the case ID and the rest of columns comprises the analyte levels denoted in MS intensity units (IU) for each metabolite at the different time points.
(CSV)

S1 File Computational framework. The R-based computational framework for data preprocessing, metabolite subset selection and dynamic network construction consists of the following R-scripts and text files:
1. Main.R: Main script for the analysis of metabolic data in order to identify putative biomarker candidates based on dynamic network visualization.
2. Preprocessing.R: (i) Removes metabolites with more than 60% of the values missing from the dataset; (ii) Replaces missing values with the metabolite's median at a given time point for the remaining dataset; (iii) Creates a data subset, containing only those metabolites, which are present at all time points.
3. BI.R: Function that calculates pBI scores for all metabolites.
4. InferBIGraph.R: Function to sum up the function calls for the calculation of a network graph.
5. FunctionsGraph.R: Multiple functions for network graph construction and visualization (i.e. create boxplot diagrams with different thresholds, graph calculation, adapt ratio for heatmap construction, plot heatmaps, plot graphs, calculate discrete weights, calculate degree-based weights, calculate graph object, plot pBI scores as bar charts).
6. *.txt files: Contain coordinates for graph visualization.
For further information please read the “ReadMe.txt” file in the Supporting information.
(ZIP)
