# Supplementary figures and images for: A novel network-based approach for discovering dynamic metabolic biomarkers in cardiovascular disease
Source: PLoS One. 2018 Dec 11;13(12):e0208953. doi: 10.1371/journal.pone.0208953 (PMC6289413; doi:10.1371/journal.pone.0208953)

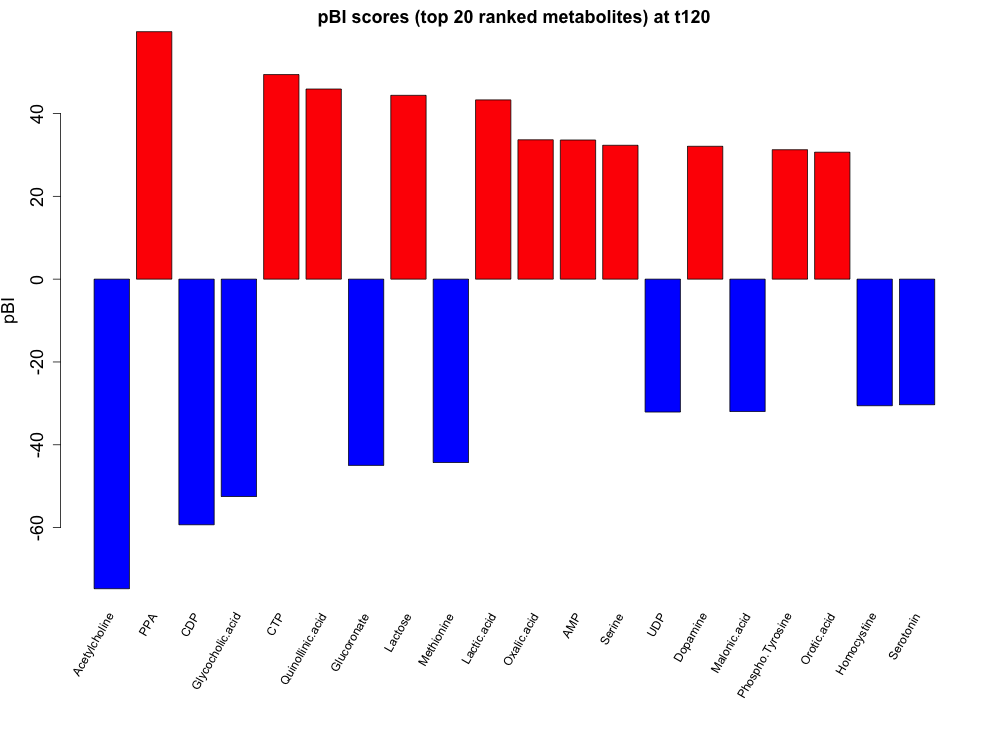

Supplement: S1 File — The R-based computational framework for data preprocessing, metabolite subset selection and dynamic network construction consists of the following R-scripts and text files: 1. Main.R: Main script for the analysis of metabolic data in order to identify putative biomarker candidates based on dynamic network visualization. 2. Preprocessing.R: (i) Removes metabolites with more than 60% of the values missing from the dataset; (ii) Replaces missing values with the metabolite's median at a given time point for the remaining dataset; (iii) Creates a data subset, containing only those metabolites, which are present at all time points. 3. BI.R: Function that calculates pBI scores for all metabolites. 4. InferBIGraph.R: Function to sum up the function calls for the calculation of a network graph. 5. FunctionsGraph.R: Multiple functions for network graph construction and visualization (i.e. create boxplot diagrams with different thresholds, graph calculation, adapt ratio for heatmap construction, plot heatmaps, plot graphs, calculate discrete weights, calculate degree-based weights, calculate graph object, plot pBI scores as bar charts). 6. *.txt files: Contain coordinates for graph visualization. For further information please read the “ReadMe.txt” file in the Supporting information. (ZIP) [file pone.0208953.s004.zip › S1_File/output/Example_Plots/01_pBI/barplot_pBI_t120.png]

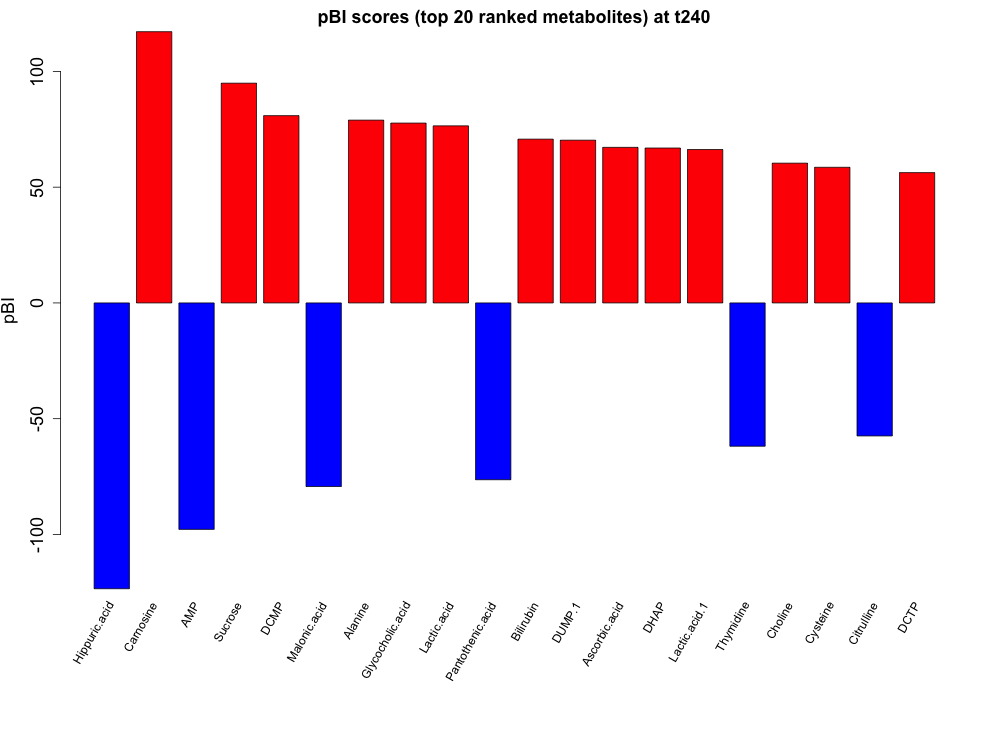

Supplement: S1 File — The R-based computational framework for data preprocessing, metabolite subset selection and dynamic network construction consists of the following R-scripts and text files: 1. Main.R: Main script for the analysis of metabolic data in order to identify putative biomarker candidates based on dynamic network visualization. 2. Preprocessing.R: (i) Removes metabolites with more than 60% of the values missing from the dataset; (ii) Replaces missing values with the metabolite's median at a given time point for the remaining dataset; (iii) Creates a data subset, containing only those metabolites, which are present at all time points. 3. BI.R: Function that calculates pBI scores for all metabolites. 4. InferBIGraph.R: Function to sum up the function calls for the calculation of a network graph. 5. FunctionsGraph.R: Multiple functions for network graph construction and visualization (i.e. create boxplot diagrams with different thresholds, graph calculation, adapt ratio for heatmap construction, plot heatmaps, plot graphs, calculate discrete weights, calculate degree-based weights, calculate graph object, plot pBI scores as bar charts). 6. *.txt files: Contain coordinates for graph visualization. For further information please read the “ReadMe.txt” file in the Supporting information. (ZIP) [file pone.0208953.s004.zip › S1_File/output/Example_Plots/01_pBI/barplot_pBI_t240.png]

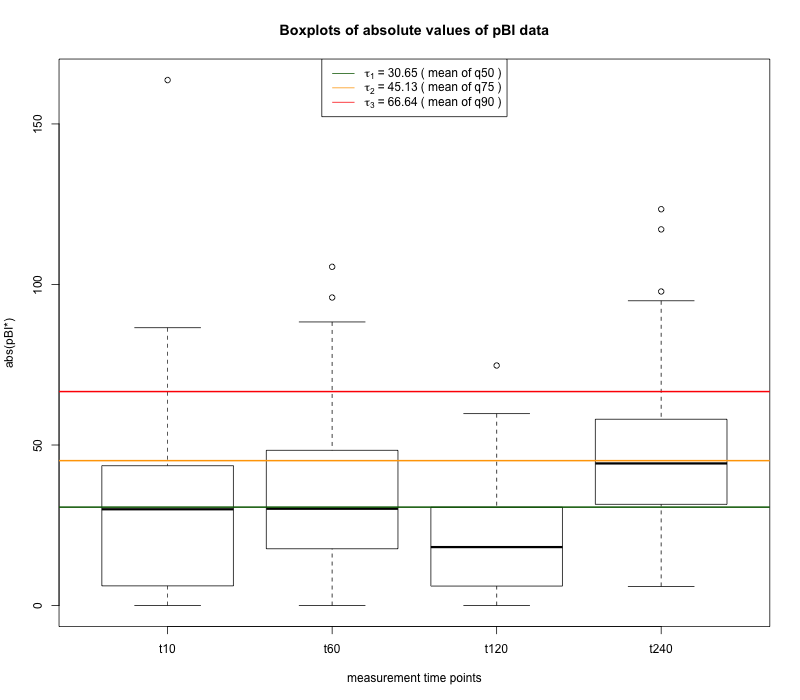

Supplement: S1 File — The R-based computational framework for data preprocessing, metabolite subset selection and dynamic network construction consists of the following R-scripts and text files: 1. Main.R: Main script for the analysis of metabolic data in order to identify putative biomarker candidates based on dynamic network visualization. 2. Preprocessing.R: (i) Removes metabolites with more than 60% of the values missing from the dataset; (ii) Replaces missing values with the metabolite's median at a given time point for the remaining dataset; (iii) Creates a data subset, containing only those metabolites, which are present at all time points. 3. BI.R: Function that calculates pBI scores for all metabolites. 4. InferBIGraph.R: Function to sum up the function calls for the calculation of a network graph. 5. FunctionsGraph.R: Multiple functions for network graph construction and visualization (i.e. create boxplot diagrams with different thresholds, graph calculation, adapt ratio for heatmap construction, plot heatmaps, plot graphs, calculate discrete weights, calculate degree-based weights, calculate graph object, plot pBI scores as bar charts). 6. *.txt files: Contain coordinates for graph visualization. For further information please read the “ReadMe.txt” file in the Supporting information. (ZIP) [file pone.0208953.s004.zip › S1_File/output/Example_Plots/01_pBI/abs_pBI_thresholds.png]

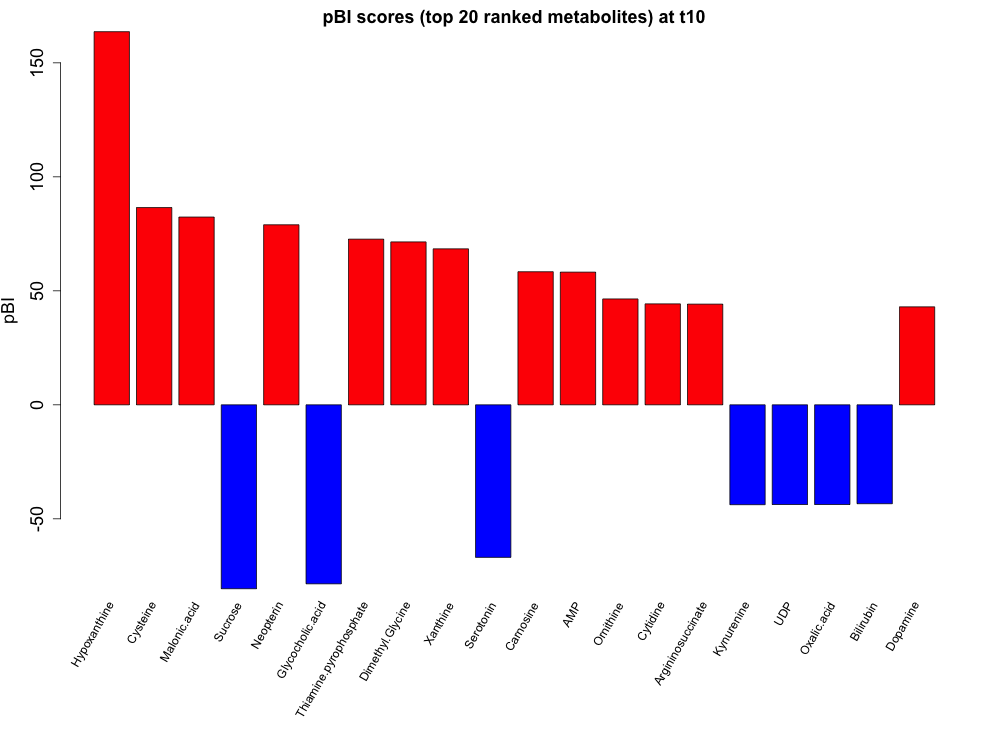

Supplement: S1 File — The R-based computational framework for data preprocessing, metabolite subset selection and dynamic network construction consists of the following R-scripts and text files: 1. Main.R: Main script for the analysis of metabolic data in order to identify putative biomarker candidates based on dynamic network visualization. 2. Preprocessing.R: (i) Removes metabolites with more than 60% of the values missing from the dataset; (ii) Replaces missing values with the metabolite's median at a given time point for the remaining dataset; (iii) Creates a data subset, containing only those metabolites, which are present at all time points. 3. BI.R: Function that calculates pBI scores for all metabolites. 4. InferBIGraph.R: Function to sum up the function calls for the calculation of a network graph. 5. FunctionsGraph.R: Multiple functions for network graph construction and visualization (i.e. create boxplot diagrams with different thresholds, graph calculation, adapt ratio for heatmap construction, plot heatmaps, plot graphs, calculate discrete weights, calculate degree-based weights, calculate graph object, plot pBI scores as bar charts). 6. *.txt files: Contain coordinates for graph visualization. For further information please read the “ReadMe.txt” file in the Supporting information. (ZIP) [file pone.0208953.s004.zip › S1_File/output/Example_Plots/01_pBI/barplot_pBI_t10.png]

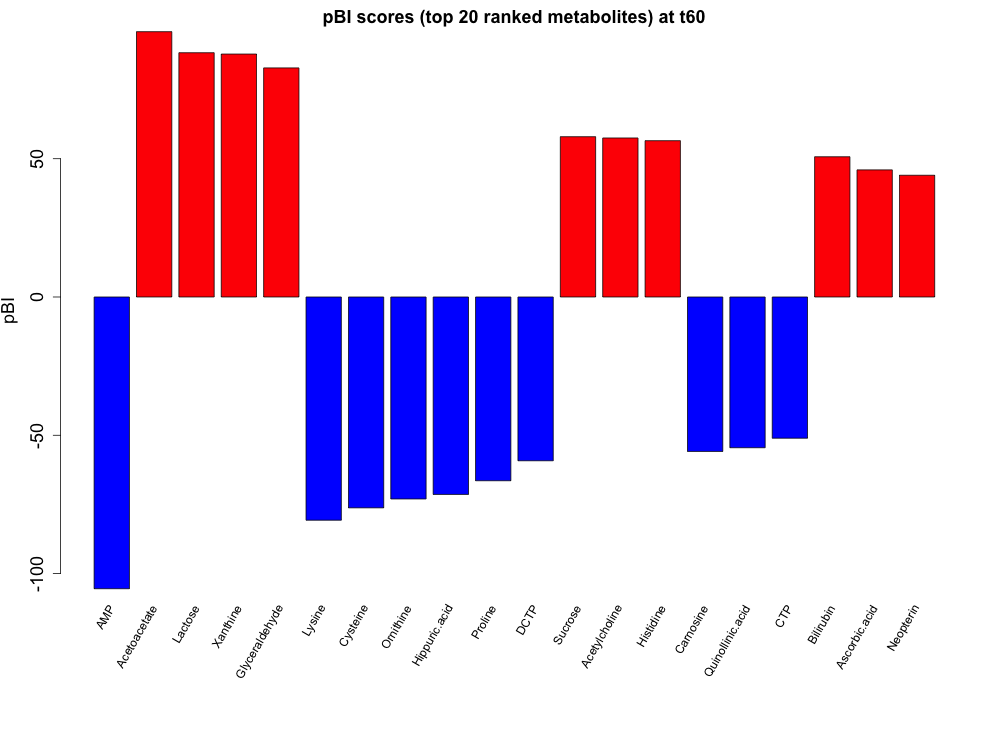

Supplement: S1 File — The R-based computational framework for data preprocessing, metabolite subset selection and dynamic network construction consists of the following R-scripts and text files: 1. Main.R: Main script for the analysis of metabolic data in order to identify putative biomarker candidates based on dynamic network visualization. 2. Preprocessing.R: (i) Removes metabolites with more than 60% of the values missing from the dataset; (ii) Replaces missing values with the metabolite's median at a given time point for the remaining dataset; (iii) Creates a data subset, containing only those metabolites, which are present at all time points. 3. BI.R: Function that calculates pBI scores for all metabolites. 4. InferBIGraph.R: Function to sum up the function calls for the calculation of a network graph. 5. FunctionsGraph.R: Multiple functions for network graph construction and visualization (i.e. create boxplot diagrams with different thresholds, graph calculation, adapt ratio for heatmap construction, plot heatmaps, plot graphs, calculate discrete weights, calculate degree-based weights, calculate graph object, plot pBI scores as bar charts). 6. *.txt files: Contain coordinates for graph visualization. For further information please read the “ReadMe.txt” file in the Supporting information. (ZIP) [file pone.0208953.s004.zip › S1_File/output/Example_Plots/01_pBI/barplot_pBI_t60.png]

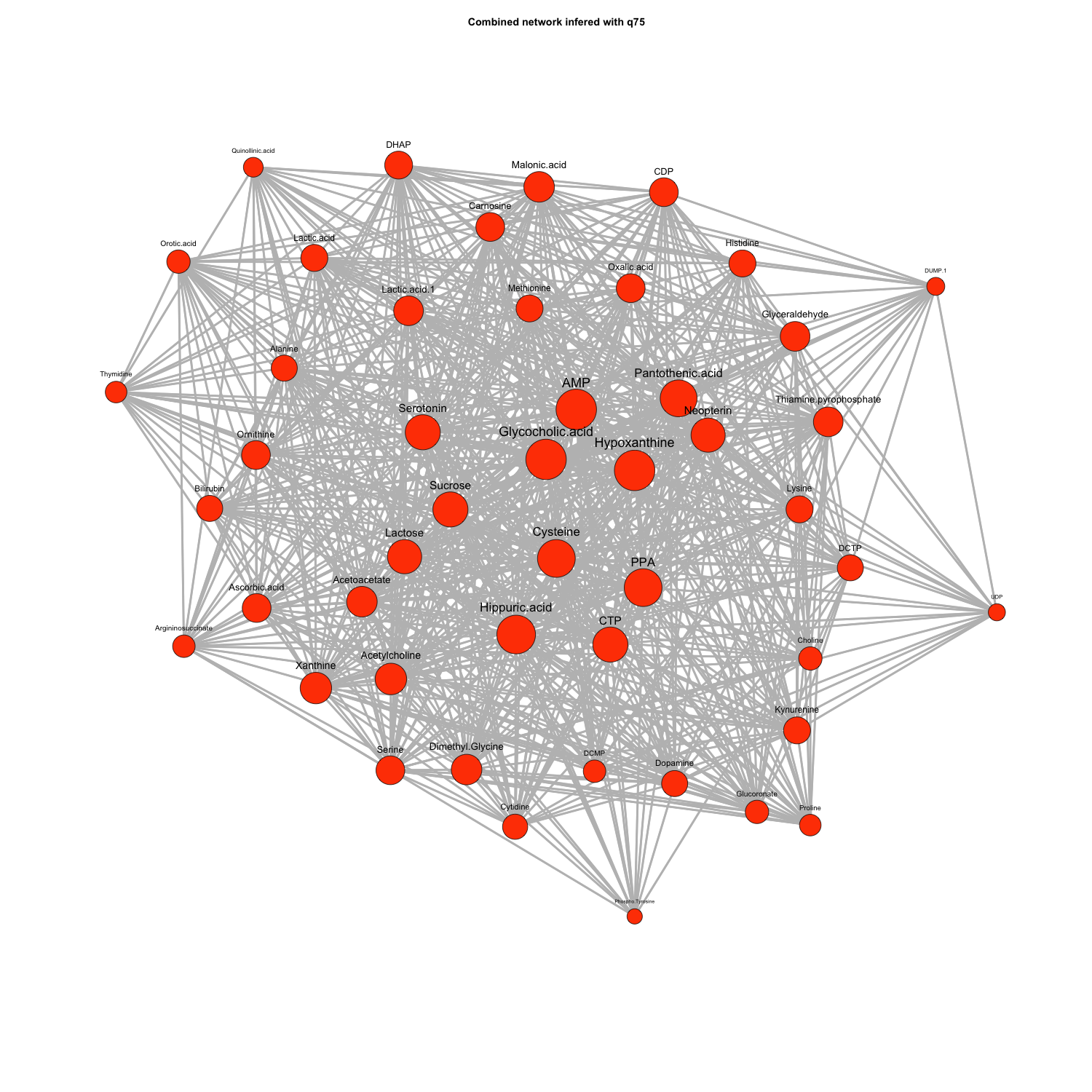

Supplement: S1 File — The R-based computational framework for data preprocessing, metabolite subset selection and dynamic network construction consists of the following R-scripts and text files: 1. Main.R: Main script for the analysis of metabolic data in order to identify putative biomarker candidates based on dynamic network visualization. 2. Preprocessing.R: (i) Removes metabolites with more than 60% of the values missing from the dataset; (ii) Replaces missing values with the metabolite's median at a given time point for the remaining dataset; (iii) Creates a data subset, containing only those metabolites, which are present at all time points. 3. BI.R: Function that calculates pBI scores for all metabolites. 4. InferBIGraph.R: Function to sum up the function calls for the calculation of a network graph. 5. FunctionsGraph.R: Multiple functions for network graph construction and visualization (i.e. create boxplot diagrams with different thresholds, graph calculation, adapt ratio for heatmap construction, plot heatmaps, plot graphs, calculate discrete weights, calculate degree-based weights, calculate graph object, plot pBI scores as bar charts). 6. *.txt files: Contain coordinates for graph visualization. For further information please read the “ReadMe.txt” file in the Supporting information. (ZIP) [file pone.0208953.s004.zip › S1_File/output/Example_Plots/02_Graphs/Overview_Combined_q75.png]

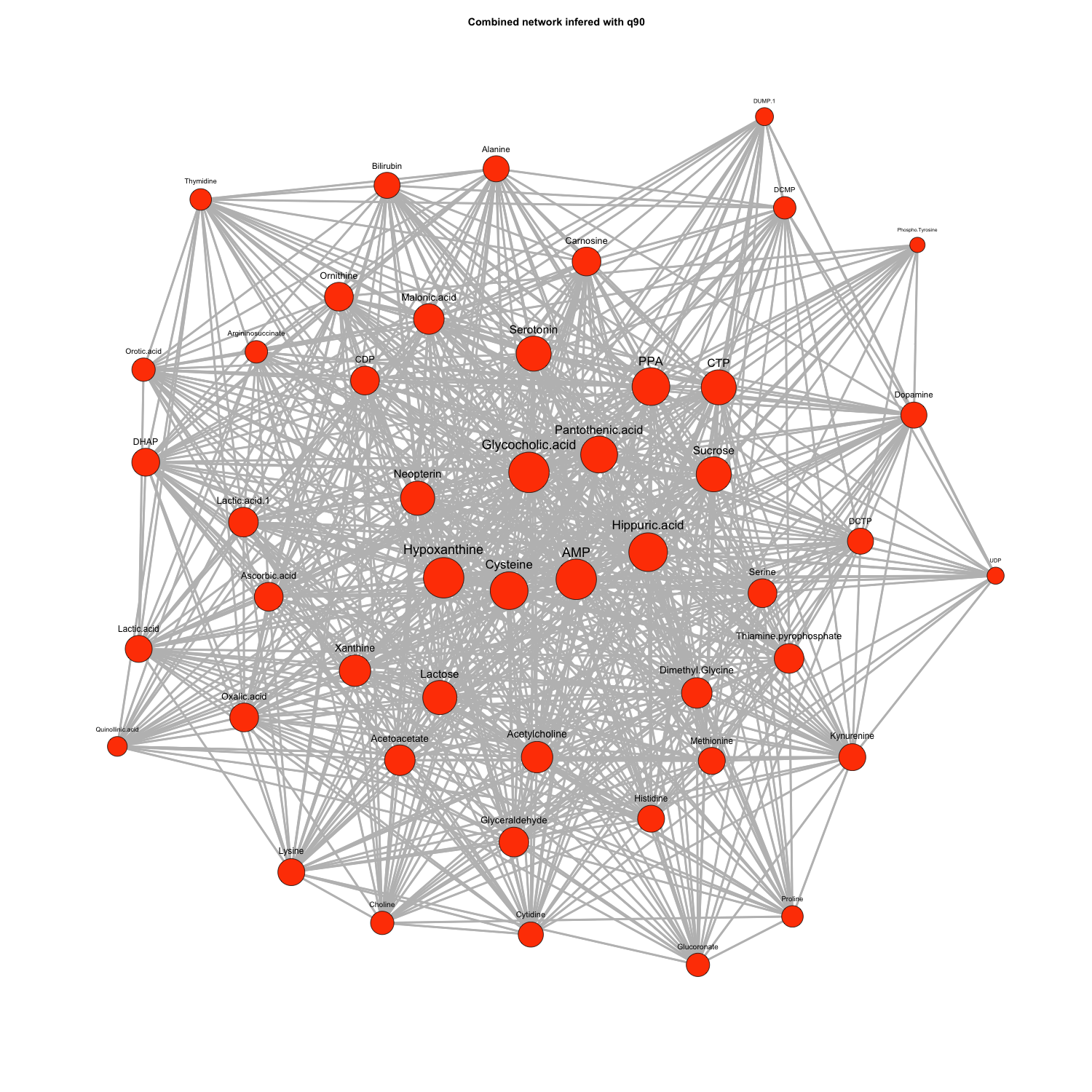

Supplement: S1 File — The R-based computational framework for data preprocessing, metabolite subset selection and dynamic network construction consists of the following R-scripts and text files: 1. Main.R: Main script for the analysis of metabolic data in order to identify putative biomarker candidates based on dynamic network visualization. 2. Preprocessing.R: (i) Removes metabolites with more than 60% of the values missing from the dataset; (ii) Replaces missing values with the metabolite's median at a given time point for the remaining dataset; (iii) Creates a data subset, containing only those metabolites, which are present at all time points. 3. BI.R: Function that calculates pBI scores for all metabolites. 4. InferBIGraph.R: Function to sum up the function calls for the calculation of a network graph. 5. FunctionsGraph.R: Multiple functions for network graph construction and visualization (i.e. create boxplot diagrams with different thresholds, graph calculation, adapt ratio for heatmap construction, plot heatmaps, plot graphs, calculate discrete weights, calculate degree-based weights, calculate graph object, plot pBI scores as bar charts). 6. *.txt files: Contain coordinates for graph visualization. For further information please read the “ReadMe.txt” file in the Supporting information. (ZIP) [file pone.0208953.s004.zip › S1_File/output/Example_Plots/02_Graphs/Overview_Combined_q90.png]

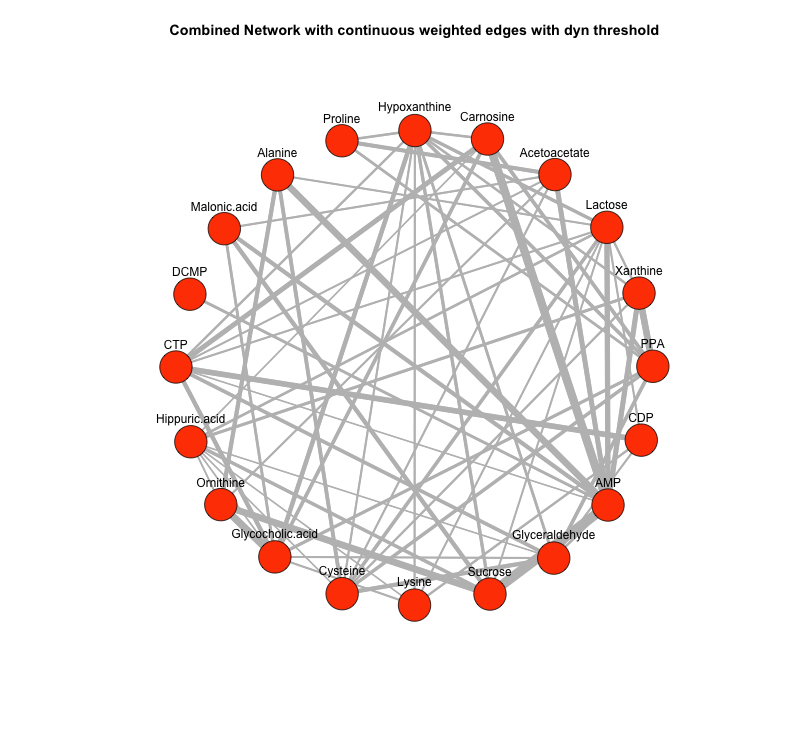

Supplement: S1 File — The R-based computational framework for data preprocessing, metabolite subset selection and dynamic network construction consists of the following R-scripts and text files: 1. Main.R: Main script for the analysis of metabolic data in order to identify putative biomarker candidates based on dynamic network visualization. 2. Preprocessing.R: (i) Removes metabolites with more than 60% of the values missing from the dataset; (ii) Replaces missing values with the metabolite's median at a given time point for the remaining dataset; (iii) Creates a data subset, containing only those metabolites, which are present at all time points. 3. BI.R: Function that calculates pBI scores for all metabolites. 4. InferBIGraph.R: Function to sum up the function calls for the calculation of a network graph. 5. FunctionsGraph.R: Multiple functions for network graph construction and visualization (i.e. create boxplot diagrams with different thresholds, graph calculation, adapt ratio for heatmap construction, plot heatmaps, plot graphs, calculate discrete weights, calculate degree-based weights, calculate graph object, plot pBI scores as bar charts). 6. *.txt files: Contain coordinates for graph visualization. For further information please read the “ReadMe.txt” file in the Supporting information. (ZIP) [file pone.0208953.s004.zip › S1_File/output/Example_Plots/02_Graphs/DynThresh/q90/quad/Network_continuous_weighted_4_graphs_q90dyn.png]

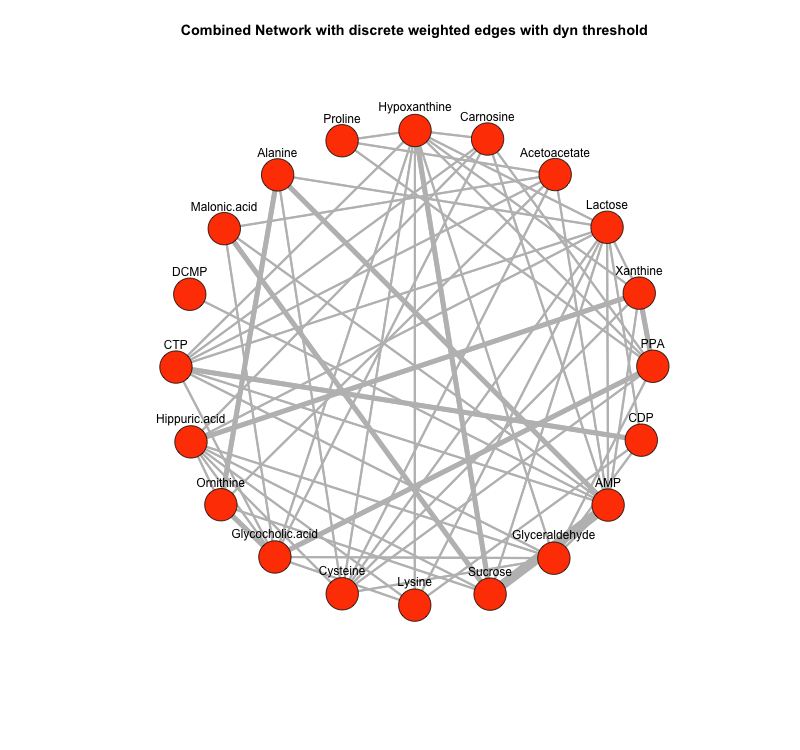

Supplement: S1 File — The R-based computational framework for data preprocessing, metabolite subset selection and dynamic network construction consists of the following R-scripts and text files: 1. Main.R: Main script for the analysis of metabolic data in order to identify putative biomarker candidates based on dynamic network visualization. 2. Preprocessing.R: (i) Removes metabolites with more than 60% of the values missing from the dataset; (ii) Replaces missing values with the metabolite's median at a given time point for the remaining dataset; (iii) Creates a data subset, containing only those metabolites, which are present at all time points. 3. BI.R: Function that calculates pBI scores for all metabolites. 4. InferBIGraph.R: Function to sum up the function calls for the calculation of a network graph. 5. FunctionsGraph.R: Multiple functions for network graph construction and visualization (i.e. create boxplot diagrams with different thresholds, graph calculation, adapt ratio for heatmap construction, plot heatmaps, plot graphs, calculate discrete weights, calculate degree-based weights, calculate graph object, plot pBI scores as bar charts). 6. *.txt files: Contain coordinates for graph visualization. For further information please read the “ReadMe.txt” file in the Supporting information. (ZIP) [file pone.0208953.s004.zip › S1_File/output/Example_Plots/02_Graphs/DynThresh/q90/quad/Network_discrete_weighted_4_graphs_q90dyn.png]

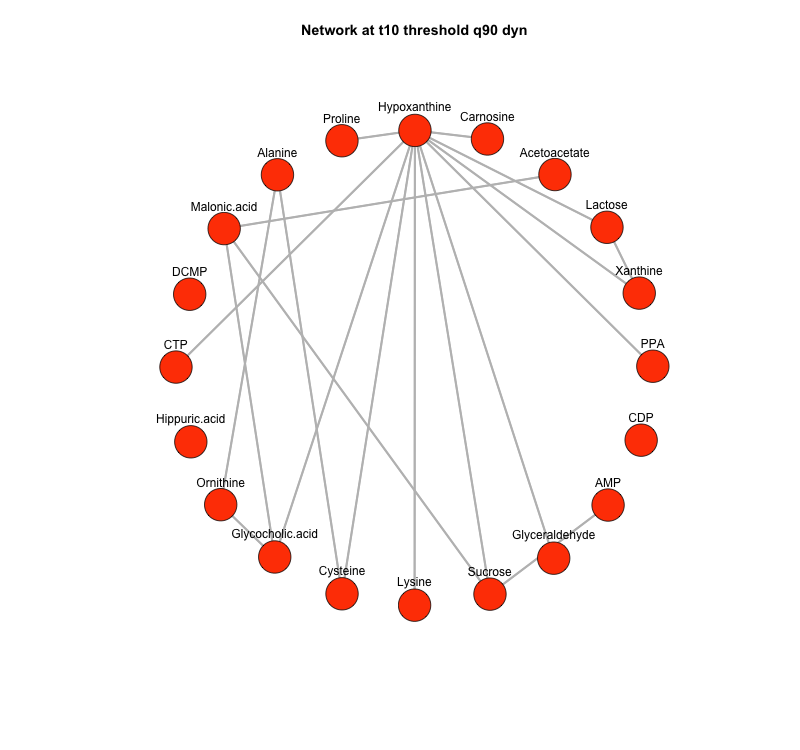

Supplement: S1 File — The R-based computational framework for data preprocessing, metabolite subset selection and dynamic network construction consists of the following R-scripts and text files: 1. Main.R: Main script for the analysis of metabolic data in order to identify putative biomarker candidates based on dynamic network visualization. 2. Preprocessing.R: (i) Removes metabolites with more than 60% of the values missing from the dataset; (ii) Replaces missing values with the metabolite's median at a given time point for the remaining dataset; (iii) Creates a data subset, containing only those metabolites, which are present at all time points. 3. BI.R: Function that calculates pBI scores for all metabolites. 4. InferBIGraph.R: Function to sum up the function calls for the calculation of a network graph. 5. FunctionsGraph.R: Multiple functions for network graph construction and visualization (i.e. create boxplot diagrams with different thresholds, graph calculation, adapt ratio for heatmap construction, plot heatmaps, plot graphs, calculate discrete weights, calculate degree-based weights, calculate graph object, plot pBI scores as bar charts). 6. *.txt files: Contain coordinates for graph visualization. For further information please read the “ReadMe.txt” file in the Supporting information. (ZIP) [file pone.0208953.s004.zip › S1_File/output/Example_Plots/02_Graphs/DynThresh/q90/Network_t10_q90dyn.png]

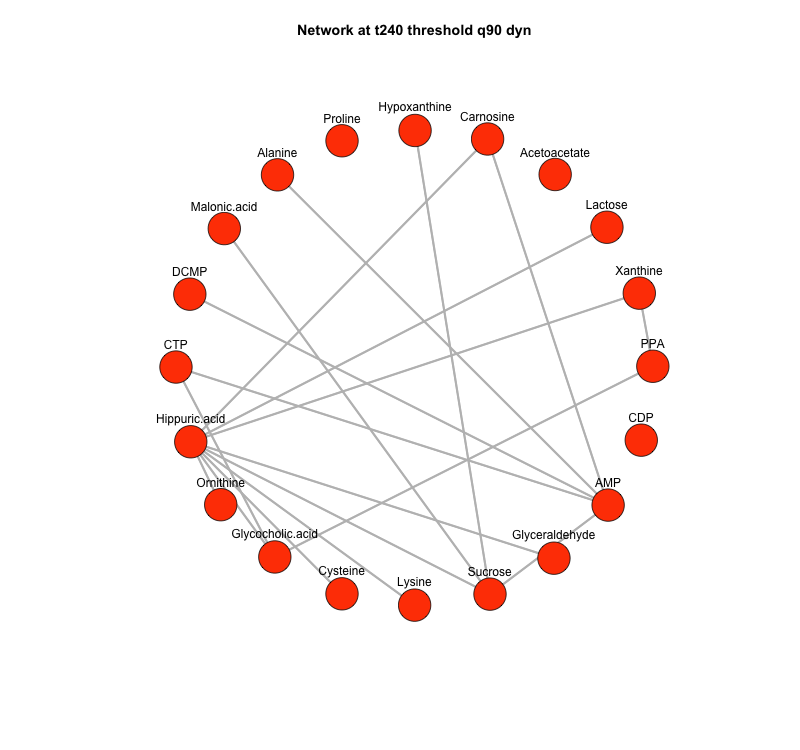

Supplement: S1 File — The R-based computational framework for data preprocessing, metabolite subset selection and dynamic network construction consists of the following R-scripts and text files: 1. Main.R: Main script for the analysis of metabolic data in order to identify putative biomarker candidates based on dynamic network visualization. 2. Preprocessing.R: (i) Removes metabolites with more than 60% of the values missing from the dataset; (ii) Replaces missing values with the metabolite's median at a given time point for the remaining dataset; (iii) Creates a data subset, containing only those metabolites, which are present at all time points. 3. BI.R: Function that calculates pBI scores for all metabolites. 4. InferBIGraph.R: Function to sum up the function calls for the calculation of a network graph. 5. FunctionsGraph.R: Multiple functions for network graph construction and visualization (i.e. create boxplot diagrams with different thresholds, graph calculation, adapt ratio for heatmap construction, plot heatmaps, plot graphs, calculate discrete weights, calculate degree-based weights, calculate graph object, plot pBI scores as bar charts). 6. *.txt files: Contain coordinates for graph visualization. For further information please read the “ReadMe.txt” file in the Supporting information. (ZIP) [file pone.0208953.s004.zip › S1_File/output/Example_Plots/02_Graphs/DynThresh/q90/Network_t240_q90dyn.png]

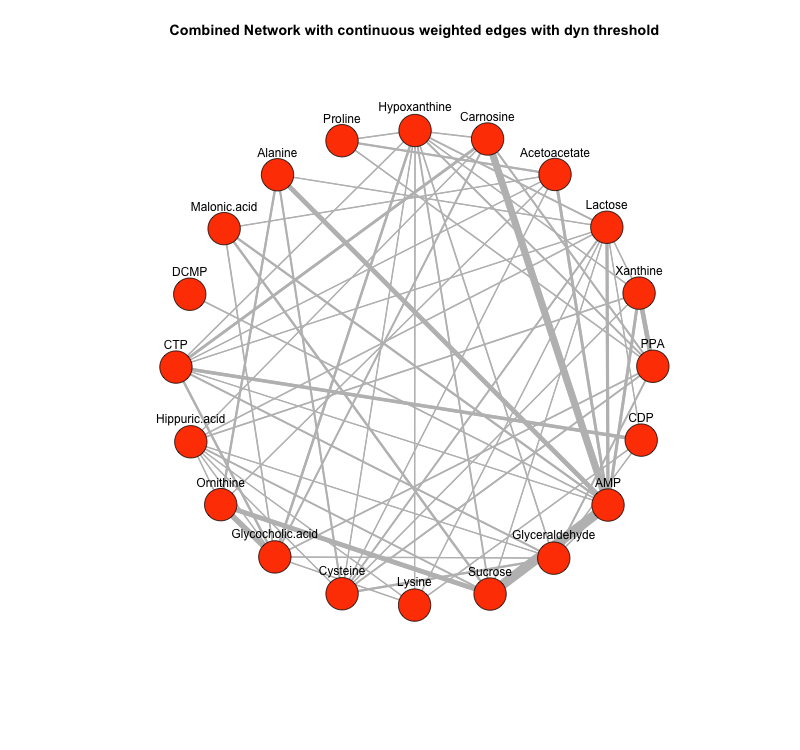

Supplement: S1 File — The R-based computational framework for data preprocessing, metabolite subset selection and dynamic network construction consists of the following R-scripts and text files: 1. Main.R: Main script for the analysis of metabolic data in order to identify putative biomarker candidates based on dynamic network visualization. 2. Preprocessing.R: (i) Removes metabolites with more than 60% of the values missing from the dataset; (ii) Replaces missing values with the metabolite's median at a given time point for the remaining dataset; (iii) Creates a data subset, containing only those metabolites, which are present at all time points. 3. BI.R: Function that calculates pBI scores for all metabolites. 4. InferBIGraph.R: Function to sum up the function calls for the calculation of a network graph. 5. FunctionsGraph.R: Multiple functions for network graph construction and visualization (i.e. create boxplot diagrams with different thresholds, graph calculation, adapt ratio for heatmap construction, plot heatmaps, plot graphs, calculate discrete weights, calculate degree-based weights, calculate graph object, plot pBI scores as bar charts). 6. *.txt files: Contain coordinates for graph visualization. For further information please read the “ReadMe.txt” file in the Supporting information. (ZIP) [file pone.0208953.s004.zip › S1_File/output/Example_Plots/02_Graphs/DynThresh/q90/poly4/Network_continuous_weighted_4_graphs_q90dyn.png]

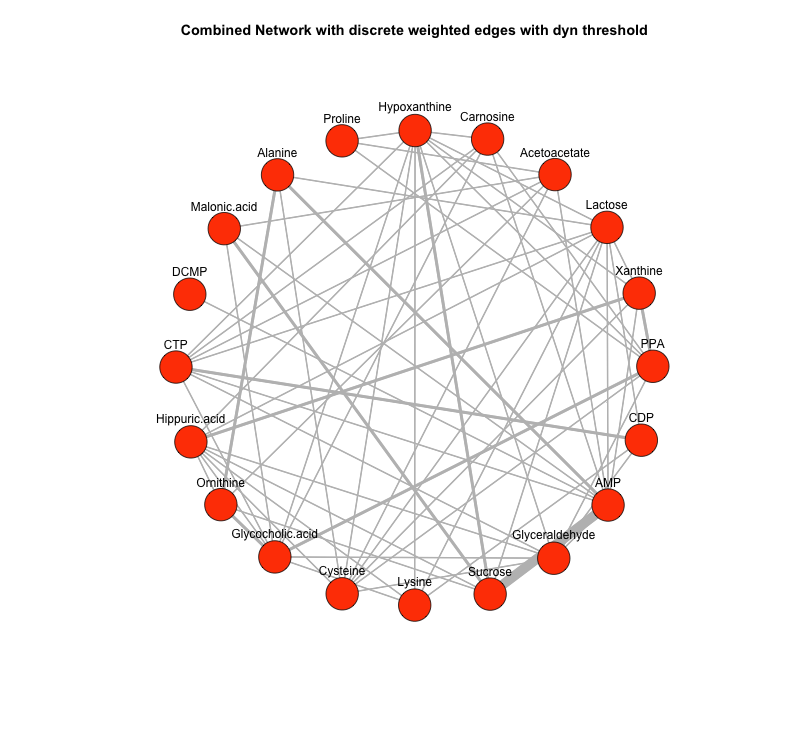

Supplement: S1 File — The R-based computational framework for data preprocessing, metabolite subset selection and dynamic network construction consists of the following R-scripts and text files: 1. Main.R: Main script for the analysis of metabolic data in order to identify putative biomarker candidates based on dynamic network visualization. 2. Preprocessing.R: (i) Removes metabolites with more than 60% of the values missing from the dataset; (ii) Replaces missing values with the metabolite's median at a given time point for the remaining dataset; (iii) Creates a data subset, containing only those metabolites, which are present at all time points. 3. BI.R: Function that calculates pBI scores for all metabolites. 4. InferBIGraph.R: Function to sum up the function calls for the calculation of a network graph. 5. FunctionsGraph.R: Multiple functions for network graph construction and visualization (i.e. create boxplot diagrams with different thresholds, graph calculation, adapt ratio for heatmap construction, plot heatmaps, plot graphs, calculate discrete weights, calculate degree-based weights, calculate graph object, plot pBI scores as bar charts). 6. *.txt files: Contain coordinates for graph visualization. For further information please read the “ReadMe.txt” file in the Supporting information. (ZIP) [file pone.0208953.s004.zip › S1_File/output/Example_Plots/02_Graphs/DynThresh/q90/poly4/Network_discrete_weighted_4_graphs_q90dyn.png]

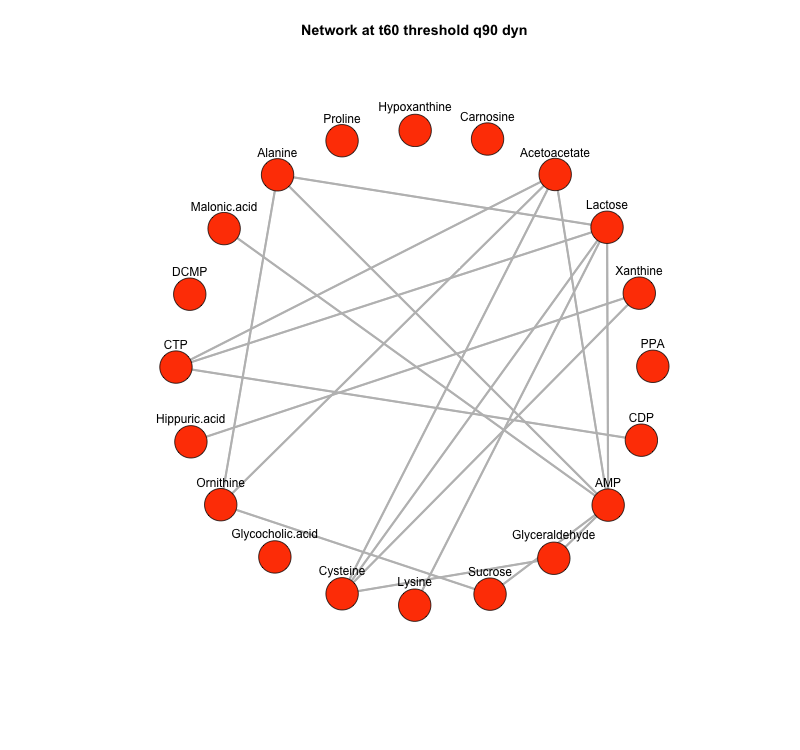

Supplement: S1 File — The R-based computational framework for data preprocessing, metabolite subset selection and dynamic network construction consists of the following R-scripts and text files: 1. Main.R: Main script for the analysis of metabolic data in order to identify putative biomarker candidates based on dynamic network visualization. 2. Preprocessing.R: (i) Removes metabolites with more than 60% of the values missing from the dataset; (ii) Replaces missing values with the metabolite's median at a given time point for the remaining dataset; (iii) Creates a data subset, containing only those metabolites, which are present at all time points. 3. BI.R: Function that calculates pBI scores for all metabolites. 4. InferBIGraph.R: Function to sum up the function calls for the calculation of a network graph. 5. FunctionsGraph.R: Multiple functions for network graph construction and visualization (i.e. create boxplot diagrams with different thresholds, graph calculation, adapt ratio for heatmap construction, plot heatmaps, plot graphs, calculate discrete weights, calculate degree-based weights, calculate graph object, plot pBI scores as bar charts). 6. *.txt files: Contain coordinates for graph visualization. For further information please read the “ReadMe.txt” file in the Supporting information. (ZIP) [file pone.0208953.s004.zip › S1_File/output/Example_Plots/02_Graphs/DynThresh/q90/Network_t60_q90dyn.png]

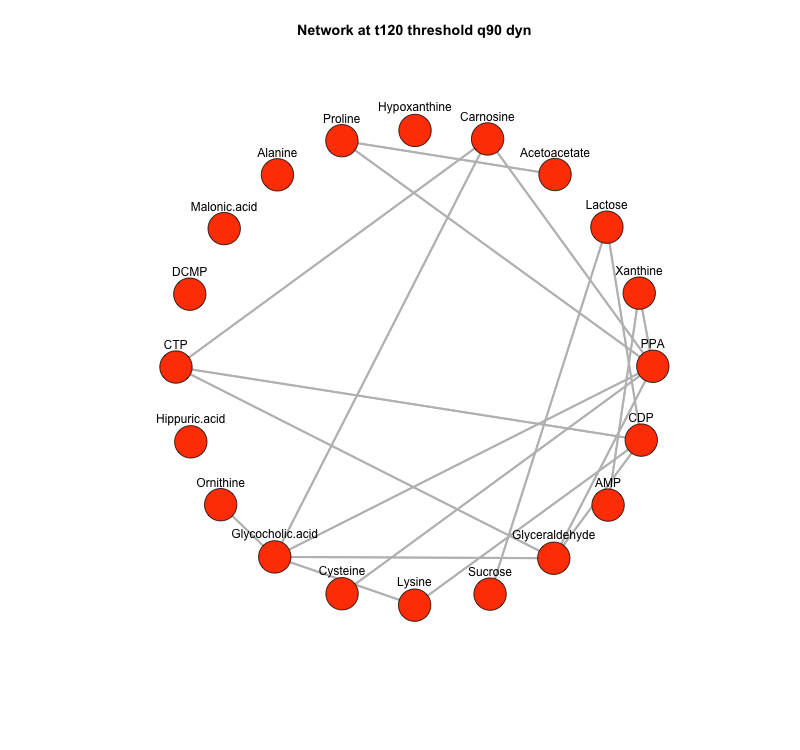

Supplement: S1 File — The R-based computational framework for data preprocessing, metabolite subset selection and dynamic network construction consists of the following R-scripts and text files: 1. Main.R: Main script for the analysis of metabolic data in order to identify putative biomarker candidates based on dynamic network visualization. 2. Preprocessing.R: (i) Removes metabolites with more than 60% of the values missing from the dataset; (ii) Replaces missing values with the metabolite's median at a given time point for the remaining dataset; (iii) Creates a data subset, containing only those metabolites, which are present at all time points. 3. BI.R: Function that calculates pBI scores for all metabolites. 4. InferBIGraph.R: Function to sum up the function calls for the calculation of a network graph. 5. FunctionsGraph.R: Multiple functions for network graph construction and visualization (i.e. create boxplot diagrams with different thresholds, graph calculation, adapt ratio for heatmap construction, plot heatmaps, plot graphs, calculate discrete weights, calculate degree-based weights, calculate graph object, plot pBI scores as bar charts). 6. *.txt files: Contain coordinates for graph visualization. For further information please read the “ReadMe.txt” file in the Supporting information. (ZIP) [file pone.0208953.s004.zip › S1_File/output/Example_Plots/02_Graphs/DynThresh/q90/Network_t120_q90dyn.png]

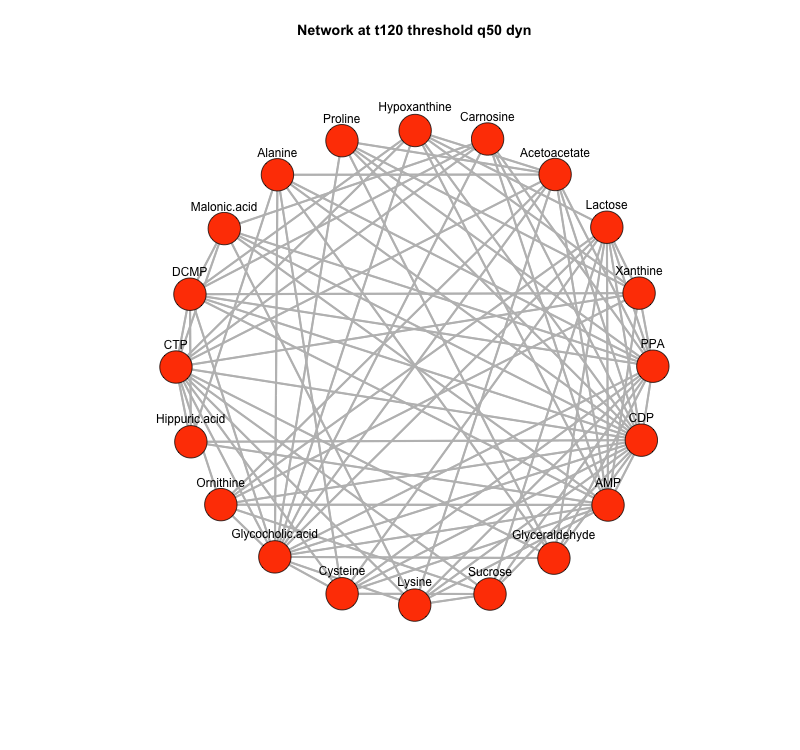

Supplement: S1 File — The R-based computational framework for data preprocessing, metabolite subset selection and dynamic network construction consists of the following R-scripts and text files: 1. Main.R: Main script for the analysis of metabolic data in order to identify putative biomarker candidates based on dynamic network visualization. 2. Preprocessing.R: (i) Removes metabolites with more than 60% of the values missing from the dataset; (ii) Replaces missing values with the metabolite's median at a given time point for the remaining dataset; (iii) Creates a data subset, containing only those metabolites, which are present at all time points. 3. BI.R: Function that calculates pBI scores for all metabolites. 4. InferBIGraph.R: Function to sum up the function calls for the calculation of a network graph. 5. FunctionsGraph.R: Multiple functions for network graph construction and visualization (i.e. create boxplot diagrams with different thresholds, graph calculation, adapt ratio for heatmap construction, plot heatmaps, plot graphs, calculate discrete weights, calculate degree-based weights, calculate graph object, plot pBI scores as bar charts). 6. *.txt files: Contain coordinates for graph visualization. For further information please read the “ReadMe.txt” file in the Supporting information. (ZIP) [file pone.0208953.s004.zip › S1_File/output/Example_Plots/02_Graphs/DynThresh/q50/Network_t120_q50dyn.png]

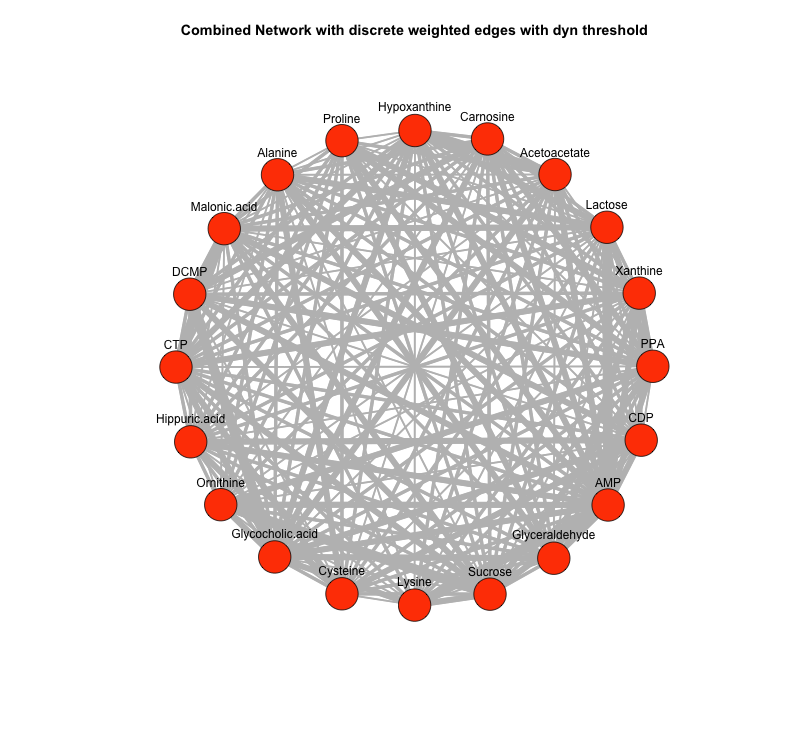

Supplement: S1 File — The R-based computational framework for data preprocessing, metabolite subset selection and dynamic network construction consists of the following R-scripts and text files: 1. Main.R: Main script for the analysis of metabolic data in order to identify putative biomarker candidates based on dynamic network visualization. 2. Preprocessing.R: (i) Removes metabolites with more than 60% of the values missing from the dataset; (ii) Replaces missing values with the metabolite's median at a given time point for the remaining dataset; (iii) Creates a data subset, containing only those metabolites, which are present at all time points. 3. BI.R: Function that calculates pBI scores for all metabolites. 4. InferBIGraph.R: Function to sum up the function calls for the calculation of a network graph. 5. FunctionsGraph.R: Multiple functions for network graph construction and visualization (i.e. create boxplot diagrams with different thresholds, graph calculation, adapt ratio for heatmap construction, plot heatmaps, plot graphs, calculate discrete weights, calculate degree-based weights, calculate graph object, plot pBI scores as bar charts). 6. *.txt files: Contain coordinates for graph visualization. For further information please read the “ReadMe.txt” file in the Supporting information. (ZIP) [file pone.0208953.s004.zip › S1_File/output/Example_Plots/02_Graphs/DynThresh/q50/quad/Network_discrete_weighted_4_graphs_q50dyn.png]

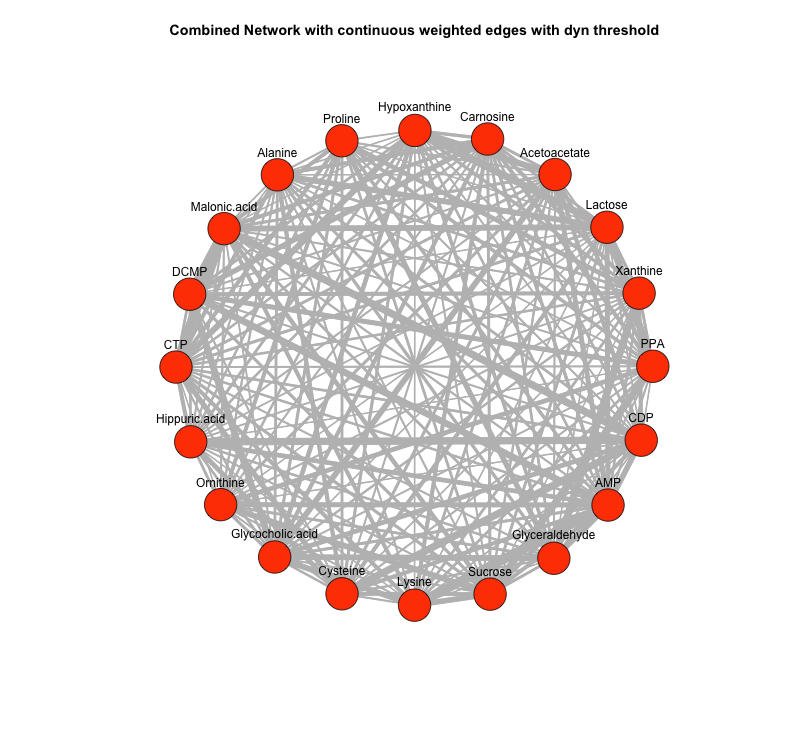

Supplement: S1 File — The R-based computational framework for data preprocessing, metabolite subset selection and dynamic network construction consists of the following R-scripts and text files: 1. Main.R: Main script for the analysis of metabolic data in order to identify putative biomarker candidates based on dynamic network visualization. 2. Preprocessing.R: (i) Removes metabolites with more than 60% of the values missing from the dataset; (ii) Replaces missing values with the metabolite's median at a given time point for the remaining dataset; (iii) Creates a data subset, containing only those metabolites, which are present at all time points. 3. BI.R: Function that calculates pBI scores for all metabolites. 4. InferBIGraph.R: Function to sum up the function calls for the calculation of a network graph. 5. FunctionsGraph.R: Multiple functions for network graph construction and visualization (i.e. create boxplot diagrams with different thresholds, graph calculation, adapt ratio for heatmap construction, plot heatmaps, plot graphs, calculate discrete weights, calculate degree-based weights, calculate graph object, plot pBI scores as bar charts). 6. *.txt files: Contain coordinates for graph visualization. For further information please read the “ReadMe.txt” file in the Supporting information. (ZIP) [file pone.0208953.s004.zip › S1_File/output/Example_Plots/02_Graphs/DynThresh/q50/quad/Network_continuous_weighted_4_graphs_q50dyn.png]

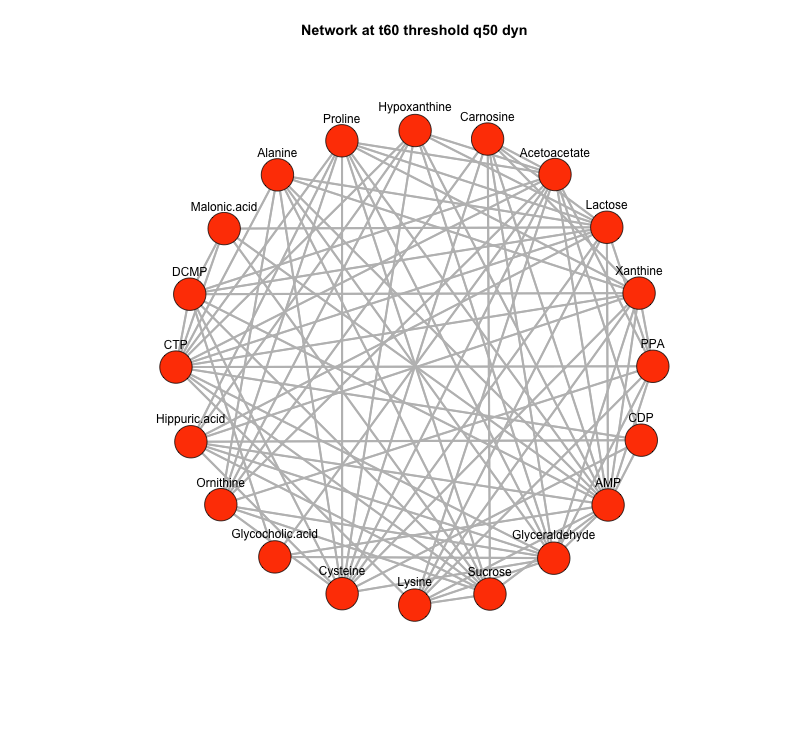

Supplement: S1 File — The R-based computational framework for data preprocessing, metabolite subset selection and dynamic network construction consists of the following R-scripts and text files: 1. Main.R: Main script for the analysis of metabolic data in order to identify putative biomarker candidates based on dynamic network visualization. 2. Preprocessing.R: (i) Removes metabolites with more than 60% of the values missing from the dataset; (ii) Replaces missing values with the metabolite's median at a given time point for the remaining dataset; (iii) Creates a data subset, containing only those metabolites, which are present at all time points. 3. BI.R: Function that calculates pBI scores for all metabolites. 4. InferBIGraph.R: Function to sum up the function calls for the calculation of a network graph. 5. FunctionsGraph.R: Multiple functions for network graph construction and visualization (i.e. create boxplot diagrams with different thresholds, graph calculation, adapt ratio for heatmap construction, plot heatmaps, plot graphs, calculate discrete weights, calculate degree-based weights, calculate graph object, plot pBI scores as bar charts). 6. *.txt files: Contain coordinates for graph visualization. For further information please read the “ReadMe.txt” file in the Supporting information. (ZIP) [file pone.0208953.s004.zip › S1_File/output/Example_Plots/02_Graphs/DynThresh/q50/Network_t60_q50dyn.png]

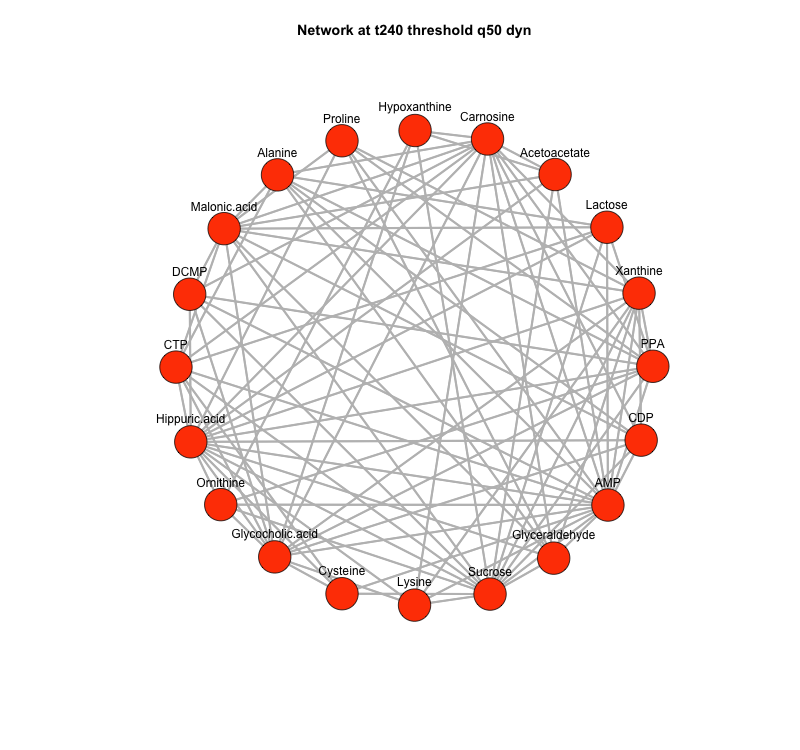

Supplement: S1 File — The R-based computational framework for data preprocessing, metabolite subset selection and dynamic network construction consists of the following R-scripts and text files: 1. Main.R: Main script for the analysis of metabolic data in order to identify putative biomarker candidates based on dynamic network visualization. 2. Preprocessing.R: (i) Removes metabolites with more than 60% of the values missing from the dataset; (ii) Replaces missing values with the metabolite's median at a given time point for the remaining dataset; (iii) Creates a data subset, containing only those metabolites, which are present at all time points. 3. BI.R: Function that calculates pBI scores for all metabolites. 4. InferBIGraph.R: Function to sum up the function calls for the calculation of a network graph. 5. FunctionsGraph.R: Multiple functions for network graph construction and visualization (i.e. create boxplot diagrams with different thresholds, graph calculation, adapt ratio for heatmap construction, plot heatmaps, plot graphs, calculate discrete weights, calculate degree-based weights, calculate graph object, plot pBI scores as bar charts). 6. *.txt files: Contain coordinates for graph visualization. For further information please read the “ReadMe.txt” file in the Supporting information. (ZIP) [file pone.0208953.s004.zip › S1_File/output/Example_Plots/02_Graphs/DynThresh/q50/Network_t240_q50dyn.png]

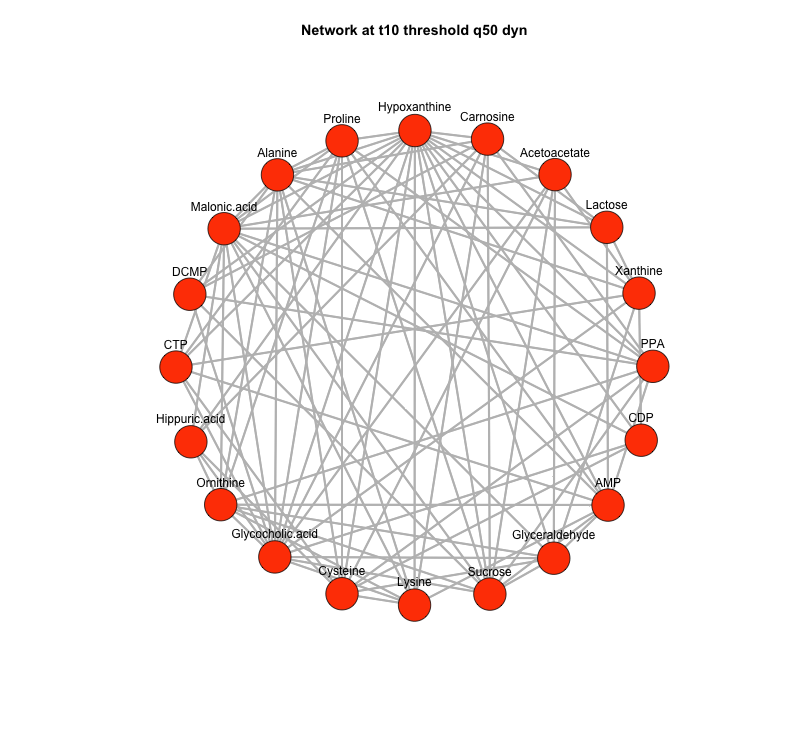

Supplement: S1 File — The R-based computational framework for data preprocessing, metabolite subset selection and dynamic network construction consists of the following R-scripts and text files: 1. Main.R: Main script for the analysis of metabolic data in order to identify putative biomarker candidates based on dynamic network visualization. 2. Preprocessing.R: (i) Removes metabolites with more than 60% of the values missing from the dataset; (ii) Replaces missing values with the metabolite's median at a given time point for the remaining dataset; (iii) Creates a data subset, containing only those metabolites, which are present at all time points. 3. BI.R: Function that calculates pBI scores for all metabolites. 4. InferBIGraph.R: Function to sum up the function calls for the calculation of a network graph. 5. FunctionsGraph.R: Multiple functions for network graph construction and visualization (i.e. create boxplot diagrams with different thresholds, graph calculation, adapt ratio for heatmap construction, plot heatmaps, plot graphs, calculate discrete weights, calculate degree-based weights, calculate graph object, plot pBI scores as bar charts). 6. *.txt files: Contain coordinates for graph visualization. For further information please read the “ReadMe.txt” file in the Supporting information. (ZIP) [file pone.0208953.s004.zip › S1_File/output/Example_Plots/02_Graphs/DynThresh/q50/Network_t10_q50dyn.png]

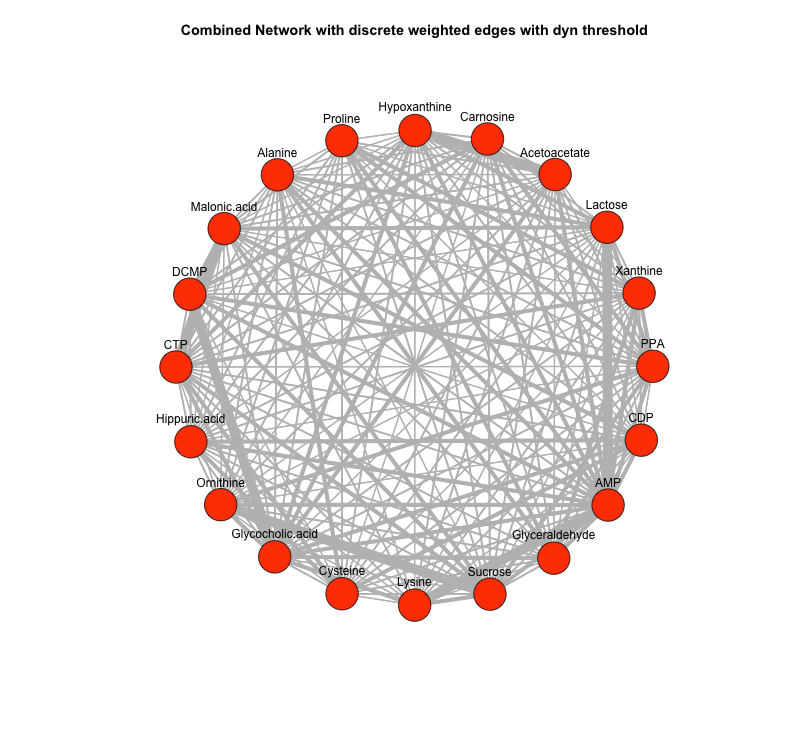

Supplement: S1 File — The R-based computational framework for data preprocessing, metabolite subset selection and dynamic network construction consists of the following R-scripts and text files: 1. Main.R: Main script for the analysis of metabolic data in order to identify putative biomarker candidates based on dynamic network visualization. 2. Preprocessing.R: (i) Removes metabolites with more than 60% of the values missing from the dataset; (ii) Replaces missing values with the metabolite's median at a given time point for the remaining dataset; (iii) Creates a data subset, containing only those metabolites, which are present at all time points. 3. BI.R: Function that calculates pBI scores for all metabolites. 4. InferBIGraph.R: Function to sum up the function calls for the calculation of a network graph. 5. FunctionsGraph.R: Multiple functions for network graph construction and visualization (i.e. create boxplot diagrams with different thresholds, graph calculation, adapt ratio for heatmap construction, plot heatmaps, plot graphs, calculate discrete weights, calculate degree-based weights, calculate graph object, plot pBI scores as bar charts). 6. *.txt files: Contain coordinates for graph visualization. For further information please read the “ReadMe.txt” file in the Supporting information. (ZIP) [file pone.0208953.s004.zip › S1_File/output/Example_Plots/02_Graphs/DynThresh/q50/poly4/Network_discrete_weighted_4_graphs_q50dyn.png]

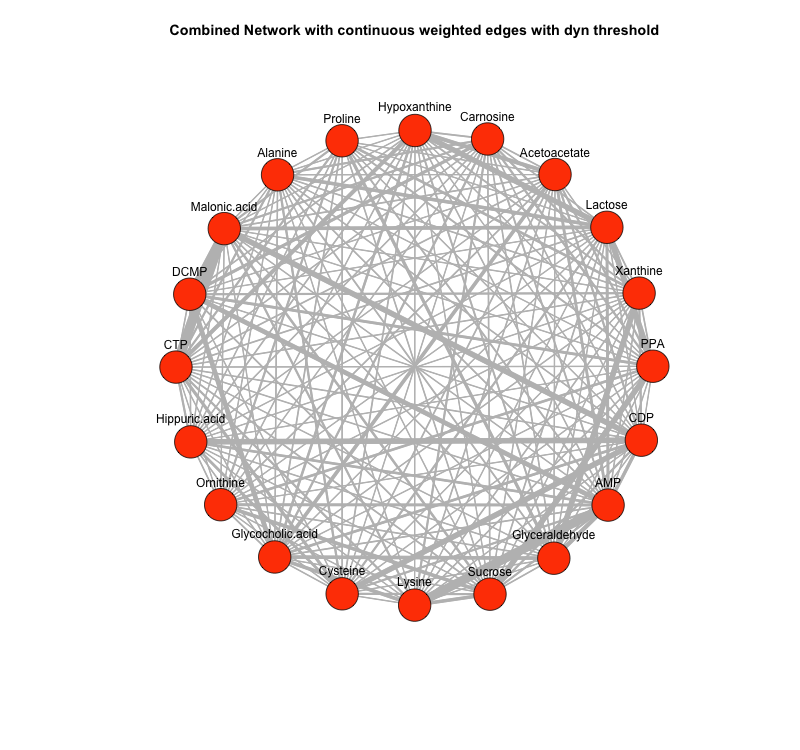

Supplement: S1 File — The R-based computational framework for data preprocessing, metabolite subset selection and dynamic network construction consists of the following R-scripts and text files: 1. Main.R: Main script for the analysis of metabolic data in order to identify putative biomarker candidates based on dynamic network visualization. 2. Preprocessing.R: (i) Removes metabolites with more than 60% of the values missing from the dataset; (ii) Replaces missing values with the metabolite's median at a given time point for the remaining dataset; (iii) Creates a data subset, containing only those metabolites, which are present at all time points. 3. BI.R: Function that calculates pBI scores for all metabolites. 4. InferBIGraph.R: Function to sum up the function calls for the calculation of a network graph. 5. FunctionsGraph.R: Multiple functions for network graph construction and visualization (i.e. create boxplot diagrams with different thresholds, graph calculation, adapt ratio for heatmap construction, plot heatmaps, plot graphs, calculate discrete weights, calculate degree-based weights, calculate graph object, plot pBI scores as bar charts). 6. *.txt files: Contain coordinates for graph visualization. For further information please read the “ReadMe.txt” file in the Supporting information. (ZIP) [file pone.0208953.s004.zip › S1_File/output/Example_Plots/02_Graphs/DynThresh/q50/poly4/Network_continuous_weighted_4_graphs_q50dyn.png]

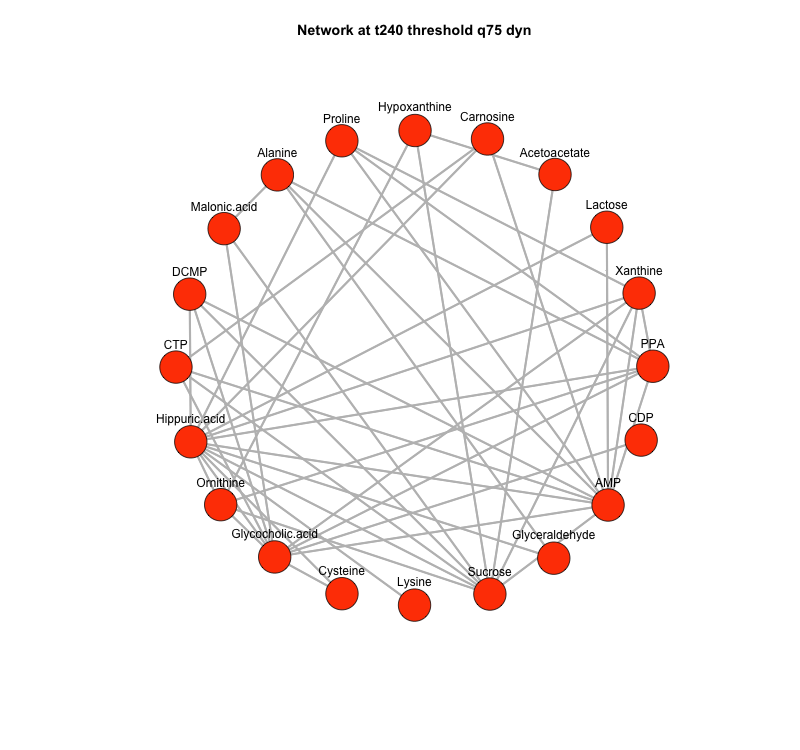

Supplement: S1 File — The R-based computational framework for data preprocessing, metabolite subset selection and dynamic network construction consists of the following R-scripts and text files: 1. Main.R: Main script for the analysis of metabolic data in order to identify putative biomarker candidates based on dynamic network visualization. 2. Preprocessing.R: (i) Removes metabolites with more than 60% of the values missing from the dataset; (ii) Replaces missing values with the metabolite's median at a given time point for the remaining dataset; (iii) Creates a data subset, containing only those metabolites, which are present at all time points. 3. BI.R: Function that calculates pBI scores for all metabolites. 4. InferBIGraph.R: Function to sum up the function calls for the calculation of a network graph. 5. FunctionsGraph.R: Multiple functions for network graph construction and visualization (i.e. create boxplot diagrams with different thresholds, graph calculation, adapt ratio for heatmap construction, plot heatmaps, plot graphs, calculate discrete weights, calculate degree-based weights, calculate graph object, plot pBI scores as bar charts). 6. *.txt files: Contain coordinates for graph visualization. For further information please read the “ReadMe.txt” file in the Supporting information. (ZIP) [file pone.0208953.s004.zip › S1_File/output/Example_Plots/02_Graphs/DynThresh/q75/Network_t240_q75dyn.png]

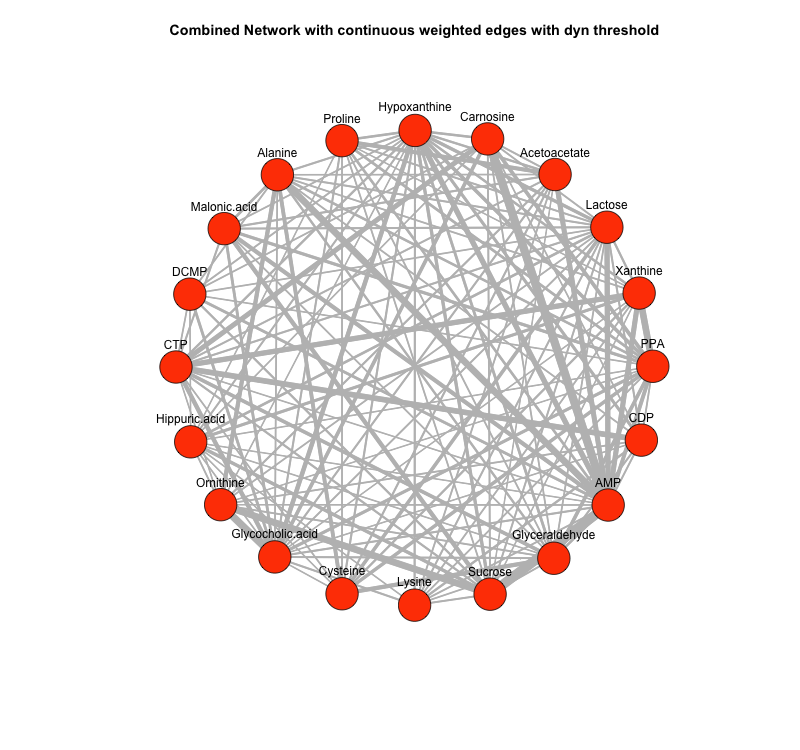

Supplement: S1 File — The R-based computational framework for data preprocessing, metabolite subset selection and dynamic network construction consists of the following R-scripts and text files: 1. Main.R: Main script for the analysis of metabolic data in order to identify putative biomarker candidates based on dynamic network visualization. 2. Preprocessing.R: (i) Removes metabolites with more than 60% of the values missing from the dataset; (ii) Replaces missing values with the metabolite's median at a given time point for the remaining dataset; (iii) Creates a data subset, containing only those metabolites, which are present at all time points. 3. BI.R: Function that calculates pBI scores for all metabolites. 4. InferBIGraph.R: Function to sum up the function calls for the calculation of a network graph. 5. FunctionsGraph.R: Multiple functions for network graph construction and visualization (i.e. create boxplot diagrams with different thresholds, graph calculation, adapt ratio for heatmap construction, plot heatmaps, plot graphs, calculate discrete weights, calculate degree-based weights, calculate graph object, plot pBI scores as bar charts). 6. *.txt files: Contain coordinates for graph visualization. For further information please read the “ReadMe.txt” file in the Supporting information. (ZIP) [file pone.0208953.s004.zip › S1_File/output/Example_Plots/02_Graphs/DynThresh/q75/quad/Network_continuous_weighted_4_graphs_q75dyn.png]

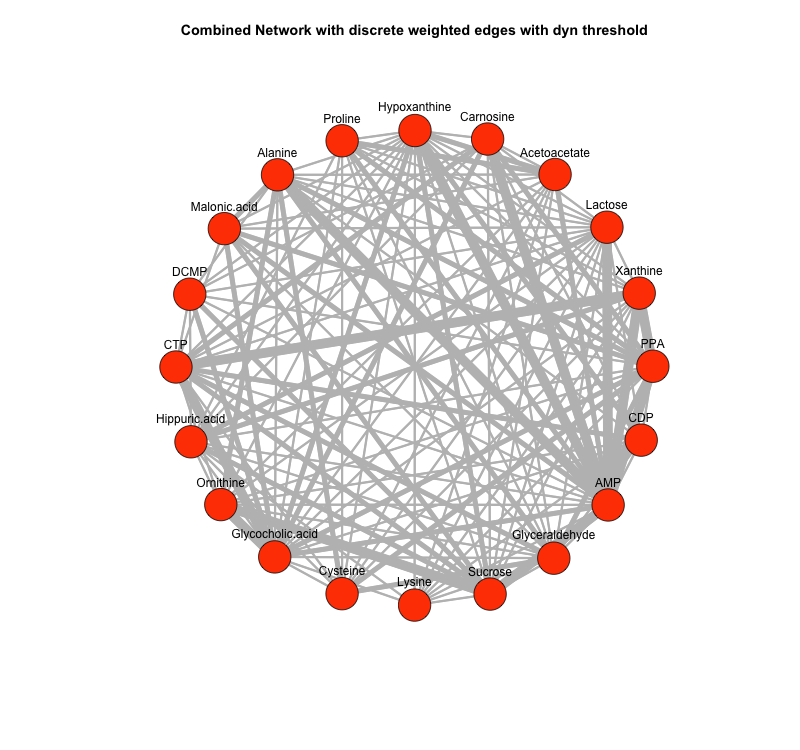

Supplement: S1 File — The R-based computational framework for data preprocessing, metabolite subset selection and dynamic network construction consists of the following R-scripts and text files: 1. Main.R: Main script for the analysis of metabolic data in order to identify putative biomarker candidates based on dynamic network visualization. 2. Preprocessing.R: (i) Removes metabolites with more than 60% of the values missing from the dataset; (ii) Replaces missing values with the metabolite's median at a given time point for the remaining dataset; (iii) Creates a data subset, containing only those metabolites, which are present at all time points. 3. BI.R: Function that calculates pBI scores for all metabolites. 4. InferBIGraph.R: Function to sum up the function calls for the calculation of a network graph. 5. FunctionsGraph.R: Multiple functions for network graph construction and visualization (i.e. create boxplot diagrams with different thresholds, graph calculation, adapt ratio for heatmap construction, plot heatmaps, plot graphs, calculate discrete weights, calculate degree-based weights, calculate graph object, plot pBI scores as bar charts). 6. *.txt files: Contain coordinates for graph visualization. For further information please read the “ReadMe.txt” file in the Supporting information. (ZIP) [file pone.0208953.s004.zip › S1_File/output/Example_Plots/02_Graphs/DynThresh/q75/quad/Network_discrete_weighted_4_graphs_q75dyn.png]

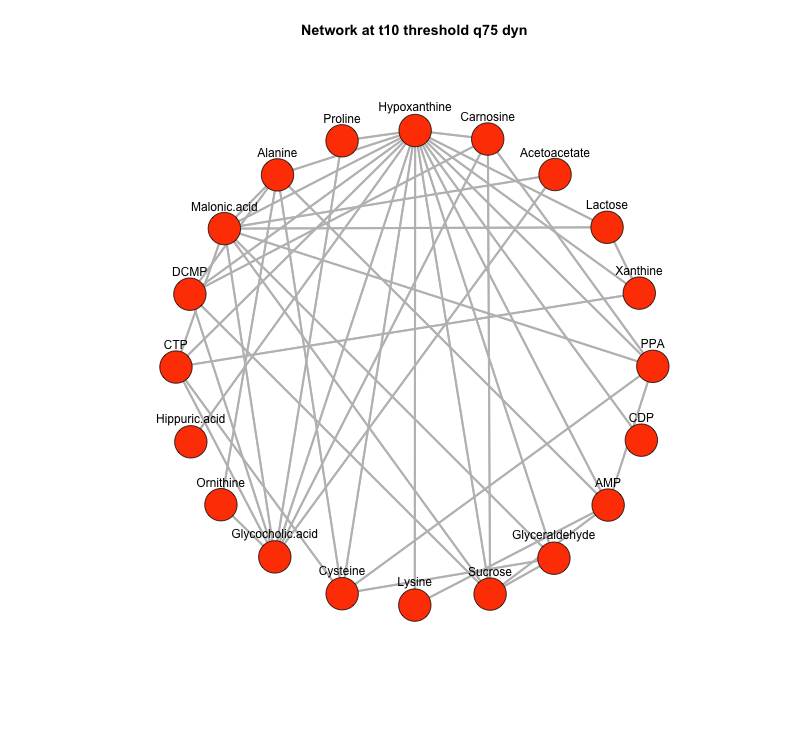

Supplement: S1 File — The R-based computational framework for data preprocessing, metabolite subset selection and dynamic network construction consists of the following R-scripts and text files: 1. Main.R: Main script for the analysis of metabolic data in order to identify putative biomarker candidates based on dynamic network visualization. 2. Preprocessing.R: (i) Removes metabolites with more than 60% of the values missing from the dataset; (ii) Replaces missing values with the metabolite's median at a given time point for the remaining dataset; (iii) Creates a data subset, containing only those metabolites, which are present at all time points. 3. BI.R: Function that calculates pBI scores for all metabolites. 4. InferBIGraph.R: Function to sum up the function calls for the calculation of a network graph. 5. FunctionsGraph.R: Multiple functions for network graph construction and visualization (i.e. create boxplot diagrams with different thresholds, graph calculation, adapt ratio for heatmap construction, plot heatmaps, plot graphs, calculate discrete weights, calculate degree-based weights, calculate graph object, plot pBI scores as bar charts). 6. *.txt files: Contain coordinates for graph visualization. For further information please read the “ReadMe.txt” file in the Supporting information. (ZIP) [file pone.0208953.s004.zip › S1_File/output/Example_Plots/02_Graphs/DynThresh/q75/Network_t10_q75dyn.png]

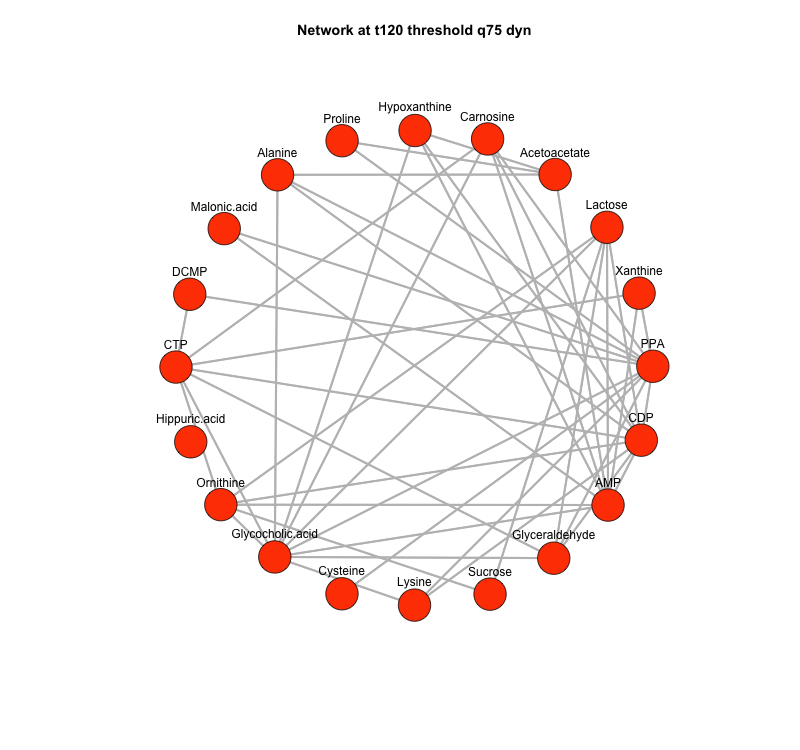

Supplement: S1 File — The R-based computational framework for data preprocessing, metabolite subset selection and dynamic network construction consists of the following R-scripts and text files: 1. Main.R: Main script for the analysis of metabolic data in order to identify putative biomarker candidates based on dynamic network visualization. 2. Preprocessing.R: (i) Removes metabolites with more than 60% of the values missing from the dataset; (ii) Replaces missing values with the metabolite's median at a given time point for the remaining dataset; (iii) Creates a data subset, containing only those metabolites, which are present at all time points. 3. BI.R: Function that calculates pBI scores for all metabolites. 4. InferBIGraph.R: Function to sum up the function calls for the calculation of a network graph. 5. FunctionsGraph.R: Multiple functions for network graph construction and visualization (i.e. create boxplot diagrams with different thresholds, graph calculation, adapt ratio for heatmap construction, plot heatmaps, plot graphs, calculate discrete weights, calculate degree-based weights, calculate graph object, plot pBI scores as bar charts). 6. *.txt files: Contain coordinates for graph visualization. For further information please read the “ReadMe.txt” file in the Supporting information. (ZIP) [file pone.0208953.s004.zip › S1_File/output/Example_Plots/02_Graphs/DynThresh/q75/Network_t120_q75dyn.png]

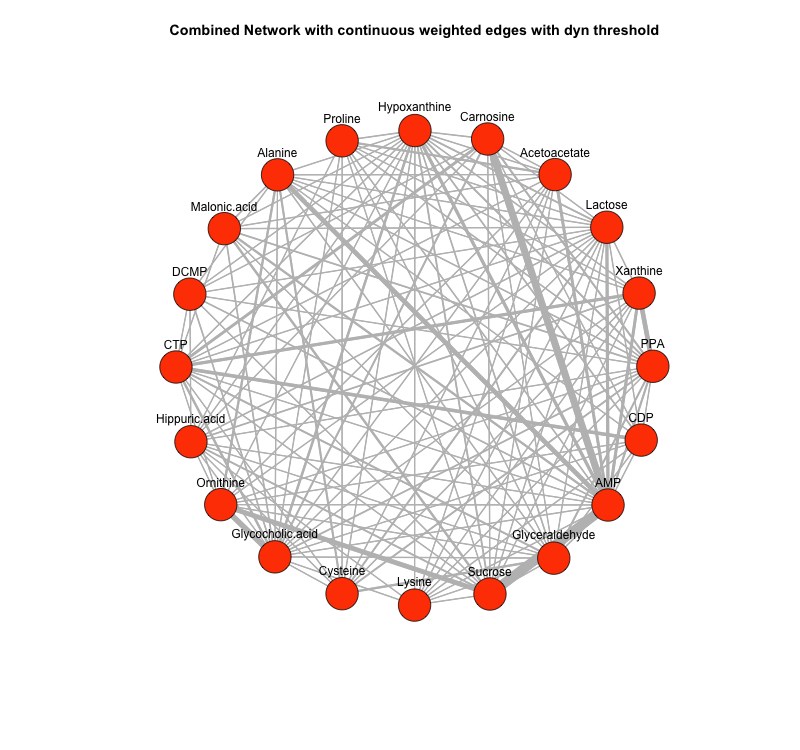

Supplement: S1 File — The R-based computational framework for data preprocessing, metabolite subset selection and dynamic network construction consists of the following R-scripts and text files: 1. Main.R: Main script for the analysis of metabolic data in order to identify putative biomarker candidates based on dynamic network visualization. 2. Preprocessing.R: (i) Removes metabolites with more than 60% of the values missing from the dataset; (ii) Replaces missing values with the metabolite's median at a given time point for the remaining dataset; (iii) Creates a data subset, containing only those metabolites, which are present at all time points. 3. BI.R: Function that calculates pBI scores for all metabolites. 4. InferBIGraph.R: Function to sum up the function calls for the calculation of a network graph. 5. FunctionsGraph.R: Multiple functions for network graph construction and visualization (i.e. create boxplot diagrams with different thresholds, graph calculation, adapt ratio for heatmap construction, plot heatmaps, plot graphs, calculate discrete weights, calculate degree-based weights, calculate graph object, plot pBI scores as bar charts). 6. *.txt files: Contain coordinates for graph visualization. For further information please read the “ReadMe.txt” file in the Supporting information. (ZIP) [file pone.0208953.s004.zip › S1_File/output/Example_Plots/02_Graphs/DynThresh/q75/poly4/Network_continuous_weighted_4_graphs_q75dyn.png]

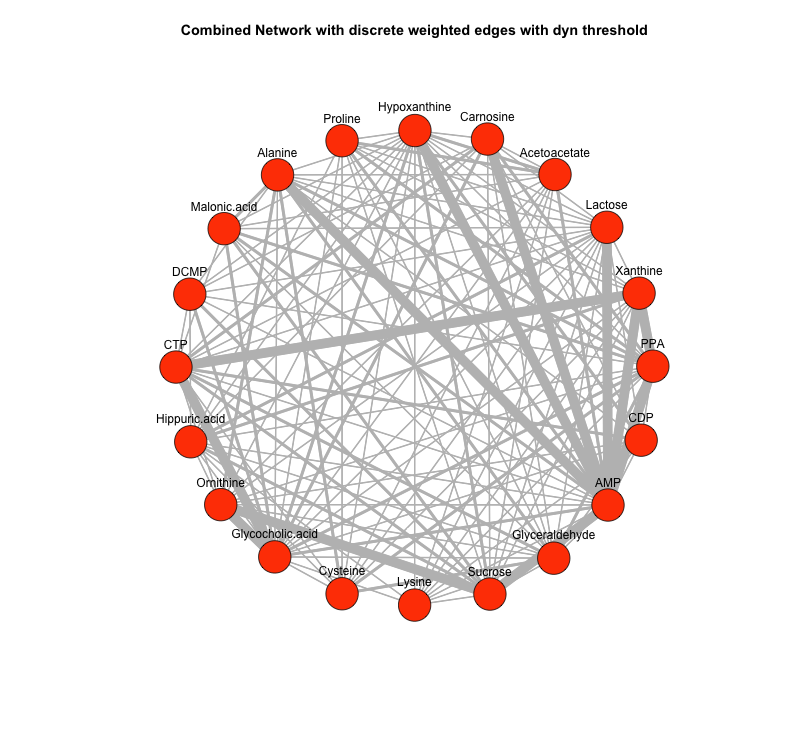

Supplement: S1 File — The R-based computational framework for data preprocessing, metabolite subset selection and dynamic network construction consists of the following R-scripts and text files: 1. Main.R: Main script for the analysis of metabolic data in order to identify putative biomarker candidates based on dynamic network visualization. 2. Preprocessing.R: (i) Removes metabolites with more than 60% of the values missing from the dataset; (ii) Replaces missing values with the metabolite's median at a given time point for the remaining dataset; (iii) Creates a data subset, containing only those metabolites, which are present at all time points. 3. BI.R: Function that calculates pBI scores for all metabolites. 4. InferBIGraph.R: Function to sum up the function calls for the calculation of a network graph. 5. FunctionsGraph.R: Multiple functions for network graph construction and visualization (i.e. create boxplot diagrams with different thresholds, graph calculation, adapt ratio for heatmap construction, plot heatmaps, plot graphs, calculate discrete weights, calculate degree-based weights, calculate graph object, plot pBI scores as bar charts). 6. *.txt files: Contain coordinates for graph visualization. For further information please read the “ReadMe.txt” file in the Supporting information. (ZIP) [file pone.0208953.s004.zip › S1_File/output/Example_Plots/02_Graphs/DynThresh/q75/poly4/Network_discrete_weighted_4_graphs_q75dyn.png]

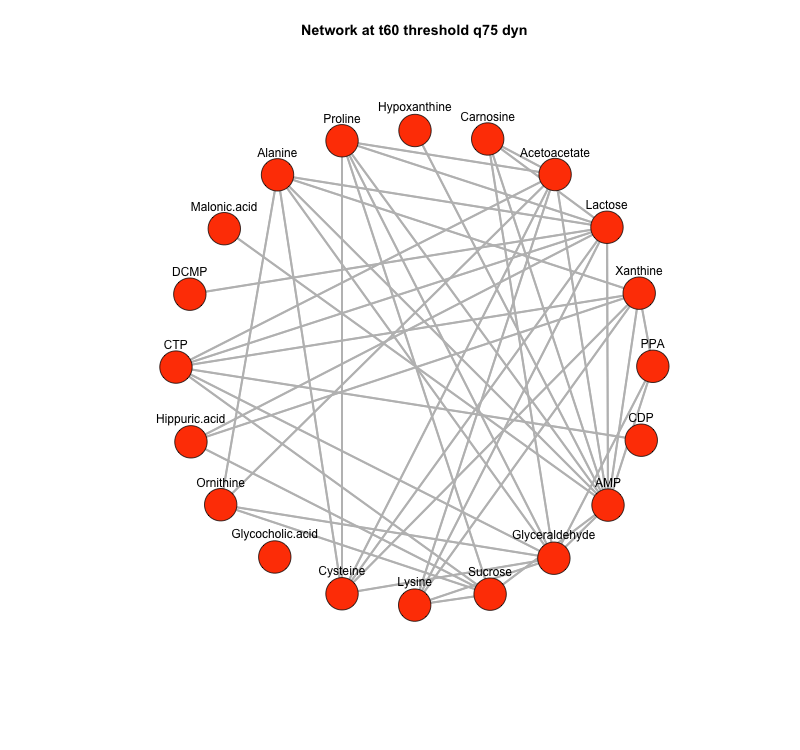

Supplement: S1 File — The R-based computational framework for data preprocessing, metabolite subset selection and dynamic network construction consists of the following R-scripts and text files: 1. Main.R: Main script for the analysis of metabolic data in order to identify putative biomarker candidates based on dynamic network visualization. 2. Preprocessing.R: (i) Removes metabolites with more than 60% of the values missing from the dataset; (ii) Replaces missing values with the metabolite's median at a given time point for the remaining dataset; (iii) Creates a data subset, containing only those metabolites, which are present at all time points. 3. BI.R: Function that calculates pBI scores for all metabolites. 4. InferBIGraph.R: Function to sum up the function calls for the calculation of a network graph. 5. FunctionsGraph.R: Multiple functions for network graph construction and visualization (i.e. create boxplot diagrams with different thresholds, graph calculation, adapt ratio for heatmap construction, plot heatmaps, plot graphs, calculate discrete weights, calculate degree-based weights, calculate graph object, plot pBI scores as bar charts). 6. *.txt files: Contain coordinates for graph visualization. For further information please read the “ReadMe.txt” file in the Supporting information. (ZIP) [file pone.0208953.s004.zip › S1_File/output/Example_Plots/02_Graphs/DynThresh/q75/Network_t60_q75dyn.png]

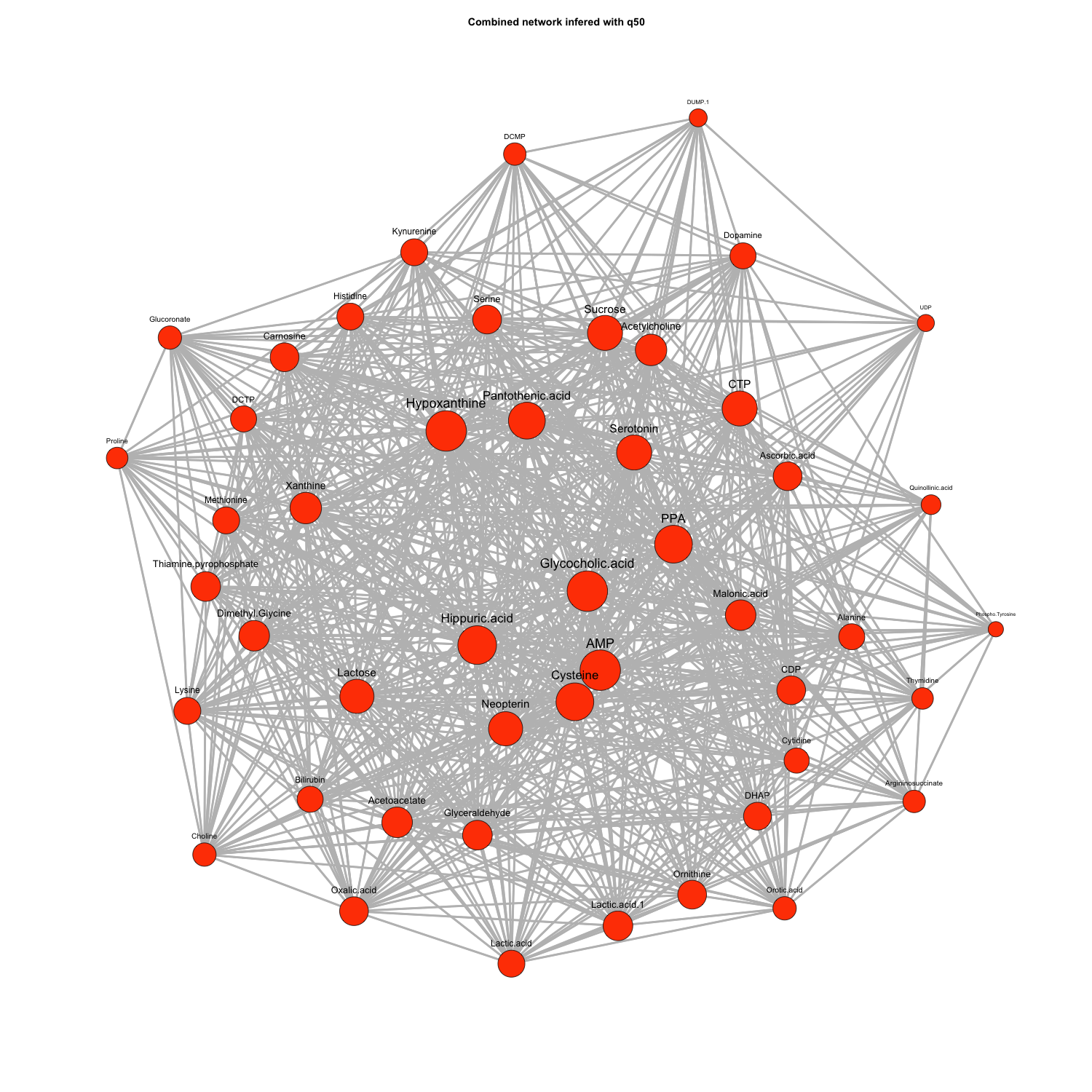

Supplement: S1 File — The R-based computational framework for data preprocessing, metabolite subset selection and dynamic network construction consists of the following R-scripts and text files: 1. Main.R: Main script for the analysis of metabolic data in order to identify putative biomarker candidates based on dynamic network visualization. 2. Preprocessing.R: (i) Removes metabolites with more than 60% of the values missing from the dataset; (ii) Replaces missing values with the metabolite's median at a given time point for the remaining dataset; (iii) Creates a data subset, containing only those metabolites, which are present at all time points. 3. BI.R: Function that calculates pBI scores for all metabolites. 4. InferBIGraph.R: Function to sum up the function calls for the calculation of a network graph. 5. FunctionsGraph.R: Multiple functions for network graph construction and visualization (i.e. create boxplot diagrams with different thresholds, graph calculation, adapt ratio for heatmap construction, plot heatmaps, plot graphs, calculate discrete weights, calculate degree-based weights, calculate graph object, plot pBI scores as bar charts). 6. *.txt files: Contain coordinates for graph visualization. For further information please read the “ReadMe.txt” file in the Supporting information. (ZIP) [file pone.0208953.s004.zip › S1_File/output/Example_Plots/02_Graphs/Overview_Combined_q50.png]

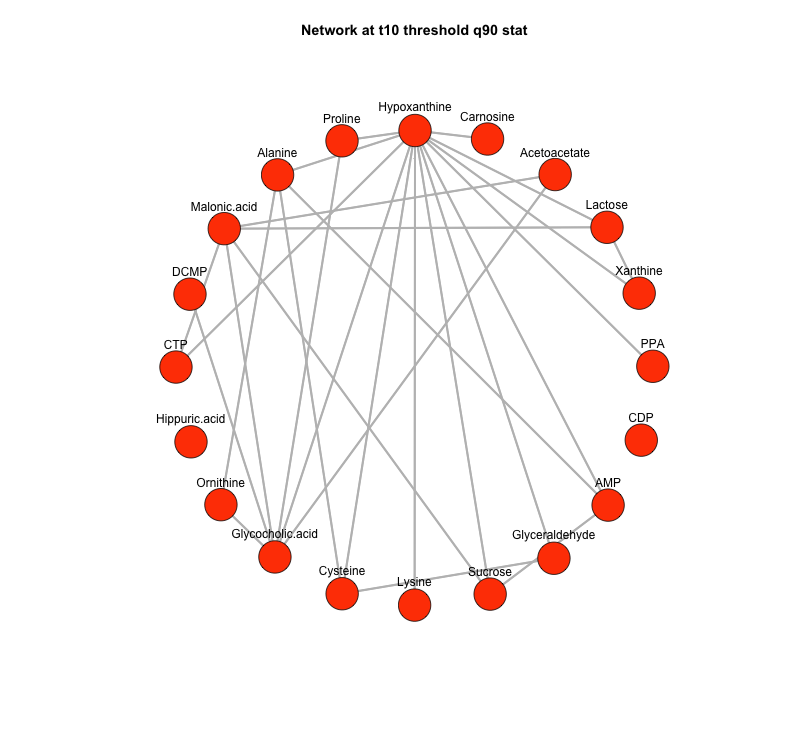

Supplement: S1 File — The R-based computational framework for data preprocessing, metabolite subset selection and dynamic network construction consists of the following R-scripts and text files: 1. Main.R: Main script for the analysis of metabolic data in order to identify putative biomarker candidates based on dynamic network visualization. 2. Preprocessing.R: (i) Removes metabolites with more than 60% of the values missing from the dataset; (ii) Replaces missing values with the metabolite's median at a given time point for the remaining dataset; (iii) Creates a data subset, containing only those metabolites, which are present at all time points. 3. BI.R: Function that calculates pBI scores for all metabolites. 4. InferBIGraph.R: Function to sum up the function calls for the calculation of a network graph. 5. FunctionsGraph.R: Multiple functions for network graph construction and visualization (i.e. create boxplot diagrams with different thresholds, graph calculation, adapt ratio for heatmap construction, plot heatmaps, plot graphs, calculate discrete weights, calculate degree-based weights, calculate graph object, plot pBI scores as bar charts). 6. *.txt files: Contain coordinates for graph visualization. For further information please read the “ReadMe.txt” file in the Supporting information. (ZIP) [file pone.0208953.s004.zip › S1_File/output/Example_Plots/02_Graphs/StatThresh/q90/Network_t10_q90stat.png]

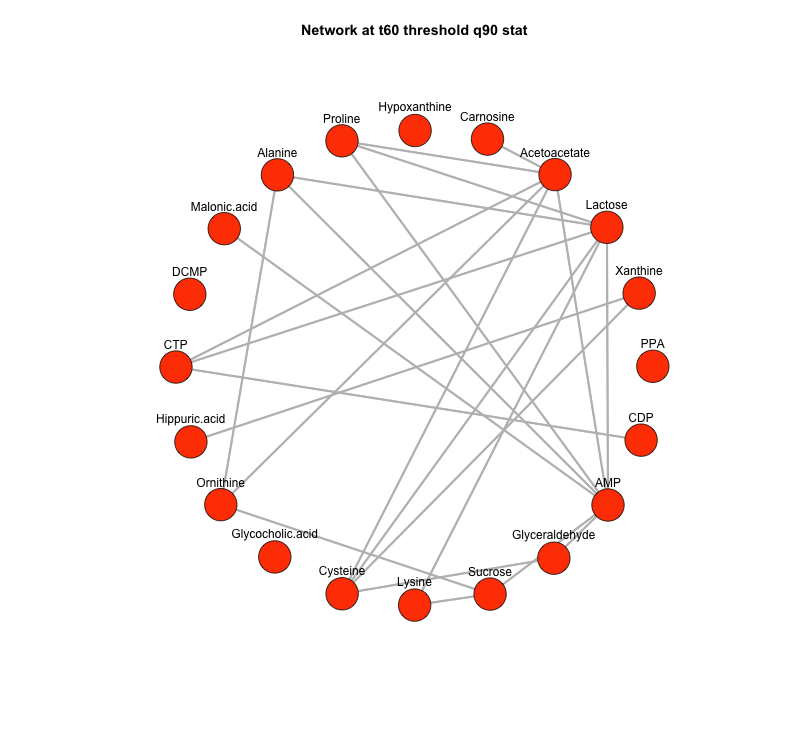

Supplement: S1 File — The R-based computational framework for data preprocessing, metabolite subset selection and dynamic network construction consists of the following R-scripts and text files: 1. Main.R: Main script for the analysis of metabolic data in order to identify putative biomarker candidates based on dynamic network visualization. 2. Preprocessing.R: (i) Removes metabolites with more than 60% of the values missing from the dataset; (ii) Replaces missing values with the metabolite's median at a given time point for the remaining dataset; (iii) Creates a data subset, containing only those metabolites, which are present at all time points. 3. BI.R: Function that calculates pBI scores for all metabolites. 4. InferBIGraph.R: Function to sum up the function calls for the calculation of a network graph. 5. FunctionsGraph.R: Multiple functions for network graph construction and visualization (i.e. create boxplot diagrams with different thresholds, graph calculation, adapt ratio for heatmap construction, plot heatmaps, plot graphs, calculate discrete weights, calculate degree-based weights, calculate graph object, plot pBI scores as bar charts). 6. *.txt files: Contain coordinates for graph visualization. For further information please read the “ReadMe.txt” file in the Supporting information. (ZIP) [file pone.0208953.s004.zip › S1_File/output/Example_Plots/02_Graphs/StatThresh/q90/Network_t60_q90stat.png]

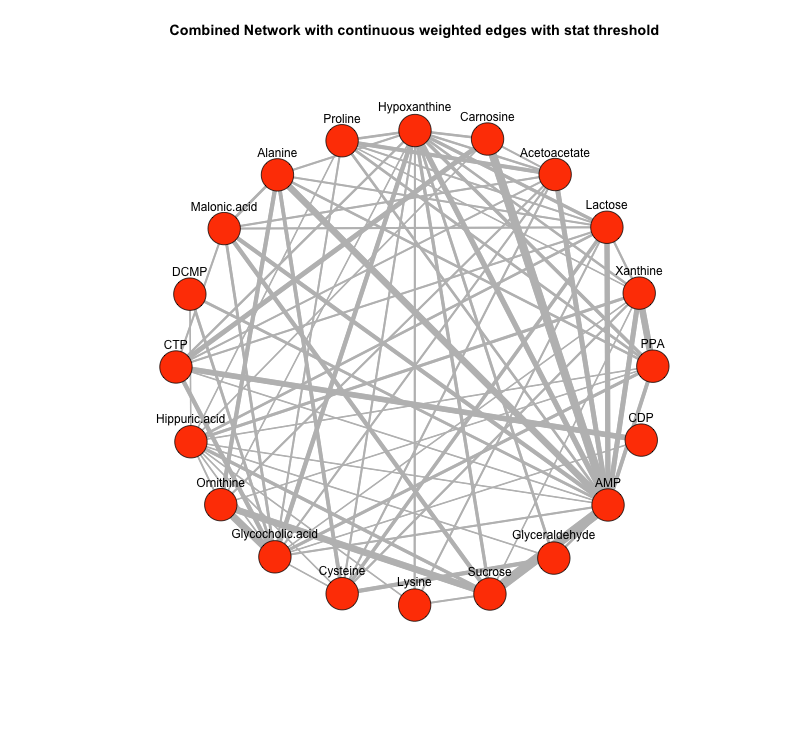

Supplement: S1 File — The R-based computational framework for data preprocessing, metabolite subset selection and dynamic network construction consists of the following R-scripts and text files: 1. Main.R: Main script for the analysis of metabolic data in order to identify putative biomarker candidates based on dynamic network visualization. 2. Preprocessing.R: (i) Removes metabolites with more than 60% of the values missing from the dataset; (ii) Replaces missing values with the metabolite's median at a given time point for the remaining dataset; (iii) Creates a data subset, containing only those metabolites, which are present at all time points. 3. BI.R: Function that calculates pBI scores for all metabolites. 4. InferBIGraph.R: Function to sum up the function calls for the calculation of a network graph. 5. FunctionsGraph.R: Multiple functions for network graph construction and visualization (i.e. create boxplot diagrams with different thresholds, graph calculation, adapt ratio for heatmap construction, plot heatmaps, plot graphs, calculate discrete weights, calculate degree-based weights, calculate graph object, plot pBI scores as bar charts). 6. *.txt files: Contain coordinates for graph visualization. For further information please read the “ReadMe.txt” file in the Supporting information. (ZIP) [file pone.0208953.s004.zip › S1_File/output/Example_Plots/02_Graphs/StatThresh/q90/quad/Network_continuous_weighted_4_graphs_q90stat.png]

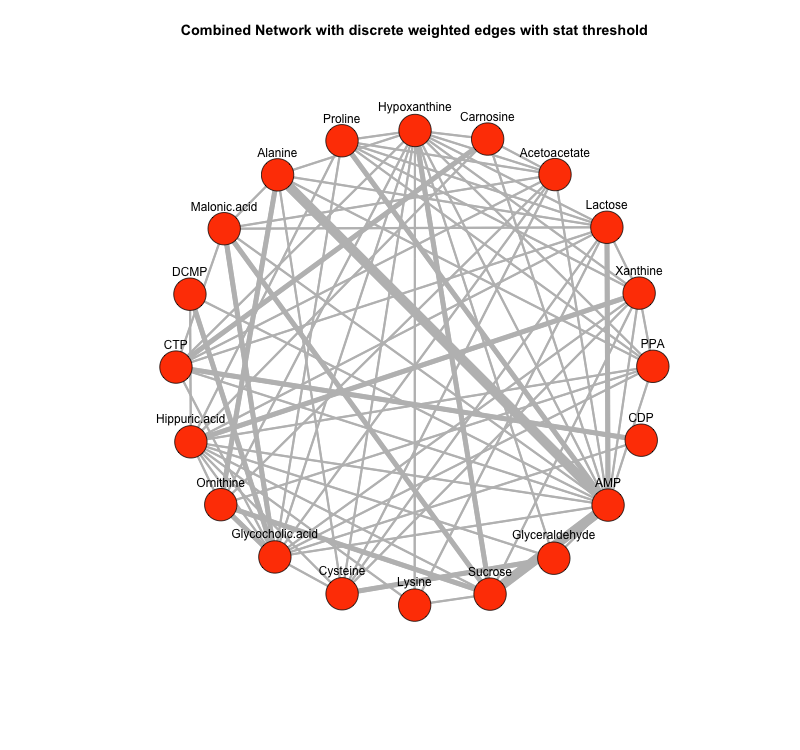

Supplement: S1 File — The R-based computational framework for data preprocessing, metabolite subset selection and dynamic network construction consists of the following R-scripts and text files: 1. Main.R: Main script for the analysis of metabolic data in order to identify putative biomarker candidates based on dynamic network visualization. 2. Preprocessing.R: (i) Removes metabolites with more than 60% of the values missing from the dataset; (ii) Replaces missing values with the metabolite's median at a given time point for the remaining dataset; (iii) Creates a data subset, containing only those metabolites, which are present at all time points. 3. BI.R: Function that calculates pBI scores for all metabolites. 4. InferBIGraph.R: Function to sum up the function calls for the calculation of a network graph. 5. FunctionsGraph.R: Multiple functions for network graph construction and visualization (i.e. create boxplot diagrams with different thresholds, graph calculation, adapt ratio for heatmap construction, plot heatmaps, plot graphs, calculate discrete weights, calculate degree-based weights, calculate graph object, plot pBI scores as bar charts). 6. *.txt files: Contain coordinates for graph visualization. For further information please read the “ReadMe.txt” file in the Supporting information. (ZIP) [file pone.0208953.s004.zip › S1_File/output/Example_Plots/02_Graphs/StatThresh/q90/quad/Network_discrete_weighted_4_graphs_q90stat.png]

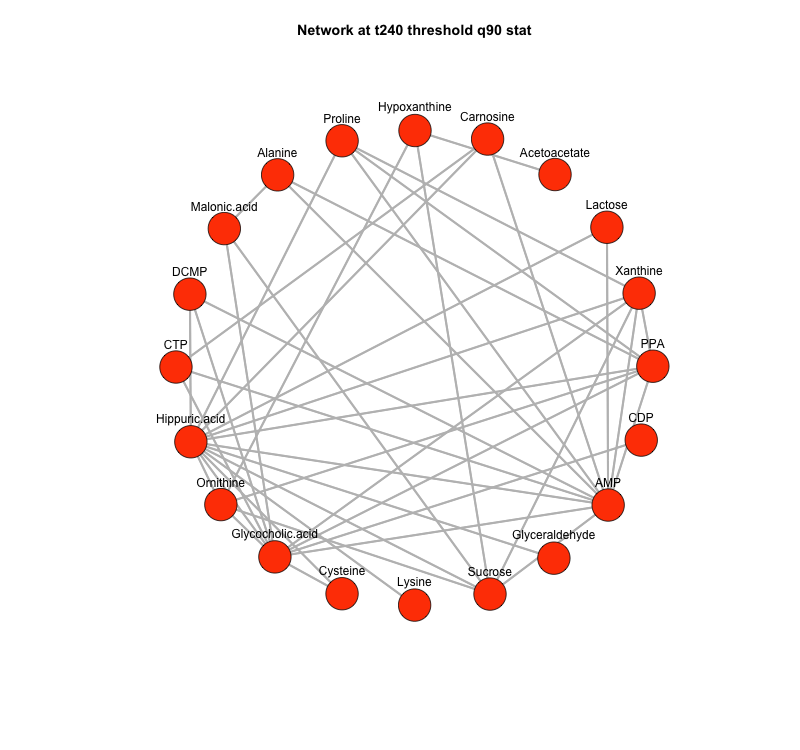

Supplement: S1 File — The R-based computational framework for data preprocessing, metabolite subset selection and dynamic network construction consists of the following R-scripts and text files: 1. Main.R: Main script for the analysis of metabolic data in order to identify putative biomarker candidates based on dynamic network visualization. 2. Preprocessing.R: (i) Removes metabolites with more than 60% of the values missing from the dataset; (ii) Replaces missing values with the metabolite's median at a given time point for the remaining dataset; (iii) Creates a data subset, containing only those metabolites, which are present at all time points. 3. BI.R: Function that calculates pBI scores for all metabolites. 4. InferBIGraph.R: Function to sum up the function calls for the calculation of a network graph. 5. FunctionsGraph.R: Multiple functions for network graph construction and visualization (i.e. create boxplot diagrams with different thresholds, graph calculation, adapt ratio for heatmap construction, plot heatmaps, plot graphs, calculate discrete weights, calculate degree-based weights, calculate graph object, plot pBI scores as bar charts). 6. *.txt files: Contain coordinates for graph visualization. For further information please read the “ReadMe.txt” file in the Supporting information. (ZIP) [file pone.0208953.s004.zip › S1_File/output/Example_Plots/02_Graphs/StatThresh/q90/Network_t240_q90stat.png]

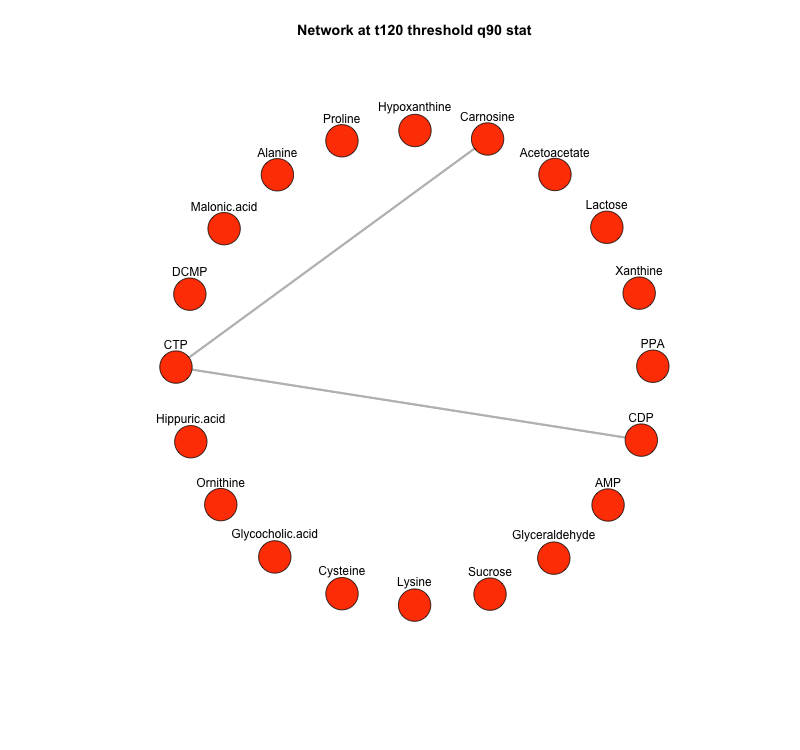

Supplement: S1 File — The R-based computational framework for data preprocessing, metabolite subset selection and dynamic network construction consists of the following R-scripts and text files: 1. Main.R: Main script for the analysis of metabolic data in order to identify putative biomarker candidates based on dynamic network visualization. 2. Preprocessing.R: (i) Removes metabolites with more than 60% of the values missing from the dataset; (ii) Replaces missing values with the metabolite's median at a given time point for the remaining dataset; (iii) Creates a data subset, containing only those metabolites, which are present at all time points. 3. BI.R: Function that calculates pBI scores for all metabolites. 4. InferBIGraph.R: Function to sum up the function calls for the calculation of a network graph. 5. FunctionsGraph.R: Multiple functions for network graph construction and visualization (i.e. create boxplot diagrams with different thresholds, graph calculation, adapt ratio for heatmap construction, plot heatmaps, plot graphs, calculate discrete weights, calculate degree-based weights, calculate graph object, plot pBI scores as bar charts). 6. *.txt files: Contain coordinates for graph visualization. For further information please read the “ReadMe.txt” file in the Supporting information. (ZIP) [file pone.0208953.s004.zip › S1_File/output/Example_Plots/02_Graphs/StatThresh/q90/Network_t120_q90stat.png]

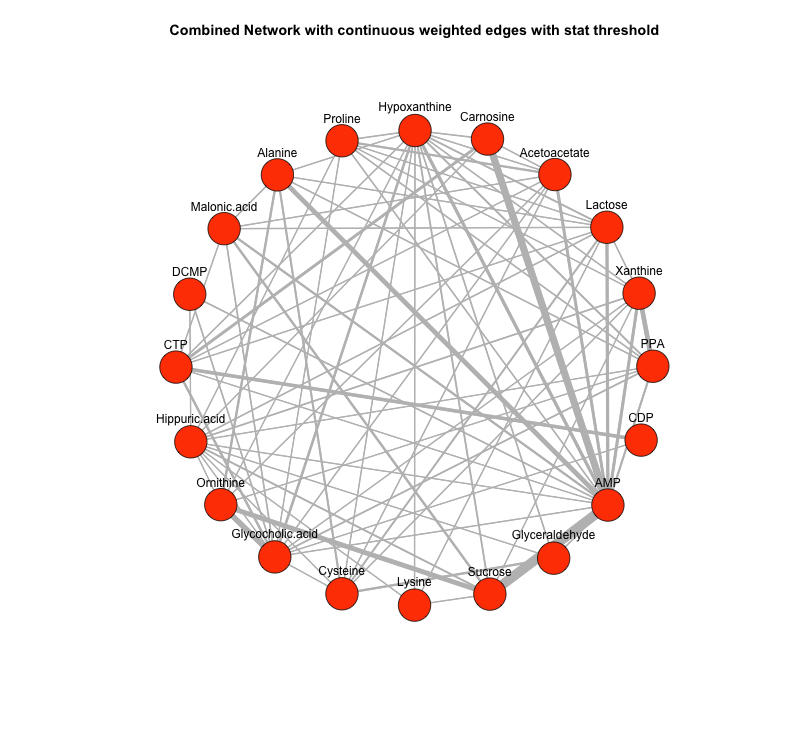

Supplement: S1 File — The R-based computational framework for data preprocessing, metabolite subset selection and dynamic network construction consists of the following R-scripts and text files: 1. Main.R: Main script for the analysis of metabolic data in order to identify putative biomarker candidates based on dynamic network visualization. 2. Preprocessing.R: (i) Removes metabolites with more than 60% of the values missing from the dataset; (ii) Replaces missing values with the metabolite's median at a given time point for the remaining dataset; (iii) Creates a data subset, containing only those metabolites, which are present at all time points. 3. BI.R: Function that calculates pBI scores for all metabolites. 4. InferBIGraph.R: Function to sum up the function calls for the calculation of a network graph. 5. FunctionsGraph.R: Multiple functions for network graph construction and visualization (i.e. create boxplot diagrams with different thresholds, graph calculation, adapt ratio for heatmap construction, plot heatmaps, plot graphs, calculate discrete weights, calculate degree-based weights, calculate graph object, plot pBI scores as bar charts). 6. *.txt files: Contain coordinates for graph visualization. For further information please read the “ReadMe.txt” file in the Supporting information. (ZIP) [file pone.0208953.s004.zip › S1_File/output/Example_Plots/02_Graphs/StatThresh/q90/poly4/Network_continuous_weighted_4_graphs_q90stat.png]

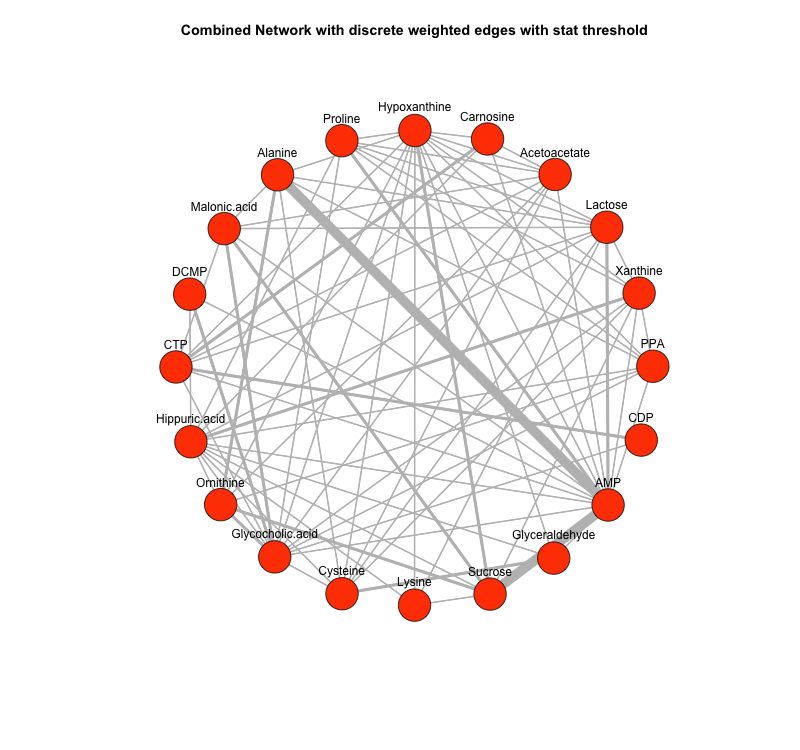

Supplement: S1 File — The R-based computational framework for data preprocessing, metabolite subset selection and dynamic network construction consists of the following R-scripts and text files: 1. Main.R: Main script for the analysis of metabolic data in order to identify putative biomarker candidates based on dynamic network visualization. 2. Preprocessing.R: (i) Removes metabolites with more than 60% of the values missing from the dataset; (ii) Replaces missing values with the metabolite's median at a given time point for the remaining dataset; (iii) Creates a data subset, containing only those metabolites, which are present at all time points. 3. BI.R: Function that calculates pBI scores for all metabolites. 4. InferBIGraph.R: Function to sum up the function calls for the calculation of a network graph. 5. FunctionsGraph.R: Multiple functions for network graph construction and visualization (i.e. create boxplot diagrams with different thresholds, graph calculation, adapt ratio for heatmap construction, plot heatmaps, plot graphs, calculate discrete weights, calculate degree-based weights, calculate graph object, plot pBI scores as bar charts). 6. *.txt files: Contain coordinates for graph visualization. For further information please read the “ReadMe.txt” file in the Supporting information. (ZIP) [file pone.0208953.s004.zip › S1_File/output/Example_Plots/02_Graphs/StatThresh/q90/poly4/Network_discrete_weighted_4_graphs_q90stat.png]

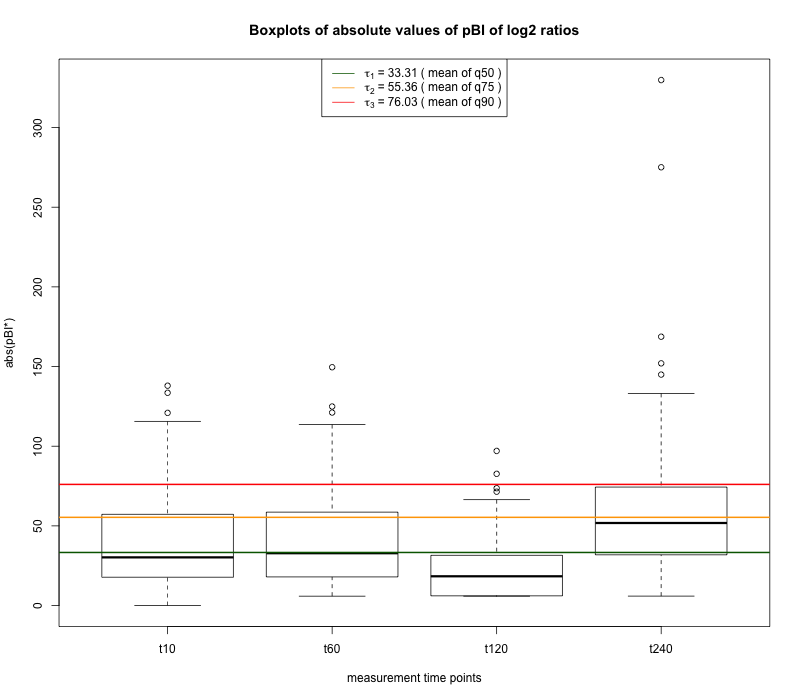

Supplement: S1 File — The R-based computational framework for data preprocessing, metabolite subset selection and dynamic network construction consists of the following R-scripts and text files: 1. Main.R: Main script for the analysis of metabolic data in order to identify putative biomarker candidates based on dynamic network visualization. 2. Preprocessing.R: (i) Removes metabolites with more than 60% of the values missing from the dataset; (ii) Replaces missing values with the metabolite's median at a given time point for the remaining dataset; (iii) Creates a data subset, containing only those metabolites, which are present at all time points. 3. BI.R: Function that calculates pBI scores for all metabolites. 4. InferBIGraph.R: Function to sum up the function calls for the calculation of a network graph. 5. FunctionsGraph.R: Multiple functions for network graph construction and visualization (i.e. create boxplot diagrams with different thresholds, graph calculation, adapt ratio for heatmap construction, plot heatmaps, plot graphs, calculate discrete weights, calculate degree-based weights, calculate graph object, plot pBI scores as bar charts). 6. *.txt files: Contain coordinates for graph visualization. For further information please read the “ReadMe.txt” file in the Supporting information. (ZIP) [file pone.0208953.s004.zip › S1_File/output/Example_Plots/02_Graphs/StatThresh/abs_pBI_thresholds.png]

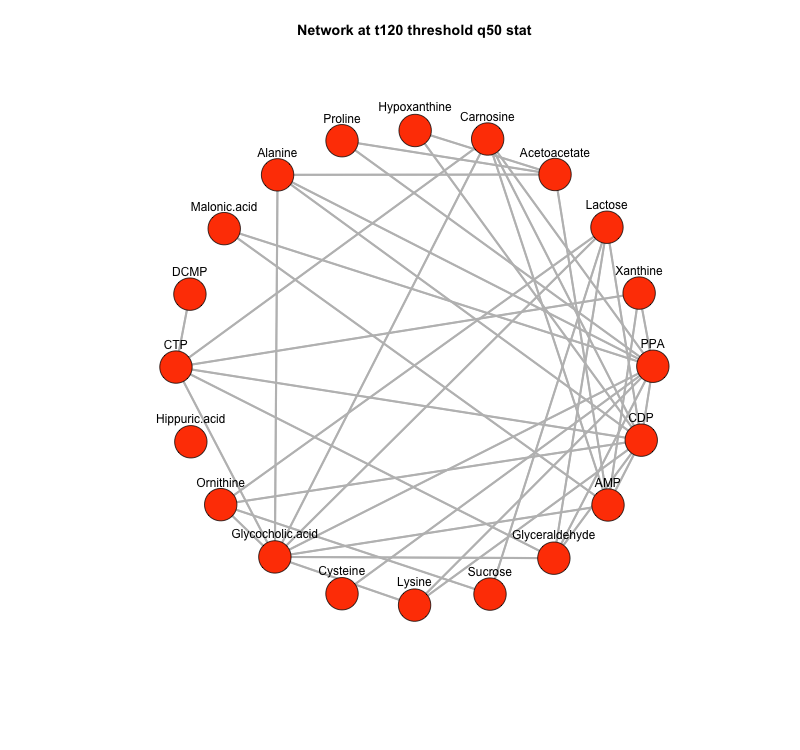

Supplement: S1 File — The R-based computational framework for data preprocessing, metabolite subset selection and dynamic network construction consists of the following R-scripts and text files: 1. Main.R: Main script for the analysis of metabolic data in order to identify putative biomarker candidates based on dynamic network visualization. 2. Preprocessing.R: (i) Removes metabolites with more than 60% of the values missing from the dataset; (ii) Replaces missing values with the metabolite's median at a given time point for the remaining dataset; (iii) Creates a data subset, containing only those metabolites, which are present at all time points. 3. BI.R: Function that calculates pBI scores for all metabolites. 4. InferBIGraph.R: Function to sum up the function calls for the calculation of a network graph. 5. FunctionsGraph.R: Multiple functions for network graph construction and visualization (i.e. create boxplot diagrams with different thresholds, graph calculation, adapt ratio for heatmap construction, plot heatmaps, plot graphs, calculate discrete weights, calculate degree-based weights, calculate graph object, plot pBI scores as bar charts). 6. *.txt files: Contain coordinates for graph visualization. For further information please read the “ReadMe.txt” file in the Supporting information. (ZIP) [file pone.0208953.s004.zip › S1_File/output/Example_Plots/02_Graphs/StatThresh/q50/Network_t120_q50stat.png]

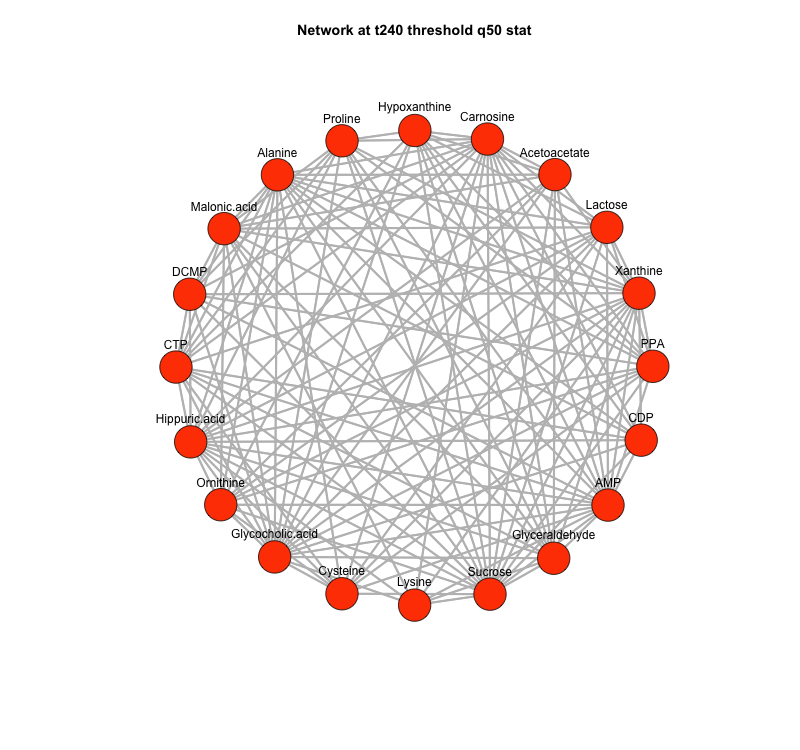

Supplement: S1 File — The R-based computational framework for data preprocessing, metabolite subset selection and dynamic network construction consists of the following R-scripts and text files: 1. Main.R: Main script for the analysis of metabolic data in order to identify putative biomarker candidates based on dynamic network visualization. 2. Preprocessing.R: (i) Removes metabolites with more than 60% of the values missing from the dataset; (ii) Replaces missing values with the metabolite's median at a given time point for the remaining dataset; (iii) Creates a data subset, containing only those metabolites, which are present at all time points. 3. BI.R: Function that calculates pBI scores for all metabolites. 4. InferBIGraph.R: Function to sum up the function calls for the calculation of a network graph. 5. FunctionsGraph.R: Multiple functions for network graph construction and visualization (i.e. create boxplot diagrams with different thresholds, graph calculation, adapt ratio for heatmap construction, plot heatmaps, plot graphs, calculate discrete weights, calculate degree-based weights, calculate graph object, plot pBI scores as bar charts). 6. *.txt files: Contain coordinates for graph visualization. For further information please read the “ReadMe.txt” file in the Supporting information. (ZIP) [file pone.0208953.s004.zip › S1_File/output/Example_Plots/02_Graphs/StatThresh/q50/Network_t240_q50stat.png]

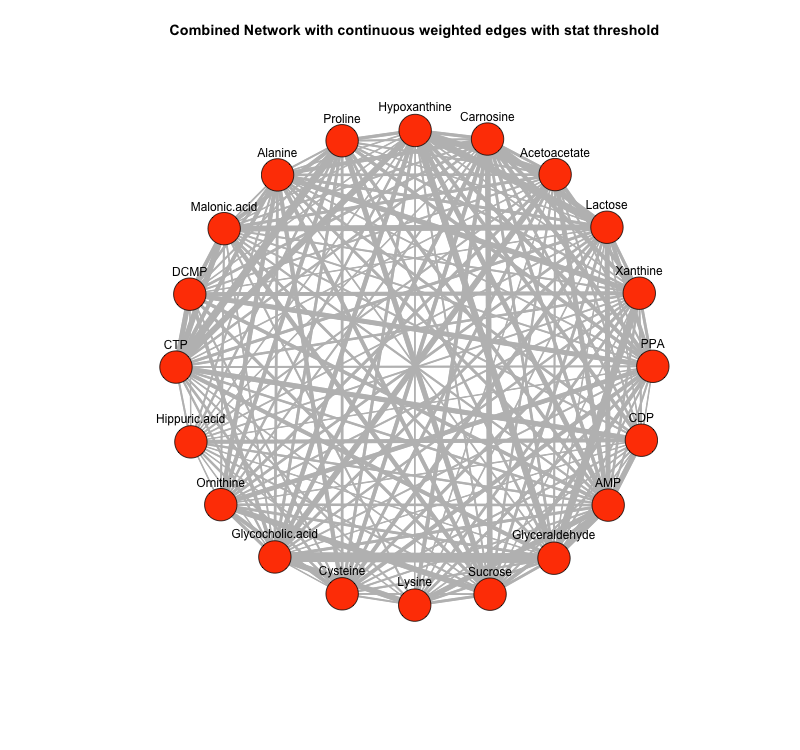

Supplement: S1 File — The R-based computational framework for data preprocessing, metabolite subset selection and dynamic network construction consists of the following R-scripts and text files: 1. Main.R: Main script for the analysis of metabolic data in order to identify putative biomarker candidates based on dynamic network visualization. 2. Preprocessing.R: (i) Removes metabolites with more than 60% of the values missing from the dataset; (ii) Replaces missing values with the metabolite's median at a given time point for the remaining dataset; (iii) Creates a data subset, containing only those metabolites, which are present at all time points. 3. BI.R: Function that calculates pBI scores for all metabolites. 4. InferBIGraph.R: Function to sum up the function calls for the calculation of a network graph. 5. FunctionsGraph.R: Multiple functions for network graph construction and visualization (i.e. create boxplot diagrams with different thresholds, graph calculation, adapt ratio for heatmap construction, plot heatmaps, plot graphs, calculate discrete weights, calculate degree-based weights, calculate graph object, plot pBI scores as bar charts). 6. *.txt files: Contain coordinates for graph visualization. For further information please read the “ReadMe.txt” file in the Supporting information. (ZIP) [file pone.0208953.s004.zip › S1_File/output/Example_Plots/02_Graphs/StatThresh/q50/quad/Network_continuous_weighted_4_graphs_q50stat.png]

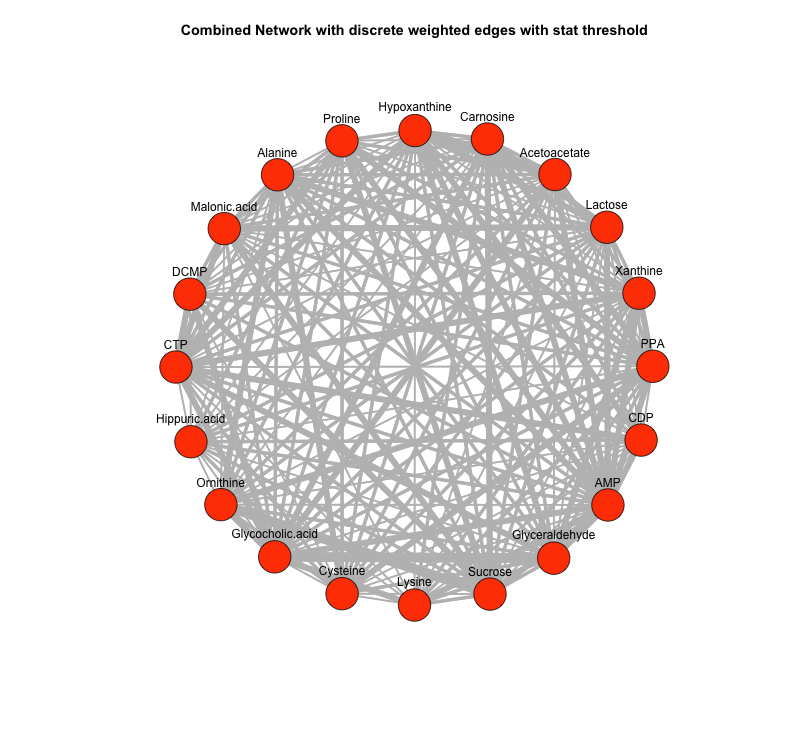

Supplement: S1 File — The R-based computational framework for data preprocessing, metabolite subset selection and dynamic network construction consists of the following R-scripts and text files: 1. Main.R: Main script for the analysis of metabolic data in order to identify putative biomarker candidates based on dynamic network visualization. 2. Preprocessing.R: (i) Removes metabolites with more than 60% of the values missing from the dataset; (ii) Replaces missing values with the metabolite's median at a given time point for the remaining dataset; (iii) Creates a data subset, containing only those metabolites, which are present at all time points. 3. BI.R: Function that calculates pBI scores for all metabolites. 4. InferBIGraph.R: Function to sum up the function calls for the calculation of a network graph. 5. FunctionsGraph.R: Multiple functions for network graph construction and visualization (i.e. create boxplot diagrams with different thresholds, graph calculation, adapt ratio for heatmap construction, plot heatmaps, plot graphs, calculate discrete weights, calculate degree-based weights, calculate graph object, plot pBI scores as bar charts). 6. *.txt files: Contain coordinates for graph visualization. For further information please read the “ReadMe.txt” file in the Supporting information. (ZIP) [file pone.0208953.s004.zip › S1_File/output/Example_Plots/02_Graphs/StatThresh/q50/quad/Network_discrete_weighted_4_graphs_q50stat.png]

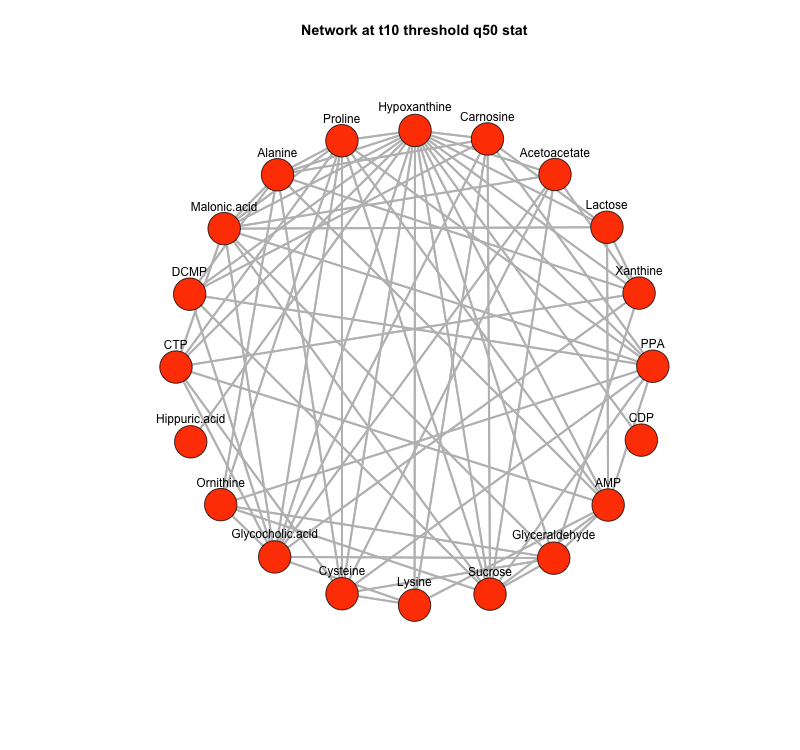

Supplement: S1 File — The R-based computational framework for data preprocessing, metabolite subset selection and dynamic network construction consists of the following R-scripts and text files: 1. Main.R: Main script for the analysis of metabolic data in order to identify putative biomarker candidates based on dynamic network visualization. 2. Preprocessing.R: (i) Removes metabolites with more than 60% of the values missing from the dataset; (ii) Replaces missing values with the metabolite's median at a given time point for the remaining dataset; (iii) Creates a data subset, containing only those metabolites, which are present at all time points. 3. BI.R: Function that calculates pBI scores for all metabolites. 4. InferBIGraph.R: Function to sum up the function calls for the calculation of a network graph. 5. FunctionsGraph.R: Multiple functions for network graph construction and visualization (i.e. create boxplot diagrams with different thresholds, graph calculation, adapt ratio for heatmap construction, plot heatmaps, plot graphs, calculate discrete weights, calculate degree-based weights, calculate graph object, plot pBI scores as bar charts). 6. *.txt files: Contain coordinates for graph visualization. For further information please read the “ReadMe.txt” file in the Supporting information. (ZIP) [file pone.0208953.s004.zip › S1_File/output/Example_Plots/02_Graphs/StatThresh/q50/Network_t10_q50stat.png]

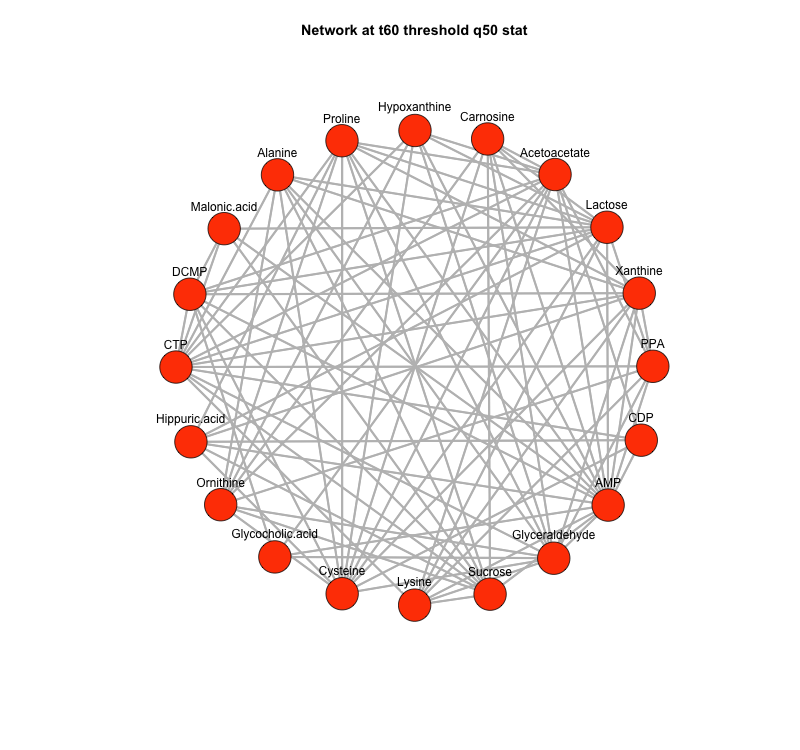

Supplement: S1 File — The R-based computational framework for data preprocessing, metabolite subset selection and dynamic network construction consists of the following R-scripts and text files: 1. Main.R: Main script for the analysis of metabolic data in order to identify putative biomarker candidates based on dynamic network visualization. 2. Preprocessing.R: (i) Removes metabolites with more than 60% of the values missing from the dataset; (ii) Replaces missing values with the metabolite's median at a given time point for the remaining dataset; (iii) Creates a data subset, containing only those metabolites, which are present at all time points. 3. BI.R: Function that calculates pBI scores for all metabolites. 4. InferBIGraph.R: Function to sum up the function calls for the calculation of a network graph. 5. FunctionsGraph.R: Multiple functions for network graph construction and visualization (i.e. create boxplot diagrams with different thresholds, graph calculation, adapt ratio for heatmap construction, plot heatmaps, plot graphs, calculate discrete weights, calculate degree-based weights, calculate graph object, plot pBI scores as bar charts). 6. *.txt files: Contain coordinates for graph visualization. For further information please read the “ReadMe.txt” file in the Supporting information. (ZIP) [file pone.0208953.s004.zip › S1_File/output/Example_Plots/02_Graphs/StatThresh/q50/Network_t60_q50stat.png]

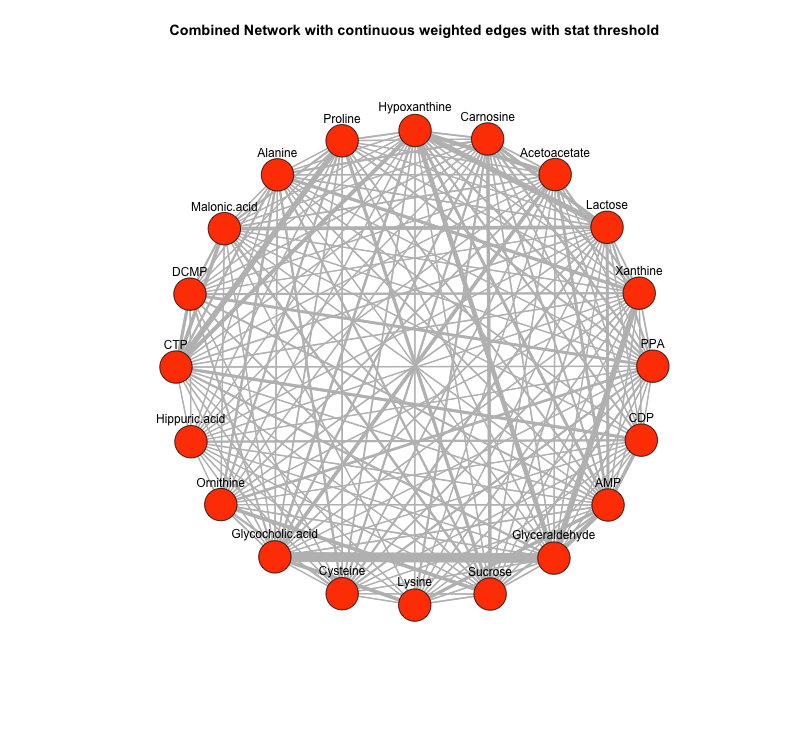

Supplement: S1 File — The R-based computational framework for data preprocessing, metabolite subset selection and dynamic network construction consists of the following R-scripts and text files: 1. Main.R: Main script for the analysis of metabolic data in order to identify putative biomarker candidates based on dynamic network visualization. 2. Preprocessing.R: (i) Removes metabolites with more than 60% of the values missing from the dataset; (ii) Replaces missing values with the metabolite's median at a given time point for the remaining dataset; (iii) Creates a data subset, containing only those metabolites, which are present at all time points. 3. BI.R: Function that calculates pBI scores for all metabolites. 4. InferBIGraph.R: Function to sum up the function calls for the calculation of a network graph. 5. FunctionsGraph.R: Multiple functions for network graph construction and visualization (i.e. create boxplot diagrams with different thresholds, graph calculation, adapt ratio for heatmap construction, plot heatmaps, plot graphs, calculate discrete weights, calculate degree-based weights, calculate graph object, plot pBI scores as bar charts). 6. *.txt files: Contain coordinates for graph visualization. For further information please read the “ReadMe.txt” file in the Supporting information. (ZIP) [file pone.0208953.s004.zip › S1_File/output/Example_Plots/02_Graphs/StatThresh/q50/poly4/Network_continuous_weighted_4_graphs_q50stat.png]

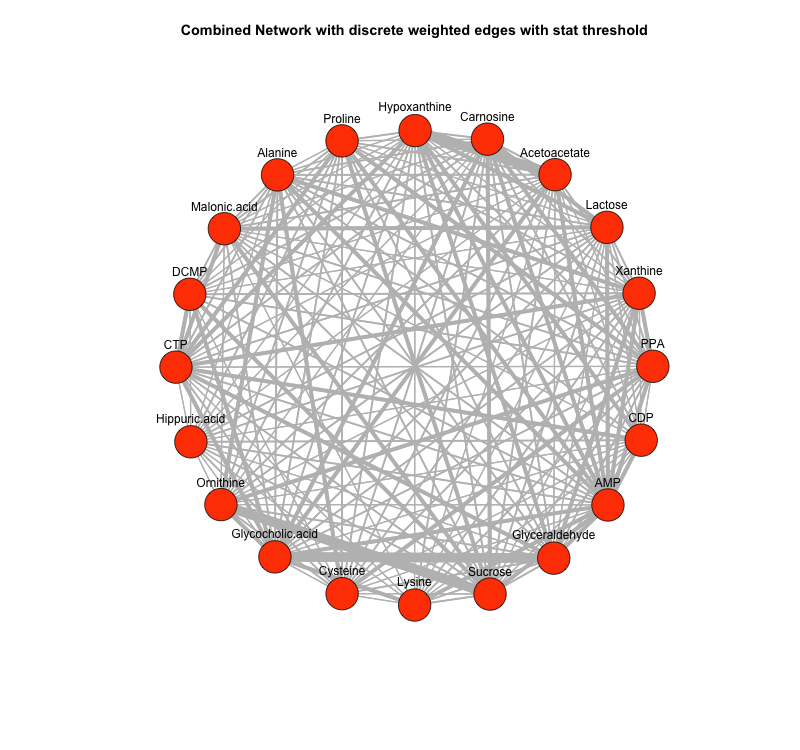

Supplement: S1 File — The R-based computational framework for data preprocessing, metabolite subset selection and dynamic network construction consists of the following R-scripts and text files: 1. Main.R: Main script for the analysis of metabolic data in order to identify putative biomarker candidates based on dynamic network visualization. 2. Preprocessing.R: (i) Removes metabolites with more than 60% of the values missing from the dataset; (ii) Replaces missing values with the metabolite's median at a given time point for the remaining dataset; (iii) Creates a data subset, containing only those metabolites, which are present at all time points. 3. BI.R: Function that calculates pBI scores for all metabolites. 4. InferBIGraph.R: Function to sum up the function calls for the calculation of a network graph. 5. FunctionsGraph.R: Multiple functions for network graph construction and visualization (i.e. create boxplot diagrams with different thresholds, graph calculation, adapt ratio for heatmap construction, plot heatmaps, plot graphs, calculate discrete weights, calculate degree-based weights, calculate graph object, plot pBI scores as bar charts). 6. *.txt files: Contain coordinates for graph visualization. For further information please read the “ReadMe.txt” file in the Supporting information. (ZIP) [file pone.0208953.s004.zip › S1_File/output/Example_Plots/02_Graphs/StatThresh/q50/poly4/Network_discrete_weighted_4_graphs_q50stat.png]

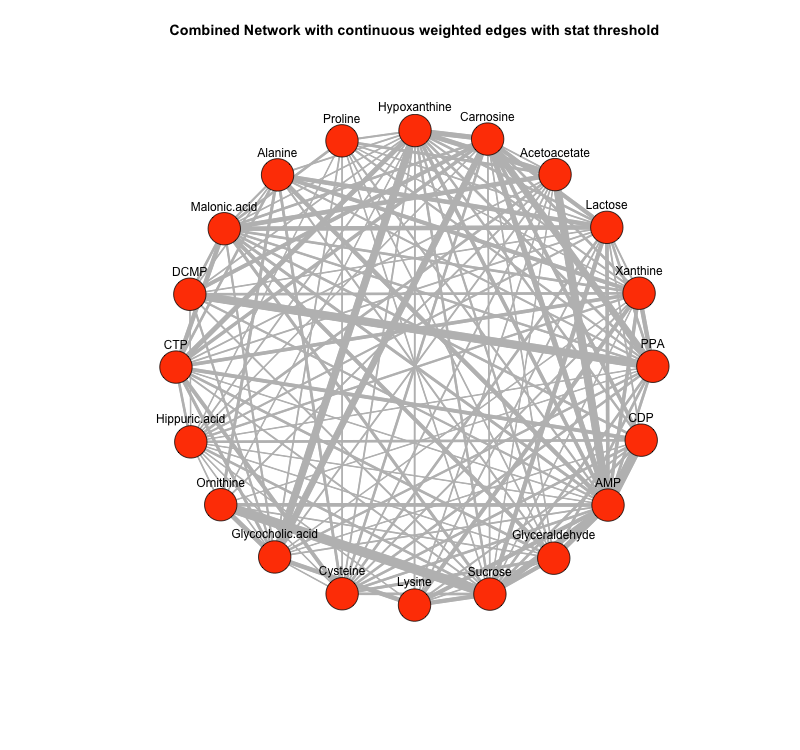

Supplement: S1 File — The R-based computational framework for data preprocessing, metabolite subset selection and dynamic network construction consists of the following R-scripts and text files: 1. Main.R: Main script for the analysis of metabolic data in order to identify putative biomarker candidates based on dynamic network visualization. 2. Preprocessing.R: (i) Removes metabolites with more than 60% of the values missing from the dataset; (ii) Replaces missing values with the metabolite's median at a given time point for the remaining dataset; (iii) Creates a data subset, containing only those metabolites, which are present at all time points. 3. BI.R: Function that calculates pBI scores for all metabolites. 4. InferBIGraph.R: Function to sum up the function calls for the calculation of a network graph. 5. FunctionsGraph.R: Multiple functions for network graph construction and visualization (i.e. create boxplot diagrams with different thresholds, graph calculation, adapt ratio for heatmap construction, plot heatmaps, plot graphs, calculate discrete weights, calculate degree-based weights, calculate graph object, plot pBI scores as bar charts). 6. *.txt files: Contain coordinates for graph visualization. For further information please read the “ReadMe.txt” file in the Supporting information. (ZIP) [file pone.0208953.s004.zip › S1_File/output/Example_Plots/02_Graphs/StatThresh/q75/quad/Network_continuous_weighted_4_graphs_q75stat.png]

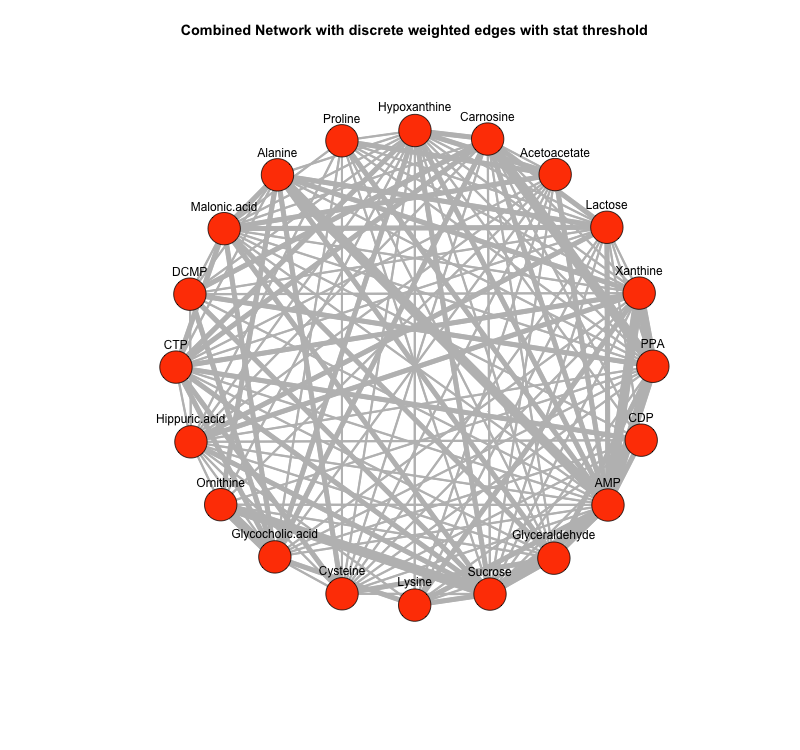

Supplement: S1 File — The R-based computational framework for data preprocessing, metabolite subset selection and dynamic network construction consists of the following R-scripts and text files: 1. Main.R: Main script for the analysis of metabolic data in order to identify putative biomarker candidates based on dynamic network visualization. 2. Preprocessing.R: (i) Removes metabolites with more than 60% of the values missing from the dataset; (ii) Replaces missing values with the metabolite's median at a given time point for the remaining dataset; (iii) Creates a data subset, containing only those metabolites, which are present at all time points. 3. BI.R: Function that calculates pBI scores for all metabolites. 4. InferBIGraph.R: Function to sum up the function calls for the calculation of a network graph. 5. FunctionsGraph.R: Multiple functions for network graph construction and visualization (i.e. create boxplot diagrams with different thresholds, graph calculation, adapt ratio for heatmap construction, plot heatmaps, plot graphs, calculate discrete weights, calculate degree-based weights, calculate graph object, plot pBI scores as bar charts). 6. *.txt files: Contain coordinates for graph visualization. For further information please read the “ReadMe.txt” file in the Supporting information. (ZIP) [file pone.0208953.s004.zip › S1_File/output/Example_Plots/02_Graphs/StatThresh/q75/quad/Network_discrete_weighted_4_graphs_q75stat.png]

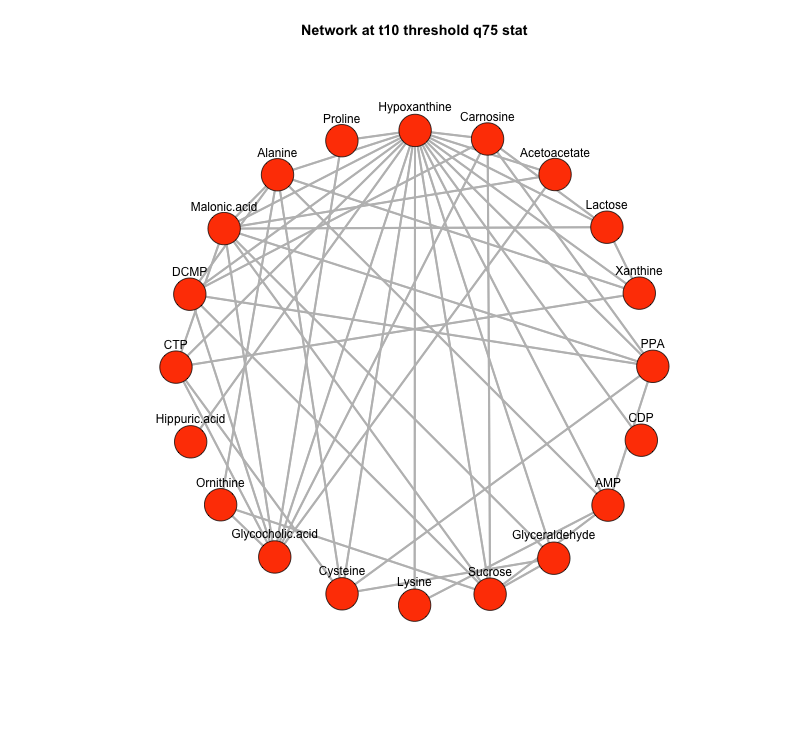

Supplement: S1 File — The R-based computational framework for data preprocessing, metabolite subset selection and dynamic network construction consists of the following R-scripts and text files: 1. Main.R: Main script for the analysis of metabolic data in order to identify putative biomarker candidates based on dynamic network visualization. 2. Preprocessing.R: (i) Removes metabolites with more than 60% of the values missing from the dataset; (ii) Replaces missing values with the metabolite's median at a given time point for the remaining dataset; (iii) Creates a data subset, containing only those metabolites, which are present at all time points. 3. BI.R: Function that calculates pBI scores for all metabolites. 4. InferBIGraph.R: Function to sum up the function calls for the calculation of a network graph. 5. FunctionsGraph.R: Multiple functions for network graph construction and visualization (i.e. create boxplot diagrams with different thresholds, graph calculation, adapt ratio for heatmap construction, plot heatmaps, plot graphs, calculate discrete weights, calculate degree-based weights, calculate graph object, plot pBI scores as bar charts). 6. *.txt files: Contain coordinates for graph visualization. For further information please read the “ReadMe.txt” file in the Supporting information. (ZIP) [file pone.0208953.s004.zip › S1_File/output/Example_Plots/02_Graphs/StatThresh/q75/Network_t10_q75stat.png]

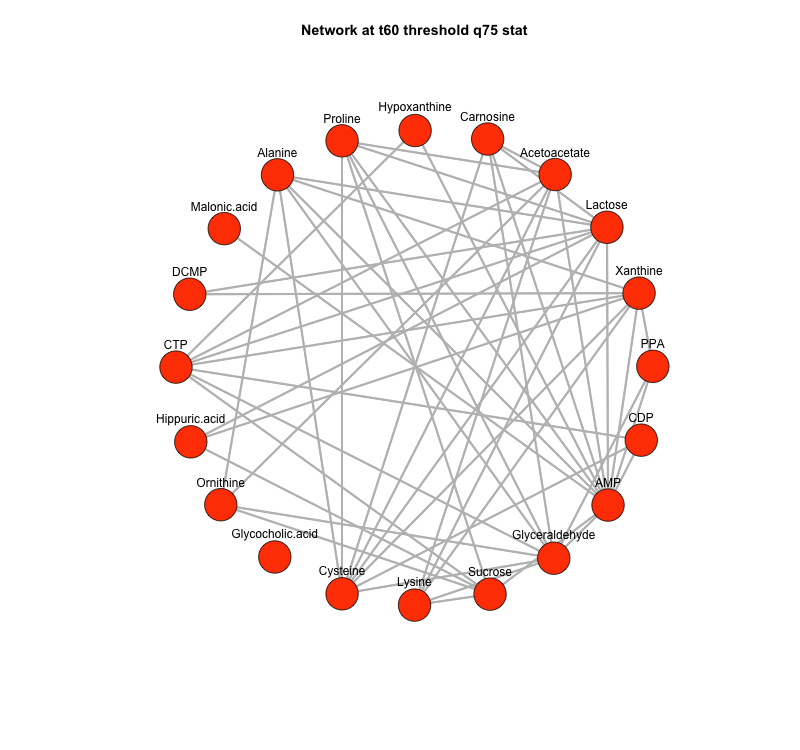

Supplement: S1 File — The R-based computational framework for data preprocessing, metabolite subset selection and dynamic network construction consists of the following R-scripts and text files: 1. Main.R: Main script for the analysis of metabolic data in order to identify putative biomarker candidates based on dynamic network visualization. 2. Preprocessing.R: (i) Removes metabolites with more than 60% of the values missing from the dataset; (ii) Replaces missing values with the metabolite's median at a given time point for the remaining dataset; (iii) Creates a data subset, containing only those metabolites, which are present at all time points. 3. BI.R: Function that calculates pBI scores for all metabolites. 4. InferBIGraph.R: Function to sum up the function calls for the calculation of a network graph. 5. FunctionsGraph.R: Multiple functions for network graph construction and visualization (i.e. create boxplot diagrams with different thresholds, graph calculation, adapt ratio for heatmap construction, plot heatmaps, plot graphs, calculate discrete weights, calculate degree-based weights, calculate graph object, plot pBI scores as bar charts). 6. *.txt files: Contain coordinates for graph visualization. For further information please read the “ReadMe.txt” file in the Supporting information. (ZIP) [file pone.0208953.s004.zip › S1_File/output/Example_Plots/02_Graphs/StatThresh/q75/Network_t60_q75stat.png]

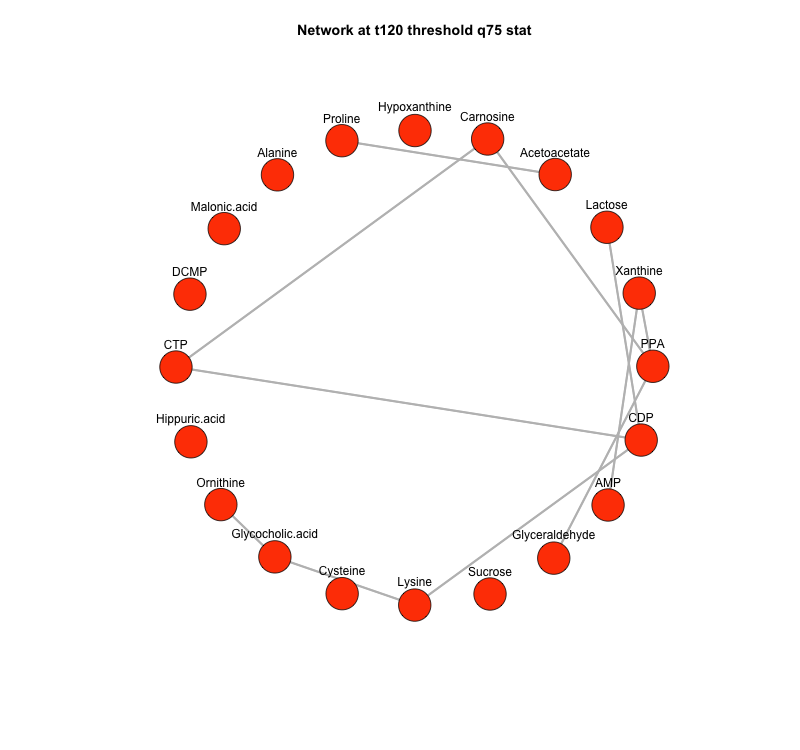

Supplement: S1 File — The R-based computational framework for data preprocessing, metabolite subset selection and dynamic network construction consists of the following R-scripts and text files: 1. Main.R: Main script for the analysis of metabolic data in order to identify putative biomarker candidates based on dynamic network visualization. 2. Preprocessing.R: (i) Removes metabolites with more than 60% of the values missing from the dataset; (ii) Replaces missing values with the metabolite's median at a given time point for the remaining dataset; (iii) Creates a data subset, containing only those metabolites, which are present at all time points. 3. BI.R: Function that calculates pBI scores for all metabolites. 4. InferBIGraph.R: Function to sum up the function calls for the calculation of a network graph. 5. FunctionsGraph.R: Multiple functions for network graph construction and visualization (i.e. create boxplot diagrams with different thresholds, graph calculation, adapt ratio for heatmap construction, plot heatmaps, plot graphs, calculate discrete weights, calculate degree-based weights, calculate graph object, plot pBI scores as bar charts). 6. *.txt files: Contain coordinates for graph visualization. For further information please read the “ReadMe.txt” file in the Supporting information. (ZIP) [file pone.0208953.s004.zip › S1_File/output/Example_Plots/02_Graphs/StatThresh/q75/Network_t120_q75stat.png]

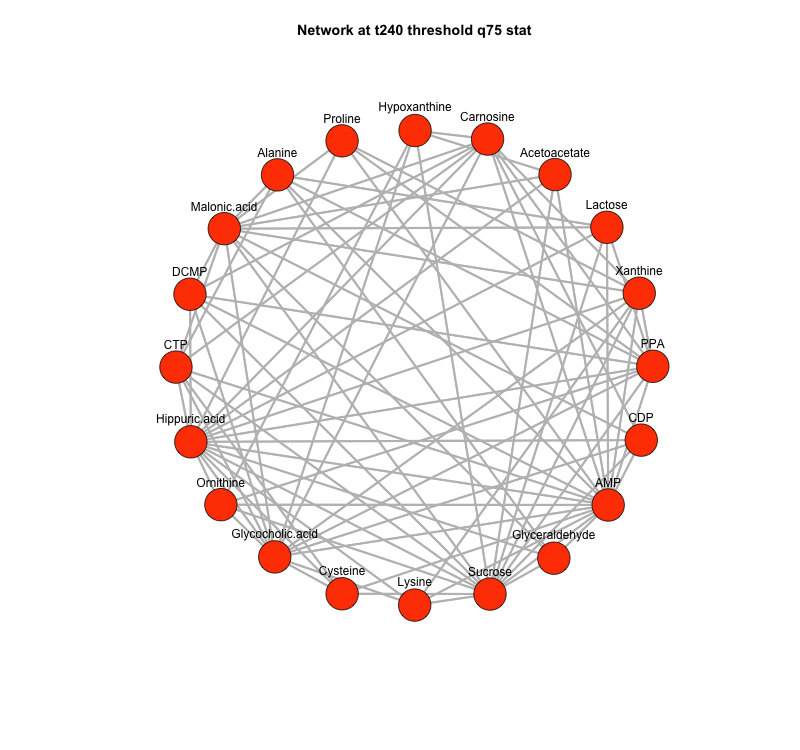

Supplement: S1 File — The R-based computational framework for data preprocessing, metabolite subset selection and dynamic network construction consists of the following R-scripts and text files: 1. Main.R: Main script for the analysis of metabolic data in order to identify putative biomarker candidates based on dynamic network visualization. 2. Preprocessing.R: (i) Removes metabolites with more than 60% of the values missing from the dataset; (ii) Replaces missing values with the metabolite's median at a given time point for the remaining dataset; (iii) Creates a data subset, containing only those metabolites, which are present at all time points. 3. BI.R: Function that calculates pBI scores for all metabolites. 4. InferBIGraph.R: Function to sum up the function calls for the calculation of a network graph. 5. FunctionsGraph.R: Multiple functions for network graph construction and visualization (i.e. create boxplot diagrams with different thresholds, graph calculation, adapt ratio for heatmap construction, plot heatmaps, plot graphs, calculate discrete weights, calculate degree-based weights, calculate graph object, plot pBI scores as bar charts). 6. *.txt files: Contain coordinates for graph visualization. For further information please read the “ReadMe.txt” file in the Supporting information. (ZIP) [file pone.0208953.s004.zip › S1_File/output/Example_Plots/02_Graphs/StatThresh/q75/Network_t240_q75stat.png]

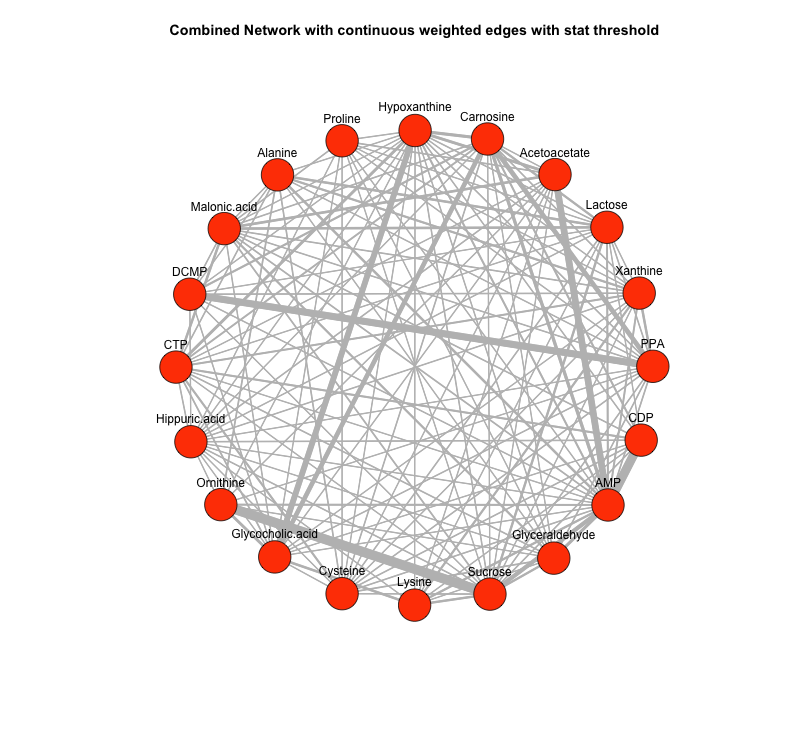

Supplement: S1 File — The R-based computational framework for data preprocessing, metabolite subset selection and dynamic network construction consists of the following R-scripts and text files: 1. Main.R: Main script for the analysis of metabolic data in order to identify putative biomarker candidates based on dynamic network visualization. 2. Preprocessing.R: (i) Removes metabolites with more than 60% of the values missing from the dataset; (ii) Replaces missing values with the metabolite's median at a given time point for the remaining dataset; (iii) Creates a data subset, containing only those metabolites, which are present at all time points. 3. BI.R: Function that calculates pBI scores for all metabolites. 4. InferBIGraph.R: Function to sum up the function calls for the calculation of a network graph. 5. FunctionsGraph.R: Multiple functions for network graph construction and visualization (i.e. create boxplot diagrams with different thresholds, graph calculation, adapt ratio for heatmap construction, plot heatmaps, plot graphs, calculate discrete weights, calculate degree-based weights, calculate graph object, plot pBI scores as bar charts). 6. *.txt files: Contain coordinates for graph visualization. For further information please read the “ReadMe.txt” file in the Supporting information. (ZIP) [file pone.0208953.s004.zip › S1_File/output/Example_Plots/02_Graphs/StatThresh/q75/poly4/Network_continuous_weighted_4_graphs_q75stat.png]

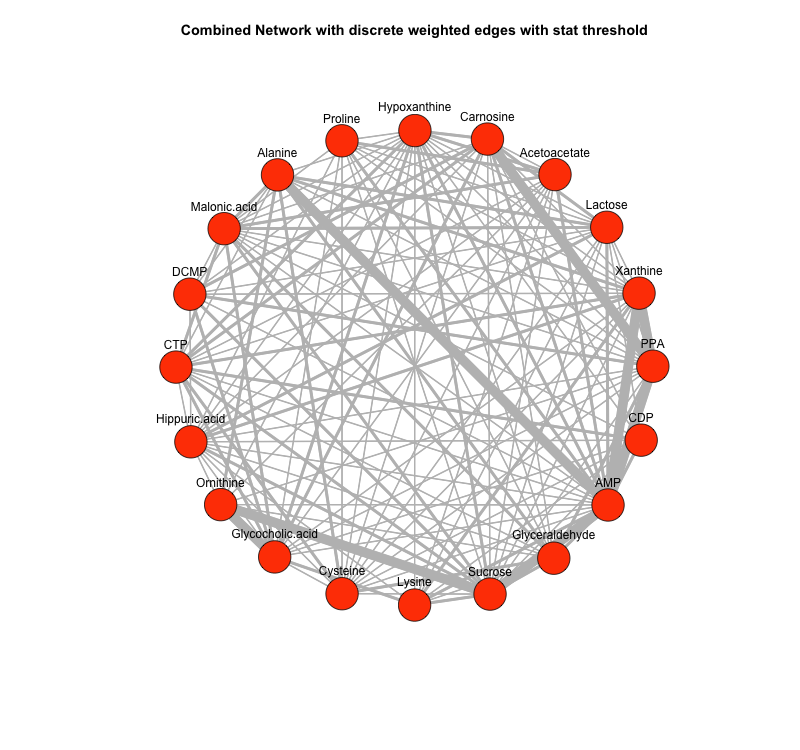

Supplement: S1 File — The R-based computational framework for data preprocessing, metabolite subset selection and dynamic network construction consists of the following R-scripts and text files: 1. Main.R: Main script for the analysis of metabolic data in order to identify putative biomarker candidates based on dynamic network visualization. 2. Preprocessing.R: (i) Removes metabolites with more than 60% of the values missing from the dataset; (ii) Replaces missing values with the metabolite's median at a given time point for the remaining dataset; (iii) Creates a data subset, containing only those metabolites, which are present at all time points. 3. BI.R: Function that calculates pBI scores for all metabolites. 4. InferBIGraph.R: Function to sum up the function calls for the calculation of a network graph. 5. FunctionsGraph.R: Multiple functions for network graph construction and visualization (i.e. create boxplot diagrams with different thresholds, graph calculation, adapt ratio for heatmap construction, plot heatmaps, plot graphs, calculate discrete weights, calculate degree-based weights, calculate graph object, plot pBI scores as bar charts). 6. *.txt files: Contain coordinates for graph visualization. For further information please read the “ReadMe.txt” file in the Supporting information. (ZIP) [file pone.0208953.s004.zip › S1_File/output/Example_Plots/02_Graphs/StatThresh/q75/poly4/Network_discrete_weighted_4_graphs_q75stat.png]

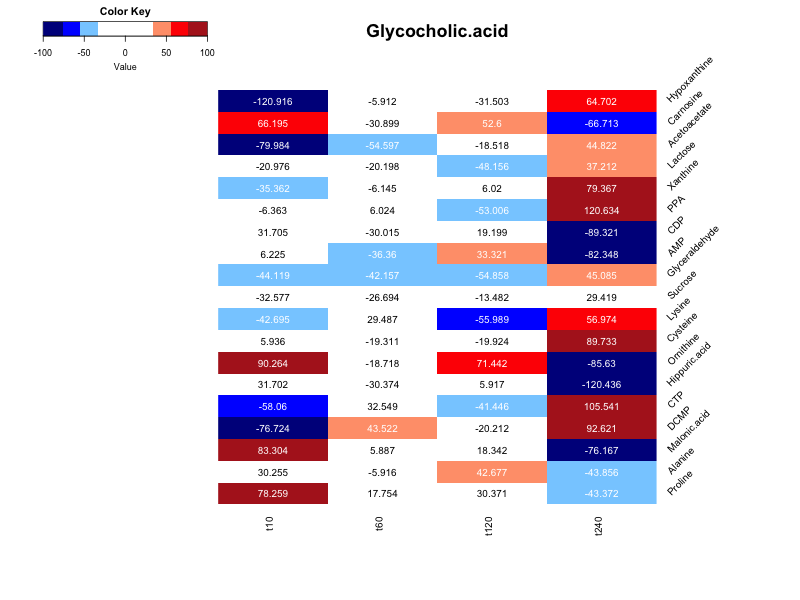

Supplement: S1 File — The R-based computational framework for data preprocessing, metabolite subset selection and dynamic network construction consists of the following R-scripts and text files: 1. Main.R: Main script for the analysis of metabolic data in order to identify putative biomarker candidates based on dynamic network visualization. 2. Preprocessing.R: (i) Removes metabolites with more than 60% of the values missing from the dataset; (ii) Replaces missing values with the metabolite's median at a given time point for the remaining dataset; (iii) Creates a data subset, containing only those metabolites, which are present at all time points. 3. BI.R: Function that calculates pBI scores for all metabolites. 4. InferBIGraph.R: Function to sum up the function calls for the calculation of a network graph. 5. FunctionsGraph.R: Multiple functions for network graph construction and visualization (i.e. create boxplot diagrams with different thresholds, graph calculation, adapt ratio for heatmap construction, plot heatmaps, plot graphs, calculate discrete weights, calculate degree-based weights, calculate graph object, plot pBI scores as bar charts). 6. *.txt files: Contain coordinates for graph visualization. For further information please read the “ReadMe.txt” file in the Supporting information. (ZIP) [file pone.0208953.s004.zip › S1_File/output/Example_Plots/03_Heatmaps/Metabolite Glycocholic.acid.png]

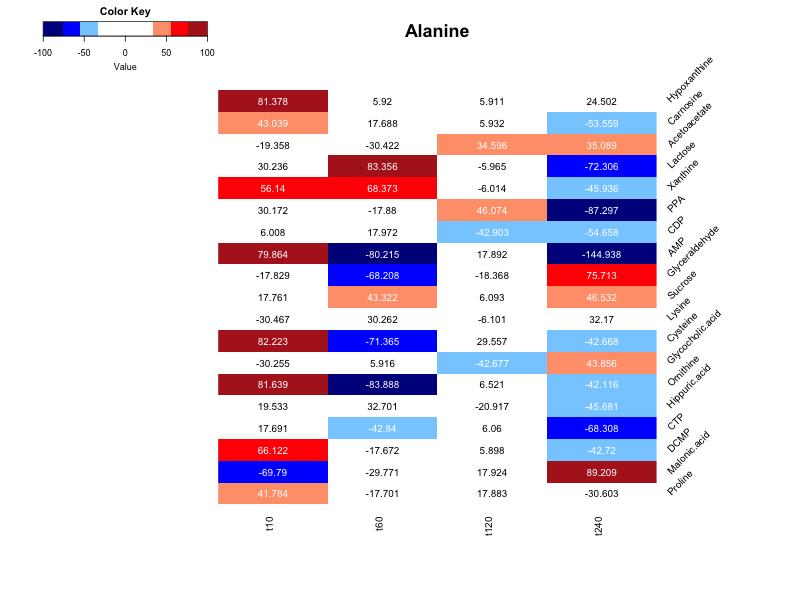

Supplement: S1 File — The R-based computational framework for data preprocessing, metabolite subset selection and dynamic network construction consists of the following R-scripts and text files: 1. Main.R: Main script for the analysis of metabolic data in order to identify putative biomarker candidates based on dynamic network visualization. 2. Preprocessing.R: (i) Removes metabolites with more than 60% of the values missing from the dataset; (ii) Replaces missing values with the metabolite's median at a given time point for the remaining dataset; (iii) Creates a data subset, containing only those metabolites, which are present at all time points. 3. BI.R: Function that calculates pBI scores for all metabolites. 4. InferBIGraph.R: Function to sum up the function calls for the calculation of a network graph. 5. FunctionsGraph.R: Multiple functions for network graph construction and visualization (i.e. create boxplot diagrams with different thresholds, graph calculation, adapt ratio for heatmap construction, plot heatmaps, plot graphs, calculate discrete weights, calculate degree-based weights, calculate graph object, plot pBI scores as bar charts). 6. *.txt files: Contain coordinates for graph visualization. For further information please read the “ReadMe.txt” file in the Supporting information. (ZIP) [file pone.0208953.s004.zip › S1_File/output/Example_Plots/03_Heatmaps/Metabolite Alanine.png]

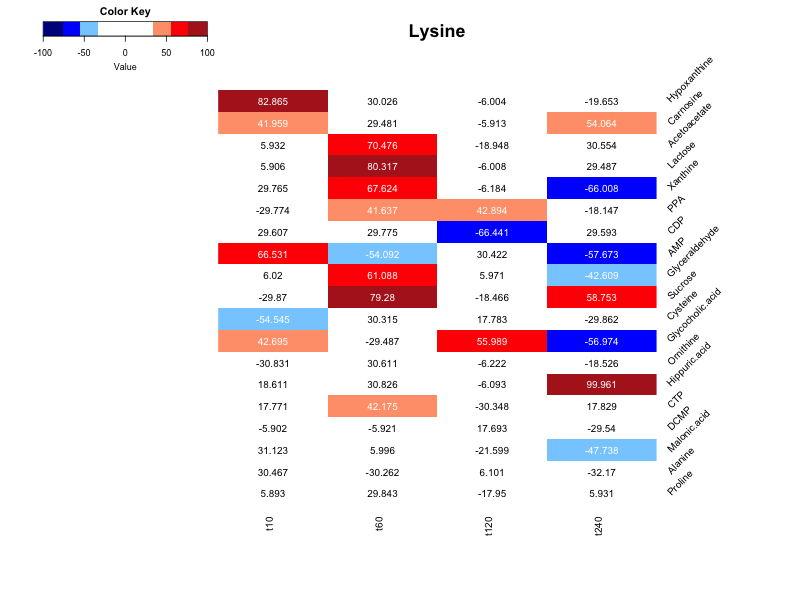

Supplement: S1 File — The R-based computational framework for data preprocessing, metabolite subset selection and dynamic network construction consists of the following R-scripts and text files: 1. Main.R: Main script for the analysis of metabolic data in order to identify putative biomarker candidates based on dynamic network visualization. 2. Preprocessing.R: (i) Removes metabolites with more than 60% of the values missing from the dataset; (ii) Replaces missing values with the metabolite's median at a given time point for the remaining dataset; (iii) Creates a data subset, containing only those metabolites, which are present at all time points. 3. BI.R: Function that calculates pBI scores for all metabolites. 4. InferBIGraph.R: Function to sum up the function calls for the calculation of a network graph. 5. FunctionsGraph.R: Multiple functions for network graph construction and visualization (i.e. create boxplot diagrams with different thresholds, graph calculation, adapt ratio for heatmap construction, plot heatmaps, plot graphs, calculate discrete weights, calculate degree-based weights, calculate graph object, plot pBI scores as bar charts). 6. *.txt files: Contain coordinates for graph visualization. For further information please read the “ReadMe.txt” file in the Supporting information. (ZIP) [file pone.0208953.s004.zip › S1_File/output/Example_Plots/03_Heatmaps/Metabolite Lysine.png]

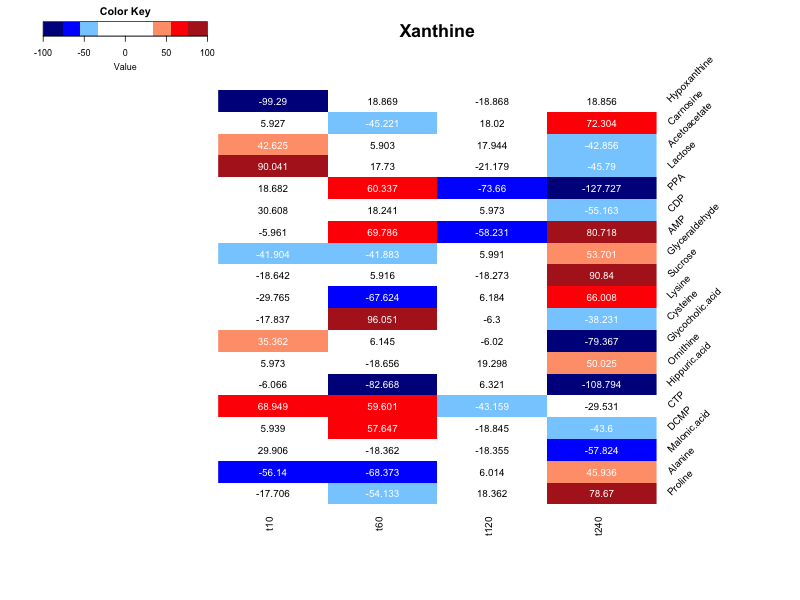

Supplement: S1 File — The R-based computational framework for data preprocessing, metabolite subset selection and dynamic network construction consists of the following R-scripts and text files: 1. Main.R: Main script for the analysis of metabolic data in order to identify putative biomarker candidates based on dynamic network visualization. 2. Preprocessing.R: (i) Removes metabolites with more than 60% of the values missing from the dataset; (ii) Replaces missing values with the metabolite's median at a given time point for the remaining dataset; (iii) Creates a data subset, containing only those metabolites, which are present at all time points. 3. BI.R: Function that calculates pBI scores for all metabolites. 4. InferBIGraph.R: Function to sum up the function calls for the calculation of a network graph. 5. FunctionsGraph.R: Multiple functions for network graph construction and visualization (i.e. create boxplot diagrams with different thresholds, graph calculation, adapt ratio for heatmap construction, plot heatmaps, plot graphs, calculate discrete weights, calculate degree-based weights, calculate graph object, plot pBI scores as bar charts). 6. *.txt files: Contain coordinates for graph visualization. For further information please read the “ReadMe.txt” file in the Supporting information. (ZIP) [file pone.0208953.s004.zip › S1_File/output/Example_Plots/03_Heatmaps/Metabolite Xanthine.png]

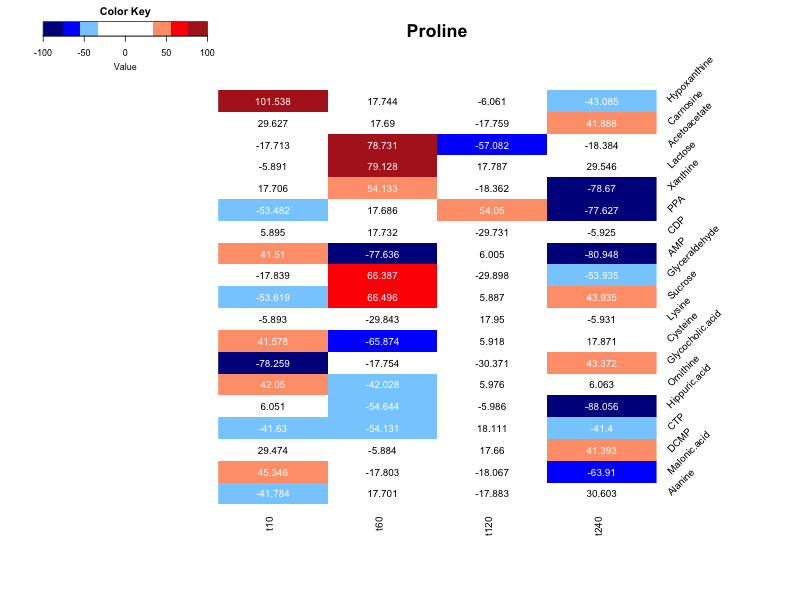

Supplement: S1 File — The R-based computational framework for data preprocessing, metabolite subset selection and dynamic network construction consists of the following R-scripts and text files: 1. Main.R: Main script for the analysis of metabolic data in order to identify putative biomarker candidates based on dynamic network visualization. 2. Preprocessing.R: (i) Removes metabolites with more than 60% of the values missing from the dataset; (ii) Replaces missing values with the metabolite's median at a given time point for the remaining dataset; (iii) Creates a data subset, containing only those metabolites, which are present at all time points. 3. BI.R: Function that calculates pBI scores for all metabolites. 4. InferBIGraph.R: Function to sum up the function calls for the calculation of a network graph. 5. FunctionsGraph.R: Multiple functions for network graph construction and visualization (i.e. create boxplot diagrams with different thresholds, graph calculation, adapt ratio for heatmap construction, plot heatmaps, plot graphs, calculate discrete weights, calculate degree-based weights, calculate graph object, plot pBI scores as bar charts). 6. *.txt files: Contain coordinates for graph visualization. For further information please read the “ReadMe.txt” file in the Supporting information. (ZIP) [file pone.0208953.s004.zip › S1_File/output/Example_Plots/03_Heatmaps/Metabolite Proline.png]

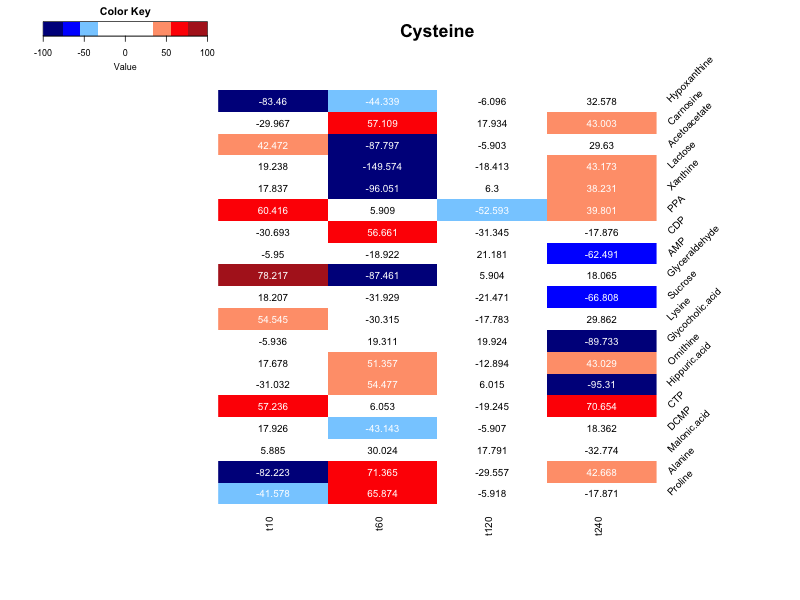

Supplement: S1 File — The R-based computational framework for data preprocessing, metabolite subset selection and dynamic network construction consists of the following R-scripts and text files: 1. Main.R: Main script for the analysis of metabolic data in order to identify putative biomarker candidates based on dynamic network visualization. 2. Preprocessing.R: (i) Removes metabolites with more than 60% of the values missing from the dataset; (ii) Replaces missing values with the metabolite's median at a given time point for the remaining dataset; (iii) Creates a data subset, containing only those metabolites, which are present at all time points. 3. BI.R: Function that calculates pBI scores for all metabolites. 4. InferBIGraph.R: Function to sum up the function calls for the calculation of a network graph. 5. FunctionsGraph.R: Multiple functions for network graph construction and visualization (i.e. create boxplot diagrams with different thresholds, graph calculation, adapt ratio for heatmap construction, plot heatmaps, plot graphs, calculate discrete weights, calculate degree-based weights, calculate graph object, plot pBI scores as bar charts). 6. *.txt files: Contain coordinates for graph visualization. For further information please read the “ReadMe.txt” file in the Supporting information. (ZIP) [file pone.0208953.s004.zip › S1_File/output/Example_Plots/03_Heatmaps/Metabolite Cysteine.png]

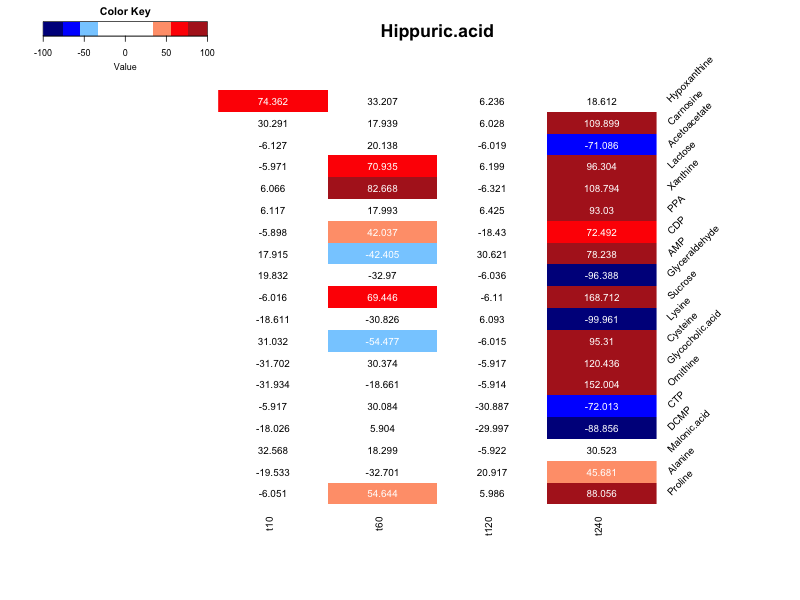

Supplement: S1 File — The R-based computational framework for data preprocessing, metabolite subset selection and dynamic network construction consists of the following R-scripts and text files: 1. Main.R: Main script for the analysis of metabolic data in order to identify putative biomarker candidates based on dynamic network visualization. 2. Preprocessing.R: (i) Removes metabolites with more than 60% of the values missing from the dataset; (ii) Replaces missing values with the metabolite's median at a given time point for the remaining dataset; (iii) Creates a data subset, containing only those metabolites, which are present at all time points. 3. BI.R: Function that calculates pBI scores for all metabolites. 4. InferBIGraph.R: Function to sum up the function calls for the calculation of a network graph. 5. FunctionsGraph.R: Multiple functions for network graph construction and visualization (i.e. create boxplot diagrams with different thresholds, graph calculation, adapt ratio for heatmap construction, plot heatmaps, plot graphs, calculate discrete weights, calculate degree-based weights, calculate graph object, plot pBI scores as bar charts). 6. *.txt files: Contain coordinates for graph visualization. For further information please read the “ReadMe.txt” file in the Supporting information. (ZIP) [file pone.0208953.s004.zip › S1_File/output/Example_Plots/03_Heatmaps/Metabolite Hippuric.acid.png]

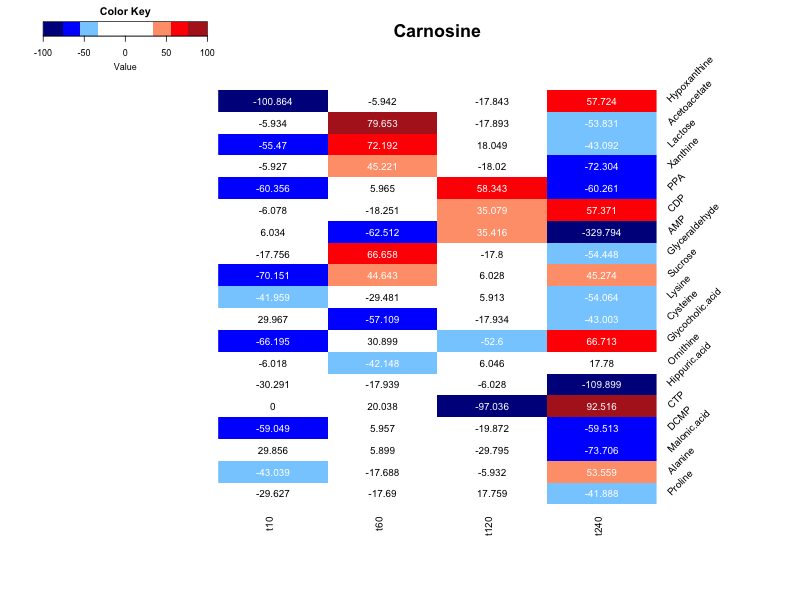

Supplement: S1 File — The R-based computational framework for data preprocessing, metabolite subset selection and dynamic network construction consists of the following R-scripts and text files: 1. Main.R: Main script for the analysis of metabolic data in order to identify putative biomarker candidates based on dynamic network visualization. 2. Preprocessing.R: (i) Removes metabolites with more than 60% of the values missing from the dataset; (ii) Replaces missing values with the metabolite's median at a given time point for the remaining dataset; (iii) Creates a data subset, containing only those metabolites, which are present at all time points. 3. BI.R: Function that calculates pBI scores for all metabolites. 4. InferBIGraph.R: Function to sum up the function calls for the calculation of a network graph. 5. FunctionsGraph.R: Multiple functions for network graph construction and visualization (i.e. create boxplot diagrams with different thresholds, graph calculation, adapt ratio for heatmap construction, plot heatmaps, plot graphs, calculate discrete weights, calculate degree-based weights, calculate graph object, plot pBI scores as bar charts). 6. *.txt files: Contain coordinates for graph visualization. For further information please read the “ReadMe.txt” file in the Supporting information. (ZIP) [file pone.0208953.s004.zip › S1_File/output/Example_Plots/03_Heatmaps/Metabolite Carnosine.png]

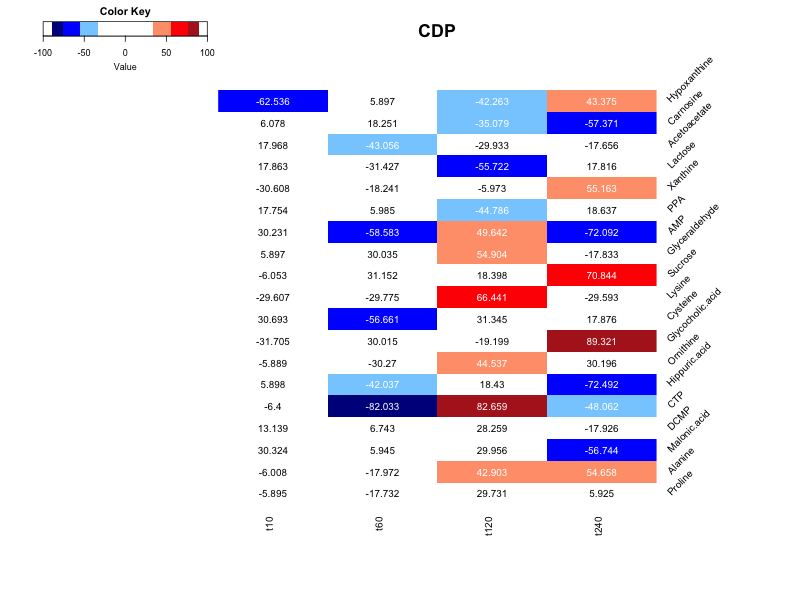

Supplement: S1 File — The R-based computational framework for data preprocessing, metabolite subset selection and dynamic network construction consists of the following R-scripts and text files: 1. Main.R: Main script for the analysis of metabolic data in order to identify putative biomarker candidates based on dynamic network visualization. 2. Preprocessing.R: (i) Removes metabolites with more than 60% of the values missing from the dataset; (ii) Replaces missing values with the metabolite's median at a given time point for the remaining dataset; (iii) Creates a data subset, containing only those metabolites, which are present at all time points. 3. BI.R: Function that calculates pBI scores for all metabolites. 4. InferBIGraph.R: Function to sum up the function calls for the calculation of a network graph. 5. FunctionsGraph.R: Multiple functions for network graph construction and visualization (i.e. create boxplot diagrams with different thresholds, graph calculation, adapt ratio for heatmap construction, plot heatmaps, plot graphs, calculate discrete weights, calculate degree-based weights, calculate graph object, plot pBI scores as bar charts). 6. *.txt files: Contain coordinates for graph visualization. For further information please read the “ReadMe.txt” file in the Supporting information. (ZIP) [file pone.0208953.s004.zip › S1_File/output/Example_Plots/03_Heatmaps/Metabolite CDP.png]

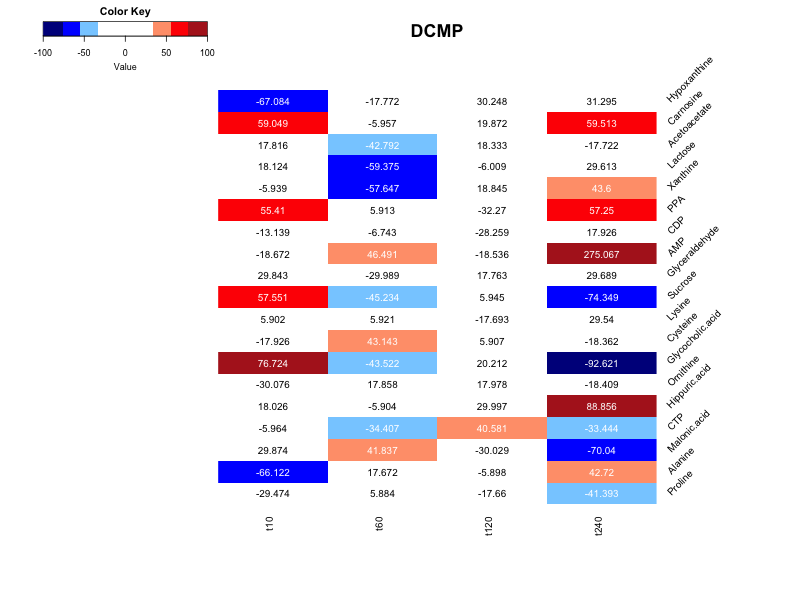

Supplement: S1 File — The R-based computational framework for data preprocessing, metabolite subset selection and dynamic network construction consists of the following R-scripts and text files: 1. Main.R: Main script for the analysis of metabolic data in order to identify putative biomarker candidates based on dynamic network visualization. 2. Preprocessing.R: (i) Removes metabolites with more than 60% of the values missing from the dataset; (ii) Replaces missing values with the metabolite's median at a given time point for the remaining dataset; (iii) Creates a data subset, containing only those metabolites, which are present at all time points. 3. BI.R: Function that calculates pBI scores for all metabolites. 4. InferBIGraph.R: Function to sum up the function calls for the calculation of a network graph. 5. FunctionsGraph.R: Multiple functions for network graph construction and visualization (i.e. create boxplot diagrams with different thresholds, graph calculation, adapt ratio for heatmap construction, plot heatmaps, plot graphs, calculate discrete weights, calculate degree-based weights, calculate graph object, plot pBI scores as bar charts). 6. *.txt files: Contain coordinates for graph visualization. For further information please read the “ReadMe.txt” file in the Supporting information. (ZIP) [file pone.0208953.s004.zip › S1_File/output/Example_Plots/03_Heatmaps/Metabolite DCMP.png]

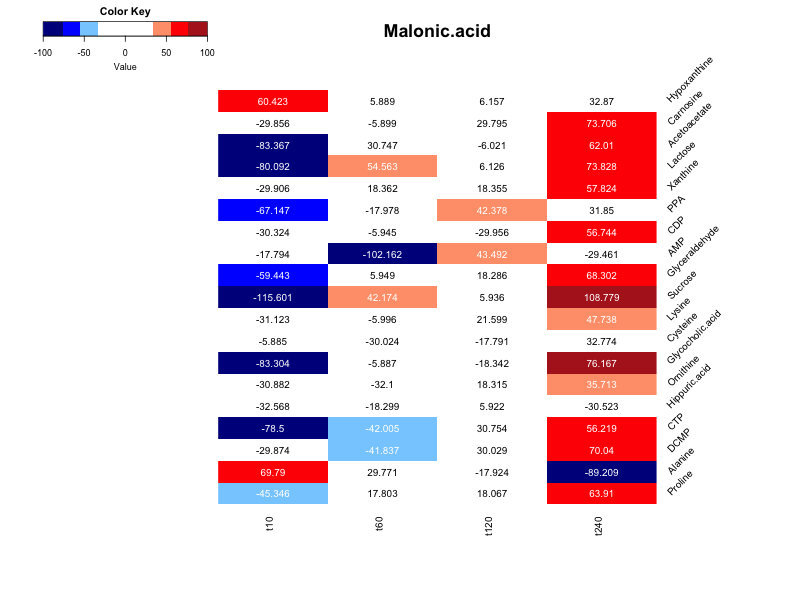

Supplement: S1 File — The R-based computational framework for data preprocessing, metabolite subset selection and dynamic network construction consists of the following R-scripts and text files: 1. Main.R: Main script for the analysis of metabolic data in order to identify putative biomarker candidates based on dynamic network visualization. 2. Preprocessing.R: (i) Removes metabolites with more than 60% of the values missing from the dataset; (ii) Replaces missing values with the metabolite's median at a given time point for the remaining dataset; (iii) Creates a data subset, containing only those metabolites, which are present at all time points. 3. BI.R: Function that calculates pBI scores for all metabolites. 4. InferBIGraph.R: Function to sum up the function calls for the calculation of a network graph. 5. FunctionsGraph.R: Multiple functions for network graph construction and visualization (i.e. create boxplot diagrams with different thresholds, graph calculation, adapt ratio for heatmap construction, plot heatmaps, plot graphs, calculate discrete weights, calculate degree-based weights, calculate graph object, plot pBI scores as bar charts). 6. *.txt files: Contain coordinates for graph visualization. For further information please read the “ReadMe.txt” file in the Supporting information. (ZIP) [file pone.0208953.s004.zip › S1_File/output/Example_Plots/03_Heatmaps/Metabolite Malonic.acid.png]

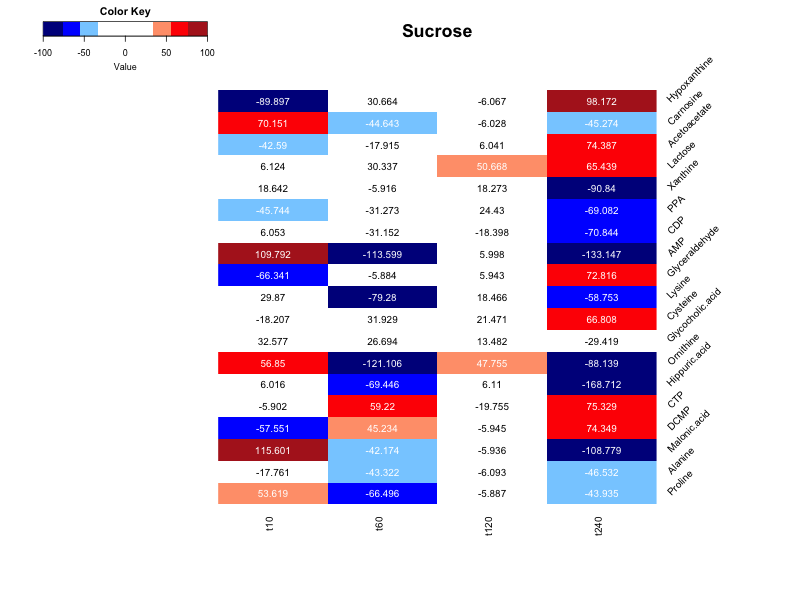

Supplement: S1 File — The R-based computational framework for data preprocessing, metabolite subset selection and dynamic network construction consists of the following R-scripts and text files: 1. Main.R: Main script for the analysis of metabolic data in order to identify putative biomarker candidates based on dynamic network visualization. 2. Preprocessing.R: (i) Removes metabolites with more than 60% of the values missing from the dataset; (ii) Replaces missing values with the metabolite's median at a given time point for the remaining dataset; (iii) Creates a data subset, containing only those metabolites, which are present at all time points. 3. BI.R: Function that calculates pBI scores for all metabolites. 4. InferBIGraph.R: Function to sum up the function calls for the calculation of a network graph. 5. FunctionsGraph.R: Multiple functions for network graph construction and visualization (i.e. create boxplot diagrams with different thresholds, graph calculation, adapt ratio for heatmap construction, plot heatmaps, plot graphs, calculate discrete weights, calculate degree-based weights, calculate graph object, plot pBI scores as bar charts). 6. *.txt files: Contain coordinates for graph visualization. For further information please read the “ReadMe.txt” file in the Supporting information. (ZIP) [file pone.0208953.s004.zip › S1_File/output/Example_Plots/03_Heatmaps/Metabolite Sucrose.png]

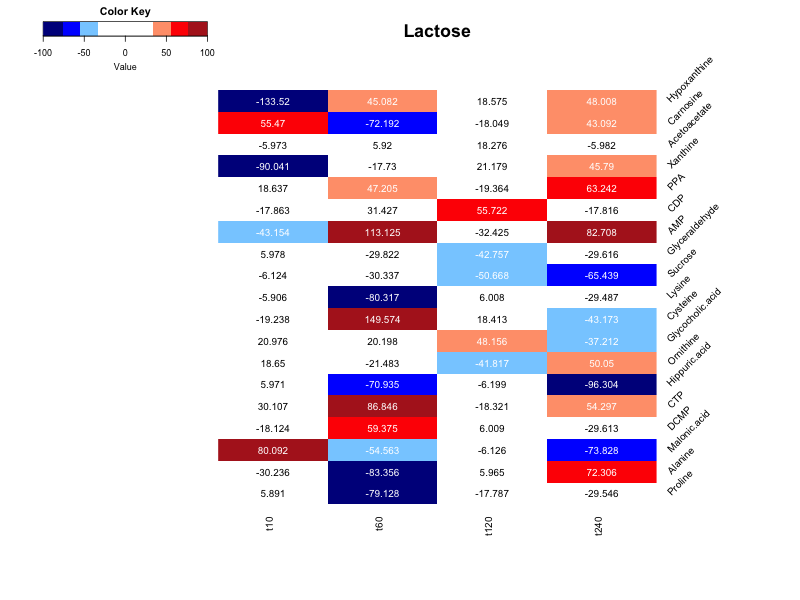

Supplement: S1 File — The R-based computational framework for data preprocessing, metabolite subset selection and dynamic network construction consists of the following R-scripts and text files: 1. Main.R: Main script for the analysis of metabolic data in order to identify putative biomarker candidates based on dynamic network visualization. 2. Preprocessing.R: (i) Removes metabolites with more than 60% of the values missing from the dataset; (ii) Replaces missing values with the metabolite's median at a given time point for the remaining dataset; (iii) Creates a data subset, containing only those metabolites, which are present at all time points. 3. BI.R: Function that calculates pBI scores for all metabolites. 4. InferBIGraph.R: Function to sum up the function calls for the calculation of a network graph. 5. FunctionsGraph.R: Multiple functions for network graph construction and visualization (i.e. create boxplot diagrams with different thresholds, graph calculation, adapt ratio for heatmap construction, plot heatmaps, plot graphs, calculate discrete weights, calculate degree-based weights, calculate graph object, plot pBI scores as bar charts). 6. *.txt files: Contain coordinates for graph visualization. For further information please read the “ReadMe.txt” file in the Supporting information. (ZIP) [file pone.0208953.s004.zip › S1_File/output/Example_Plots/03_Heatmaps/Metabolite Lactose.png]

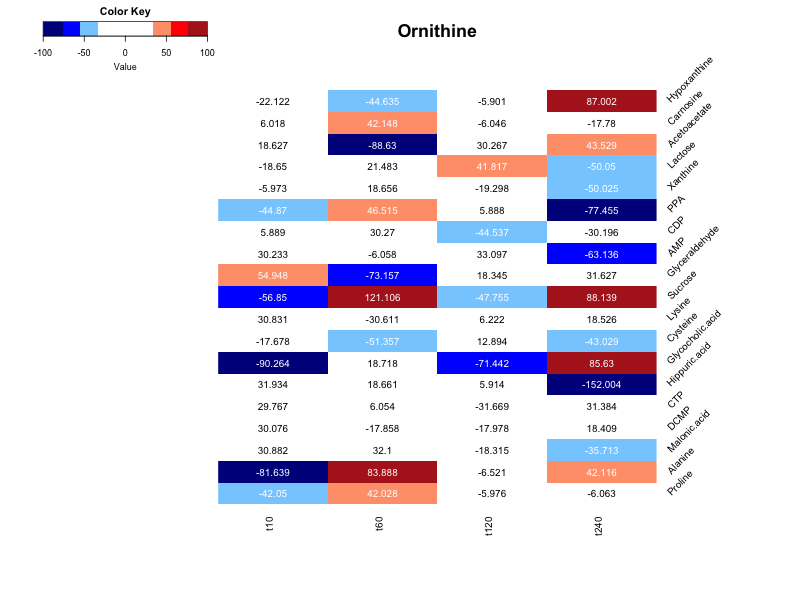

Supplement: S1 File — The R-based computational framework for data preprocessing, metabolite subset selection and dynamic network construction consists of the following R-scripts and text files: 1. Main.R: Main script for the analysis of metabolic data in order to identify putative biomarker candidates based on dynamic network visualization. 2. Preprocessing.R: (i) Removes metabolites with more than 60% of the values missing from the dataset; (ii) Replaces missing values with the metabolite's median at a given time point for the remaining dataset; (iii) Creates a data subset, containing only those metabolites, which are present at all time points. 3. BI.R: Function that calculates pBI scores for all metabolites. 4. InferBIGraph.R: Function to sum up the function calls for the calculation of a network graph. 5. FunctionsGraph.R: Multiple functions for network graph construction and visualization (i.e. create boxplot diagrams with different thresholds, graph calculation, adapt ratio for heatmap construction, plot heatmaps, plot graphs, calculate discrete weights, calculate degree-based weights, calculate graph object, plot pBI scores as bar charts). 6. *.txt files: Contain coordinates for graph visualization. For further information please read the “ReadMe.txt” file in the Supporting information. (ZIP) [file pone.0208953.s004.zip › S1_File/output/Example_Plots/03_Heatmaps/Metabolite Ornithine.png]

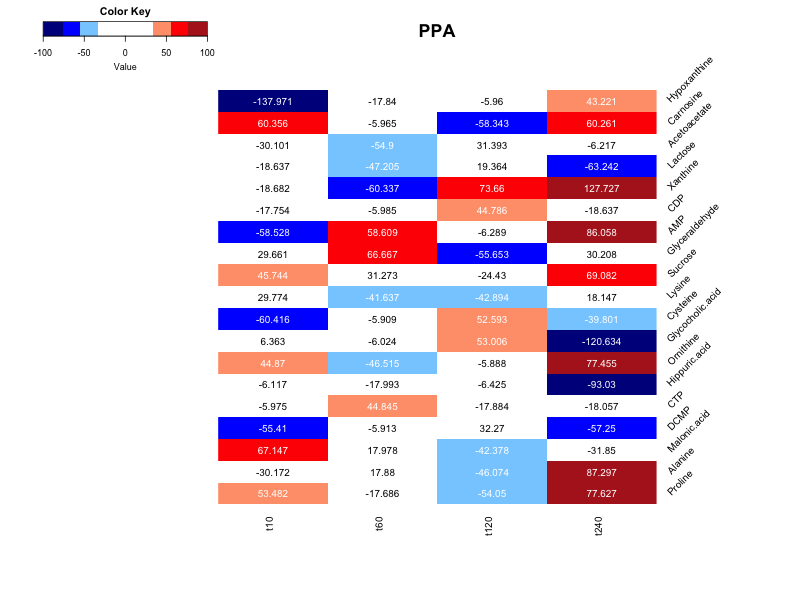

Supplement: S1 File — The R-based computational framework for data preprocessing, metabolite subset selection and dynamic network construction consists of the following R-scripts and text files: 1. Main.R: Main script for the analysis of metabolic data in order to identify putative biomarker candidates based on dynamic network visualization. 2. Preprocessing.R: (i) Removes metabolites with more than 60% of the values missing from the dataset; (ii) Replaces missing values with the metabolite's median at a given time point for the remaining dataset; (iii) Creates a data subset, containing only those metabolites, which are present at all time points. 3. BI.R: Function that calculates pBI scores for all metabolites. 4. InferBIGraph.R: Function to sum up the function calls for the calculation of a network graph. 5. FunctionsGraph.R: Multiple functions for network graph construction and visualization (i.e. create boxplot diagrams with different thresholds, graph calculation, adapt ratio for heatmap construction, plot heatmaps, plot graphs, calculate discrete weights, calculate degree-based weights, calculate graph object, plot pBI scores as bar charts). 6. *.txt files: Contain coordinates for graph visualization. For further information please read the “ReadMe.txt” file in the Supporting information. (ZIP) [file pone.0208953.s004.zip › S1_File/output/Example_Plots/03_Heatmaps/Metabolite PPA.png]

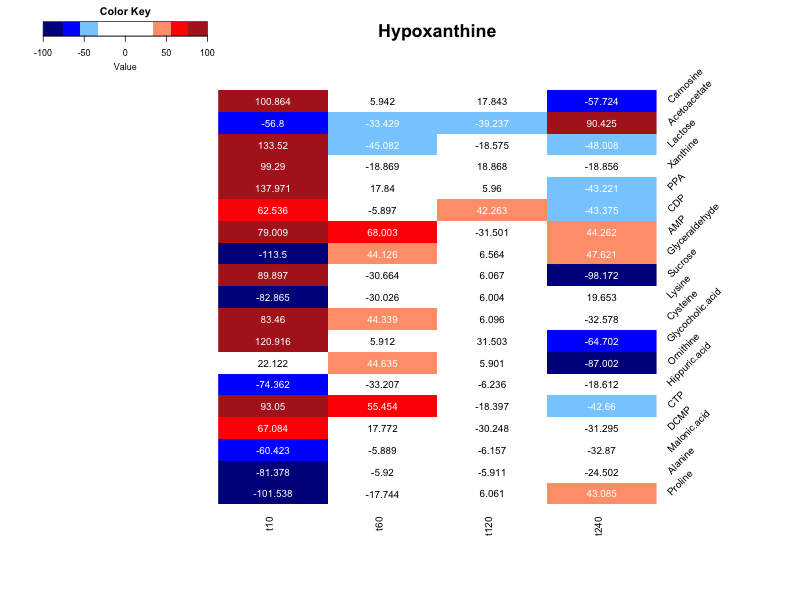

Supplement: S1 File — The R-based computational framework for data preprocessing, metabolite subset selection and dynamic network construction consists of the following R-scripts and text files: 1. Main.R: Main script for the analysis of metabolic data in order to identify putative biomarker candidates based on dynamic network visualization. 2. Preprocessing.R: (i) Removes metabolites with more than 60% of the values missing from the dataset; (ii) Replaces missing values with the metabolite's median at a given time point for the remaining dataset; (iii) Creates a data subset, containing only those metabolites, which are present at all time points. 3. BI.R: Function that calculates pBI scores for all metabolites. 4. InferBIGraph.R: Function to sum up the function calls for the calculation of a network graph. 5. FunctionsGraph.R: Multiple functions for network graph construction and visualization (i.e. create boxplot diagrams with different thresholds, graph calculation, adapt ratio for heatmap construction, plot heatmaps, plot graphs, calculate discrete weights, calculate degree-based weights, calculate graph object, plot pBI scores as bar charts). 6. *.txt files: Contain coordinates for graph visualization. For further information please read the “ReadMe.txt” file in the Supporting information. (ZIP) [file pone.0208953.s004.zip › S1_File/output/Example_Plots/03_Heatmaps/Metabolite Hypoxanthine.png]

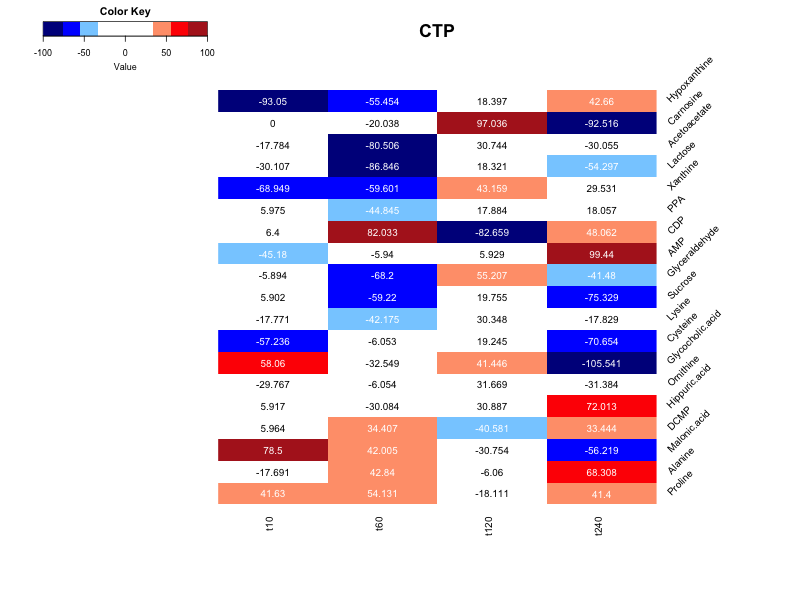

Supplement: S1 File — The R-based computational framework for data preprocessing, metabolite subset selection and dynamic network construction consists of the following R-scripts and text files: 1. Main.R: Main script for the analysis of metabolic data in order to identify putative biomarker candidates based on dynamic network visualization. 2. Preprocessing.R: (i) Removes metabolites with more than 60% of the values missing from the dataset; (ii) Replaces missing values with the metabolite's median at a given time point for the remaining dataset; (iii) Creates a data subset, containing only those metabolites, which are present at all time points. 3. BI.R: Function that calculates pBI scores for all metabolites. 4. InferBIGraph.R: Function to sum up the function calls for the calculation of a network graph. 5. FunctionsGraph.R: Multiple functions for network graph construction and visualization (i.e. create boxplot diagrams with different thresholds, graph calculation, adapt ratio for heatmap construction, plot heatmaps, plot graphs, calculate discrete weights, calculate degree-based weights, calculate graph object, plot pBI scores as bar charts). 6. *.txt files: Contain coordinates for graph visualization. For further information please read the “ReadMe.txt” file in the Supporting information. (ZIP) [file pone.0208953.s004.zip › S1_File/output/Example_Plots/03_Heatmaps/Metabolite CTP.png]

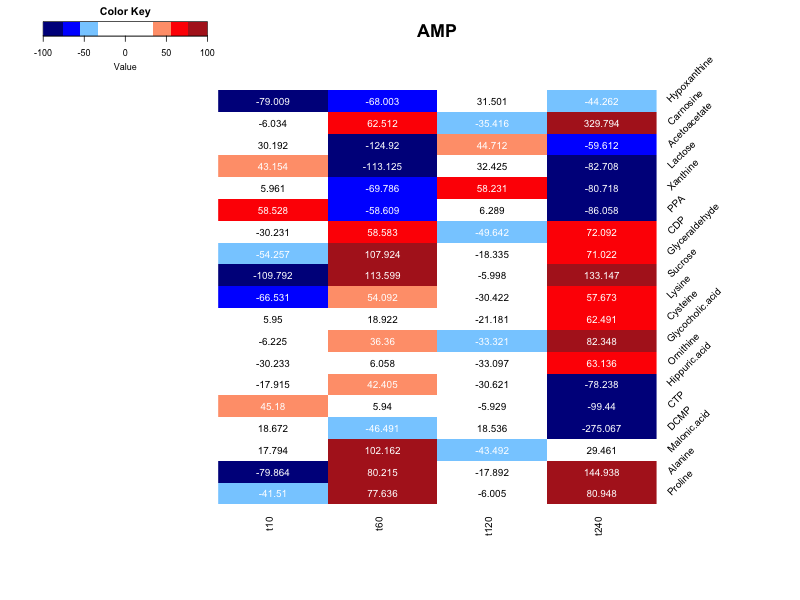

Supplement: S1 File — The R-based computational framework for data preprocessing, metabolite subset selection and dynamic network construction consists of the following R-scripts and text files: 1. Main.R: Main script for the analysis of metabolic data in order to identify putative biomarker candidates based on dynamic network visualization. 2. Preprocessing.R: (i) Removes metabolites with more than 60% of the values missing from the dataset; (ii) Replaces missing values with the metabolite's median at a given time point for the remaining dataset; (iii) Creates a data subset, containing only those metabolites, which are present at all time points. 3. BI.R: Function that calculates pBI scores for all metabolites. 4. InferBIGraph.R: Function to sum up the function calls for the calculation of a network graph. 5. FunctionsGraph.R: Multiple functions for network graph construction and visualization (i.e. create boxplot diagrams with different thresholds, graph calculation, adapt ratio for heatmap construction, plot heatmaps, plot graphs, calculate discrete weights, calculate degree-based weights, calculate graph object, plot pBI scores as bar charts). 6. *.txt files: Contain coordinates for graph visualization. For further information please read the “ReadMe.txt” file in the Supporting information. (ZIP) [file pone.0208953.s004.zip › S1_File/output/Example_Plots/03_Heatmaps/Metabolite AMP.png]

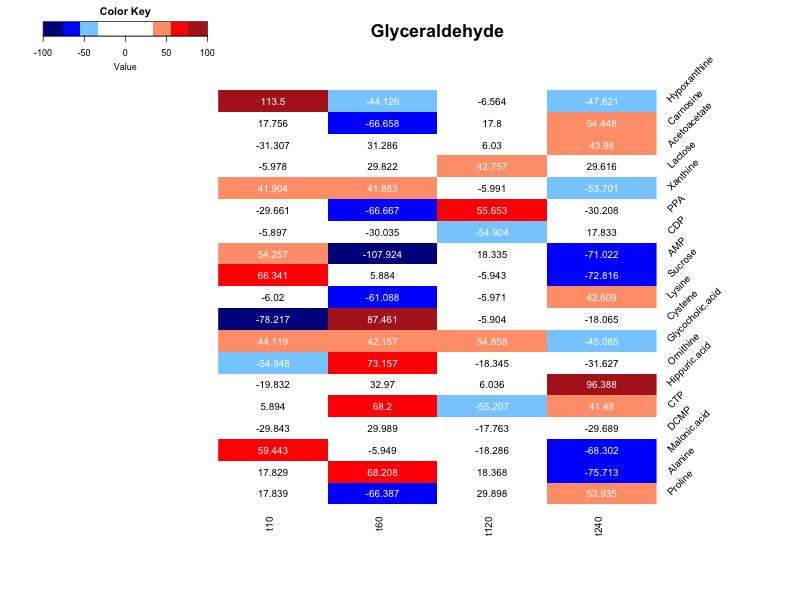

Supplement: S1 File — The R-based computational framework for data preprocessing, metabolite subset selection and dynamic network construction consists of the following R-scripts and text files: 1. Main.R: Main script for the analysis of metabolic data in order to identify putative biomarker candidates based on dynamic network visualization. 2. Preprocessing.R: (i) Removes metabolites with more than 60% of the values missing from the dataset; (ii) Replaces missing values with the metabolite's median at a given time point for the remaining dataset; (iii) Creates a data subset, containing only those metabolites, which are present at all time points. 3. BI.R: Function that calculates pBI scores for all metabolites. 4. InferBIGraph.R: Function to sum up the function calls for the calculation of a network graph. 5. FunctionsGraph.R: Multiple functions for network graph construction and visualization (i.e. create boxplot diagrams with different thresholds, graph calculation, adapt ratio for heatmap construction, plot heatmaps, plot graphs, calculate discrete weights, calculate degree-based weights, calculate graph object, plot pBI scores as bar charts). 6. *.txt files: Contain coordinates for graph visualization. For further information please read the “ReadMe.txt” file in the Supporting information. (ZIP) [file pone.0208953.s004.zip › S1_File/output/Example_Plots/03_Heatmaps/Metabolite Glyceraldehyde.png]

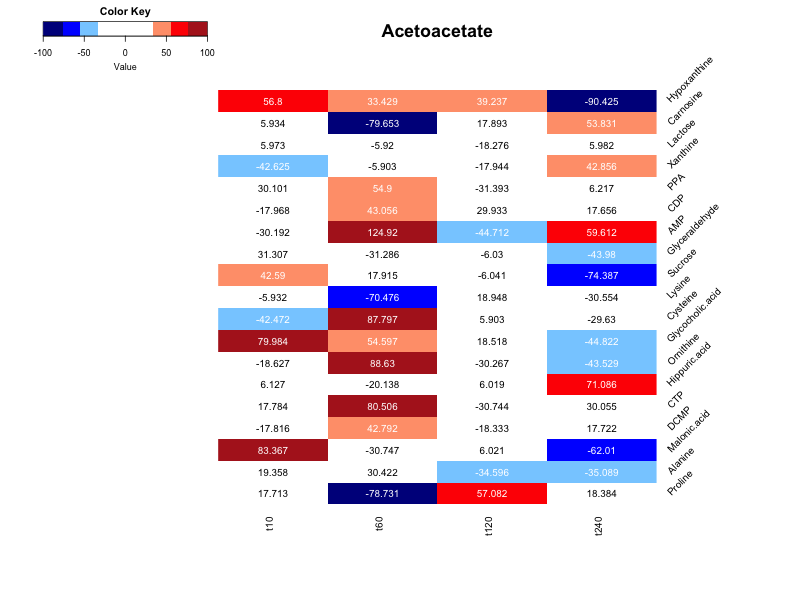

Supplement: S1 File — The R-based computational framework for data preprocessing, metabolite subset selection and dynamic network construction consists of the following R-scripts and text files: 1. Main.R: Main script for the analysis of metabolic data in order to identify putative biomarker candidates based on dynamic network visualization. 2. Preprocessing.R: (i) Removes metabolites with more than 60% of the values missing from the dataset; (ii) Replaces missing values with the metabolite's median at a given time point for the remaining dataset; (iii) Creates a data subset, containing only those metabolites, which are present at all time points. 3. BI.R: Function that calculates pBI scores for all metabolites. 4. InferBIGraph.R: Function to sum up the function calls for the calculation of a network graph. 5. FunctionsGraph.R: Multiple functions for network graph construction and visualization (i.e. create boxplot diagrams with different thresholds, graph calculation, adapt ratio for heatmap construction, plot heatmaps, plot graphs, calculate discrete weights, calculate degree-based weights, calculate graph object, plot pBI scores as bar charts). 6. *.txt files: Contain coordinates for graph visualization. For further information please read the “ReadMe.txt” file in the Supporting information. (ZIP) [file pone.0208953.s004.zip › S1_File/output/Example_Plots/03_Heatmaps/Metabolite Acetoacetate.png]
